# Supplementary material for: How do pilot and feasibility studies inform randomised placebo-controlled trials in surgery? A systematic review
Source: BMJ Open. 2023 Nov 20;13(11):e071094. doi: 10.1136/bmjopen-2022-071094 (PMC10660967; doi:10.1136/bmjopen-2022-071094)
Supplement: Supplementary data [file bmjopen-2022-071094supp001.pdf]

Appendix A – Search terms uses in Ovid MEDLINE, Ovid EMBASE and CENTRAL databases

Ovid MEDLINE

1. Clinical trial/
2. Randomized controlled trial/
3. Randomization/
4. Rct.tw.
5. random allocation.tw.
6. Randomly allocated.tw.
7. Allocated randomly.tw.
8. Randomized Controlled Trials as Topic/
9. randomized controlled trial/
10. Double Blind Method/
11. Single Blind Method/
12. clinical trial/
13. controlled clinical trial.pt.
14. randomized controlled trial.pt.
15. clinical trial.pt.
16. exp Clinical Trials as topic/
17. or/1-16
18. PLACEBOS/
19. placebo$.tw.
20. sham.tw.
21. immitation.tw.
22. placebo effect$.tw.
23. or/18-22
24. surgery.tw.
25. surgical.tw.
26. arthroscopy.tw.
27. endoscopy.tw.
28. transplantation.tw.
29. $scopy.tw.

30. $scopic.tw.
31. laparoscopy.tw.
32. Meta-Analysis as Topic/
33. meta analy$.tw.
34. metaanaly$.tw.
35. Review/
36. Comment/
37. Letter/
38. Editorial/
39. animal/
40. dose$.tw.
41. pre$medication.tw.
42. an$esthesia.tw.
43. an$esthetic$.tw.
44. antibiotic$.tw.
45. steroid$.tw.
46. prophylaxis.tw.
47. prevention.tw.
48. preoperative.tw.
49. preanaesthetic$.tw.
50. pre$emptive.tw.
51. pre-operative.tw.
52. post-operative.tw.
53. postoperative.tw.
54. post$surgery.tw.
55. (analgesic adj trial).tw.
56. oral$.tw.
57. acupuncture.tw.
58. acupressure.tw.
59. scar.tw.
60. infection.tw.

61. dental.tw.

62. post$surgical.tw.

63. pre$surgical.tw.

64. case report.tw.

65. case study.tw.

66. pacing.tw.

67. stimulation.tw.

68. growth factor$.tw.

69. hormon$.tw.

70. or/24-31

71. or/32-69

72. 17 and 23

73. 72 and 70

74. 73 not 71

Ovid EMBASE

1. Clinical trial/

2. Randomized controlled trial/

3. Randomization/

4. Single blind procedure/

5. Double blind procedure/

6. Crossover procedure/

7. Randomi?ed controlled trial$.tw.

8. Rct.tw.

9. random allocation.tw.

10. Randomly allocated.tw.

11. Allocated randomly.tw.

12. (allocated adj2 random).tw.

13. Single blind$.tw.

14. Single blind$.tw.

15. or/1-14

16. Placebo$.tw.

17. placebo effect$.tw.
18. sham.tw.
19. placebo.tw.
20. or/16-19
21. surgery.tw.
22. surgical.tw.
23. arthroscopy.tw.
24. endoscopy.tw.
25. $scopy.tw.
26. $scopic.tw.
27. laparoscopy.tw.
28. transplantation.tw.
29. or/21-28
30. letter/
31. Review/
32. animal/
33. editorial/
34. ((meta adj analy$) or metaanalys$).tw.
35. (analgesic adj trial).tw.
36. meta$analysis.tw.
37. dose$.tw.
38. oral$.tw.
39. orally.tw.
40. dental.tw.
41. pre$medication.tw.
42. pre$surgical.tw.
43. post$surgical.tw.
44. pre$surgery.tw.
45. post$surgery.tw.
46. antibiotic$.tw.
47. an$esthetic$.tw.

48. steroid$.tw.

49. peri$operative.tw.

50. pre$emptive.tw.

51. preanesthetic$.tw.

52. post$operative.tw.

53. prophylaxis.tw.

54. prevention.tw.

55. acupuncture.tw.

56. accupressure.tw.

57. scar$.tw.

58. infection$.tw.

59. acupressure.tw.

60. pre$operative.tw.

61. growth factor$.tw.

62. pacing.tw.

63. stimulation.tw.

64. hormon$.tw.

65. case report$.tw.

66. case study.tw.

67. or/30-66

68. 15 and 20

69. 68 and 29

70. 69 not 67

Cochrane Central Register of Controlled Trials

http://onlinelibrary.wiley.com/o/cochrane/cochrane_clcentral_articles_fs.html

(placebo OR placebo effect OR sham OR imitation):ti,ab,kw and (surgery OR surgical OR laparoscopy OR endoscopy

OR arthroscopy OR transplantation OR scopy):ti,ab,kw and (clinical trial OR randomised clinical trail OR RCT OR

randomised controlled trial OR randomisation):ti,ab,kw not (drug OR dental OR oral OR infection OR steroids OR

hormones OR growth factor OR prophylaxis OR anaesthesia OR pre-surgical OR post-surgical OR pre-emptive OR

post-operative OR preoperative OR antibiotics OR acupuncture OR acupressure OR scar OR infection OR

prevention):ti,ab,kw not (review OR animal OR stimulation):ti,ab,kw in Trials
