# Supplementary material for: How do pilot and feasibility studies inform randomised placebo-controlled trials in surgery? A systematic review
Source: BMJ Open. 2023 Nov 20;13(11):e071094. doi: 10.1136/bmjopen-2022-071094 (PMC10660967; doi:10.1136/bmjopen-2022-071094)
Supplement: Supplementary data [file bmjopen-2022-071094supp002.pdf]

Supplementary table 2. List of references of 131 included RCTs

Trial #	References (alphabetical order)
1	Abbott, J., Hawe, J., Hunter, D., Holmes, M., Finn, P., & Garry, R. (2004). Laparoscopic excision of endometriosis: a randomized, placebo-controlled trial. Fertility and sterility, 82(4), 878–884. https://doi.org/10.1016/j.fertnstert.2004.03.046
2	Al-Lamee, R., Thompson, D., Dehbi, H. M., Sen, S., Tang, K., Davies, J., Keeble, T., Mielewczik, M., Kaprielian, R., Malik, I. S., Nijjer, S. S., Petraco, R., Cook, C., Ahmad, Y., Howard, J., Baker, C., Sharp, A., Gerber, R., Talwar, S., Assomull, R., … ORBITA investigators (2018). Percutaneous coronary intervention in stable angina (ORBITA): a double-blind, randomised controlled trial. Lancet (London, England), 391(10115), 31–40. https://doi.org/10.1016/S0140-6736(17)32714-9
3	Anderson, D. B., Ferreira, M. L., Harris, I. A., Davis, G. A., Stanford, R., Beard, D., Li, Q., Jan, S., Mobbs, R. J., Maher, C. G., Yong, R., Zammit, T., Latimer, J., & Buchbinder, R. (2019). SUcceSS, SUrgery for Spinal Stenosis: protocol of a randomised, placebo-controlled trial. BMJ open, 9(2), e024944. https://doi.org/10.1136/bmjopen-2018-024944
4	Arts, J., Bisschops, R., Blondeau, K., Farré, R., Vos, R., Holvoet, L., Caenepeel, P., Lerut, A., & Tack, J. (2012). A double-blind sham-controlled study of the effect of radiofrequency energy on symptoms and distensibility of the gastro-esophageal junction in GERD. The American journal of gastroenterology, 107(2), 222–230. https://doi.org/10.1038/ajg.2011.395
5	Bäck, L. J., Liukko, T., Rantanen, I., Peltola, J. S., Partinen, M., Ylikoski, J., & Mäkitie, A. A. (2009). Radiofrequency surgery of the soft palate in the treatment of mild obstructive sleep apnea is not effective as a single-stage procedure: A randomized single-blinded placebo-controlled trial. The Laryngoscope, 119(8), 1621–1627. https://doi.org/10.1002/lary.20562
6	Bajbouj, M., Becker, V., Eckel, F., Miehlke, S., Pech, O., Prinz, C., Schmid, R. M., & Meining, A. (2009). Argon plasma coagulation of cervical heterotopic gastric mucosa as an alternative treatment for globus sensations. Gastroenterology, 137(2), 440–444. https://doi.org/10.1053/j.gastro.2009.04.053
7	Beard, D. J., Rees, J. L., Cook, J. A., Rombach, I., Cooper, C., Merritt, N., Shirkey, B. A., Donovan, J. L., Gwilym, S., Savulescu, J., Moser, J., Gray, A., Jepson, M., Tracey, I., Judge, A., Wartolowska, K., Carr, A. J., & CSAW Study Group (2018). Arthroscopic subacromial decompression for subacromial shoulder pain (CSAW): a multicentre, pragmatic, parallel group, placebo-controlled, three-group, randomised surgical trial. Lancet (London, England), 391(10118), 329–338. https://doi.org/10.1016/S0140-6736(17)32457-1
8	Benjamin, S. B., Maher, K. A., Cattau, E. L., Jr, Collen, M. J., Fleischer, D. E., Lewis, J. H., Ciarleglio, C. A., Earll, J. M., Schaffer, S., & Mirkin, K. (1988). Double-blind controlled trial of the Garren-Edwards gastric bubble: an adjunctive treatment for exogenous obesity. Gastroenterology, 95(3), 581–588. https://doi.org/10.1016/s0016-5085(88)80001-5
9	Blaganje, M., Šćepanović, D., Žgur, L., Verdenik, I., Pajk, F., & Lukanović, A. (2018). Non-ablative Er:YAG laser therapy effect on stress urinary incontinence related to quality of life and sexual function: A randomized controlled trial. European journal of obstetrics, gynecology, and reproductive biology, 224, 153–158. https://doi.org/10.1016/j.ejogrb.2018.03.038
10	Böhm, M., Kario, K., Kandzari, D. E., Mahfoud, F., Weber, M. A., Schmieder, R. E., Tsioufis, K., Pocock, S., Konstantinidis, D., Choi, J. W., East, C., Lee, D. P., Ma, A., Ewen, S., Cohen, D. L., Wilensky, R., Devireddy, C. M., Lea, J., Schmid, A., Weil, J., … SPYRAL HTN-OFF MED Pivotal Investigators (2020). Efficacy of catheter-based renal denervation in the absence of antihypertensive medications (SPYRAL HTN-OFF MED Pivotal): a multicentre, randomised, sham-controlled trial. Lancet (London, England), 395(10234), 1444–1451. https://doi.org/10.1016/S0140-6736(20)30554-7

11	Bradley, J. D., Heilman, D. K., Katz, B. P., Gsell, P., Wallick, J. E., & Brandt, K. D. (2002). Tidal irrigation as treatment for knee osteoarthritis: a sham-controlled, randomized, double-blinded evaluation. Arthritis and rheumatism, 46(1), 100–108. https://doi.org/10.1002/1529-0131(200201)46:1<100::aid-art10037>3.0.co;2-v
12	Buchbinder, R., Osborne, R. H., Ebeling, P. R., Wark, J. D., Mitchell, P., Wriedt, C., Graves, S., Staples, M. P., & Murphy, B. (2009). A randomized trial of vertebroplasty for painful osteoporotic vertebral fractures. The New England journal of medicine, 361(6), 557–568. https://doi.org/10.1056/NEJMoa0900429
13	Campbell, M. K., Entwistle, V. A., Cuthbertson, B. H., Skea, Z. C., Sutherland, A. G., McDonald, A. M., Norrie, J. D., Carlson, R. V., Bridgman, S., & KORAL study group (2011). Developing a placebo-controlled trial in surgery: issues of design, acceptability and feasibility. Trials, 12, 50. https://doi.org/10.1186/1745-6215-12-50
14	Castro, M., Rubin, A. S., Laviolette, M., Fiterman, J., De Andrade Lima, M., Shah, P. L., Fiss, E., Olivenstein, R., Thomson, N. C., Niven, R. M., Pavord, I. D., Simoff, M., Duhamel, D. R., McEvoy, C., Barbers, R., Ten Hacken, N. H., Wechsler, M. E., Holmes, M., Phillips, M. J., Erzurum, S., … AIR2 Trial Study Group (2010). Effectiveness and safety of bronchial thermoplasty in the treatment of severe asthma: a multicenter, randomized, double-blind, sham-controlled clinical trial. American journal of respiratory and critical care medicine, 181(2), 116–124. https://doi.org/10.1164/rccm.200903-0354OC
15	Chan, D. L., Cruz, J. R., Mui, W. L., Wong, S. K. H., & Ng, E. K. W. (2021). Outcomes with Intra-gastric Balloon Therapy in BMI < 35 Non-morbid Obesity: 10-Year Follow-Up Study of an RCT. Obesity surgery, 31(2), 781–786. https://doi.org/10.1007/s11695-020-04986-3
16	Clark, W., Bird, P., Gonski, P., Diamond, T. H., Smerdely, P., McNeil, H. P., Schlaphoff, G., Bryant, C., Barnes, E., & Gebski, V. (2016). Safety and efficacy of vertebroplasty for acute painful osteoporotic fractures (VAPOUR): a multicentre, randomised, double-blind, placebo-controlled trial. Lancet (London, England), 388(10052), 1408–1416. https://doi.org/10.1016/S0140-6736(16)31341-1
17	Cobb, L. A., Thomas, G. I., Dillard, D. H., Merendino, K. A., & Bruce, R. A. (1959). An evaluation of internal-mammary-artery ligation by a double-blind technic. The New England journal of medicine, 260(22), 1115–1118. https://doi.org/10.1056/NEJM195905282602204
18	Corley, D. A., Katz, P., Wo, J. M., Stefan, A., Patti, M., Rothstein, R., Edmundowicz, S., Kline, M., Mason, R., & Wolfe, M. M. (2003). Improvement of gastroesophageal reflux symptoms after radiofrequency energy: a randomized, sham-controlled trial. Gastroenterology, 125(3), 668–676. https://doi.org/10.1016/s0016-5085(03)01052-7
19	Cotton, P. B., Durkalski, V., Romagnuolo, J., Pauls, Q., Fogel, E., Tarnasky, P., Aliperti, G., Freeman, M., Kozarek, R., Jamidar, P., Wilcox, M., Serrano, J., Brawman-Mintzer, O., Elta, G., Mauldin, P., Thornhill, A., Hawes, R., Wood-Williams, A., Orrell, K., Drossman, D., … Robuck, P. (2014). Effect of endoscopic sphincterotomy for suspected sphincter of Oddi dysfunction on pain-related disability following cholecystectomy: the EPISOD randomized clinical trial. JAMA, 311(20), 2101–2109. https://doi.org/10.1001/jama.2014.5220
20	Davey, C., Zoumot, Z., Jordan, S., McNulty, W. H., Carr, D. H., Hind, M. D., Hansell, D. M., Rubens, M. B., Banya, W., Polkey, M. I., Shah, P. L., & Hopkinson, N. S. (2015). Bronchoscopic lung volume reduction with endobronchial valves for patients with heterogeneous emphysema and intact interlobar fissures (the BeLieVeR-HIFi study): a randomised controlled trial. Lancet (London, England), 386(9998), 1066–1073. https://doi.org/10.1016/S0140-6736(15)60001-0
21	Davys, H. J., Turner, D. E., Helliwell, P. S., Conaghan, P. G., Emery, P., & Woodburn, J. (2005). Debridement of plantar callosities in rheumatoid arthritis: a randomized controlled trial. Rheumatology (Oxford, England), 44(2), 207–210. https://doi.org/10.1093/rheumatology/keh435
22	Dawes, P. T., Kirlew, C., & Haslock, I. (1987). Saline washout for knee osteoarthritis: results of a controlled study. Clinical rheumatology, 6(1), 61–63. https://doi.org/10.1007/BF02201002

23	de Quadros, L. G., Neto, M. G., Marchesini, J. C., Teixeira, A., Grecco, E., Junior, R. L. K., Zundel, N., Filho, I. J. Z., de Souza, T. F., Filho, A. C., da Silva, L. B., Ramos, A. C., Ferraz, Á. A. B., & Campos, J. M. (2020). Endoscopic Argon Plasma Coagulation vs. Multidisciplinary Evaluation in the Management of Weight Regain After Gastric Bypass Surgery: a Randomized Controlled Trial with SHAM Group. Obesity surgery, 30(5), 1904–1916. https://doi.org/10.1007/s11695-020-04414-6
24	Devière, J., Costamagna, G., Neuhaus, H., Voderholzer, W., Louis, H., Tringali, A., Marchese, M., Fiedler, T., Darb-Esfahani, P., & Schumacher, B. (2005). Nonresorbable copolymer implantation for gastroesophageal reflux disease: a randomized sham-controlled multicenter trial. Gastroenterology, 128(3), 532–540. https://doi.org/10.1053/j.gastro.2004.12.005
25	Dimond, E. G., Kittle, C. F., & Crockett, J. E. (1960). Comparison of internal mammary artery ligation and sham operation for angina pectoris. The American journal of cardiology, 5, 483–486. https://doi.org/10.1016/0002-9149(60)90105-3
26	Donnenfeld, E. D., Holland, E. J., & Solomon, K. D. (2021). Safety and efficacy of nepafenac punctal plug delivery system in controlling postoperative ocular pain and inflammation after cataract surgery. Journal of cataract and refractive surgery, 47(2), 158–164. https://doi.org/10.1097/j.jcrs.0000000000000414
27	Dowson, A., Mullen, M. J., Peatfield, R., Muir, K., Khan, A. A., Wells, C., Lipscombe, S. L., Rees, T., De Giovanni, J. V., Morrison, W. L., Hildick-Smith, D., Elrington, G., Hillis, W. S., Malik, I. S., & Rickards, A. (2008). Migraine Intervention With STARFlex Technology (MIST) trial: a prospective, multicenter, double-blind, sham-controlled trial to evaluate the effectiveness of patent foramen ovale closure with STARFlex septal repair implant to resolve refractory migraine headache. Circulation, 117(11), 1397–1404. https://doi.org/10.1161/CIRCULATIONAHA.107.727271
28	Eid, G. M., McCloskey, C. A., Eagleton, J. K., Lee, L. B., & Courcoulas, A. P. (2014). StomaphyX vs a sham procedure for revisional surgery to reduce regained weight in Roux-en-Y gastric bypass patients: a randomized clinical trial. JAMA surgery, 149(4), 372–379. https://doi.org/10.1001/jamasurg.2013.4051
29	Engholm, M., Bertelsen, J. B., Mathiassen, O. N., Bøtker, H. E., Vase, H., Peters, C. D., Bech, J. N., Buus, N. H., Schroeder, A. P., Rickers, H., Hansen, K. W., Poulsen, P. L., Kaltoft, A., & Christensen, K. L. (2018). Effects of renal denervation on coronary flow reserve and forearm dilation capacity in patients with treatment-resistant hypertension. A randomized, double-blinded, sham-controlled clinical trial. International journal of cardiology, 250, 29–34. https://doi.org/10.1016/j.ijcard.2017.09.200
30	Feldman, T., Mauri, L., Kahwash, R., Litwin, S., Ricciardi, M. J., van der Harst, P., Penicka, M., Fail, P. S., Kaye, D. M., Petrie, M. C., Basuray, A., Hummel, S. L., Forde-McLean, R., Nielsen, C. D., Lilly, S., Massaro, J. M., Burkhoff, D., Shah, S. J., & REDUCE LAP-HF I Investigators and Study Coordinators (2018). Transcatheter Interatrial Shunt Device for the Treatment of Heart Failure With Preserved Ejection Fraction (REDUCE LAP-HF I [Reduce Elevated Left Atrial Pressure in Patients With Heart Failure]): A Phase 2, Randomized, Sham-Controlled Trial. Circulation, 137(4), 364–375. https://doi.org/10.1161/CIRCULATIONAHA.117.032094
31	Firanescu, C. E., de Vries, J., Lodder, P., Venmans, A., Schoemaker, M. C., Smeets, A. J., Donga, E., Juttmann, J. R., Klazen, C. A. H., Elgersma, O. E. H., Jansen, F. H., Tielbeek, A. V., Boukrab, I., Schonenberg, K., van Rooij, W. J. J., Hirsch, J. A., & Lohle, P. N. M. (2018). Vertebroplasty versus sham procedure for painful acute osteoporotic vertebral compression fractures (VERTOS IV): randomised sham controlled clinical trial. BMJ (Clinical research ed.), 361, k1551. https://doi.org/10.1136/bmj.k1551<br><br>Firanescu, C. E., de Vries, J., Lodder, P., Schoemaker, M. C., Smeets, A. J., Donga, E., Juttmann, J. R., Klazen, C. A. H., Elgersma, O. E. H., Jansen, F. H., van der Horst, I., Blonk, M., Venmans, A., & Lohle, P. N. M. (2019). Percutaneous Vertebroplasty is no Risk Factor for New Vertebral Fractures and Protects Against Further Height Loss (VERTOS IV). Cardiovascular and interventional radiology, 42(7), 991–1000. https://doi.org/10.1007/s00270-019-02205-w

32	Fischgrund, J. S., Rhyne, A., Franke, J., Sasso, R., Kitchel, S., Bae, H., Yeung, C., Truumees, E., Schaufele, M., Yuan, P., Vajkoczy, P., DePalma, M., Anderson, D. G., Thibodeau, L., & Meyer, B. (2018). Intraosseous basivertebral nerve ablation for the treatment of chronic low back pain: a prospective randomized double-blind sham-controlled multi-center study. European spine journal: official publication of the European Spine Society, the European Spinal Deformity Society, and the European Section of the Cervical Spine Research Society, 27(5), 1146–1156. https://doi.org/10.1007/s00586-018-5496-1
33	Fleischer D. (1985). Endoscopic Nd:YAG laser therapy for active esophageal variceal bleeding. A randomized controlled study. Gastrointestinal endoscopy, 31(1), 4–9. https://doi.org/10.1016/s0016-5107(85)71954-2
34	Fockens, P., Cohen, L., Edmundowicz, S. A., Binmoeller, K., Rothstein, R. I., Smith, D., Lin, E., Nickl, N., Overholt, B., Kahrilas, P. J., Vakil, N., Abdel Aziz Hassan, A. M., & Lehman, G. A. (2010). Prospective randomized controlled trial of an injectable esophageal prosthesis versus a sham procedure for endoscopic treatment of gastroesophageal reflux disease. Surgical endoscopy, 24(6), 1387–1397. https://doi.org/10.1007/s00464-009-0784-9
35	Freed, C. R., Greene, P. E., Breeze, R. E., Tsai, W. Y., DuMouchel, W., Kao, R., Dillon, S., Winfield, H., Culver, S., Trojanowski, J. Q., Eidelberg, D., & Fahn, S. (2001). Transplantation of embryonic dopamine neurons for severe Parkinson's disease. The New England journal of medicine, 344(10), 710–719. https://doi.org/10.1056/NEJM200103083441002
36	Freeman, B. J., Fraser, R. D., Cain, C. M., Hall, D. J., & Chapple, D. C. (2005). A randomized, double-blind, controlled trial: intradiscal electrothermal therapy versus placebo for the treatment of chronic discogenic low back pain. Spine, 30(21), 2369–2378. https://doi.org/10.1097/01.brs.0000186587.43373.f2
37	Freitas, D., Donato, A., & Monteiro, J. G. (1985). Controlled trial of liquid monopolar electrocoagulation in bleeding peptic ulcers. The American journal of gastroenterology, 80(11), 853–857.
38	Friedman, M., Schalch, P., Lin, H. C., Kakodkar, K. A., Joseph, N. J., & Mazloom, N. (2008). Palatal implants for the treatment of snoring and obstructive sleep apnea/hypopnea syndrome. Otolaryngology--head and neck surgery : official journal of American Academy of Otolaryngology-Head and Neck Surgery, 138(2), 209–216. https://doi.org/10.1016/j.otohns.2007.10.026
39	Fullarton, G. M., Birnie, G. G., Macdonald, A., & Murray, W. R. (1989). Controlled trial of heater probe treatment in bleeding peptic ulcers. The British journal of surgery, 76(6), 541–544. https://doi.org/10.1002/bjs.1800760606
40	Garcia, C. A., Soler, F. C. (2018). The Effect of Plantar Hyperkeratosis Debridement on Self-Perception of Pain Levels in older People. International journal of gerontology, 12(4), 314–318. https://doi.org/10.1016/j.ijge.2018.05.002
41	Geenen, J. E., Hogan, W. J., Dodds, W. J., Toouli, J., & Venu, R. P. (1989). The efficacy of endoscopic sphincterotomy after cholecystectomy in patients with sphincter-of-Oddi dysfunction. The New England journal of medicine, 320(2), 82–87. https://doi.org/10.1056/NEJM198901123200203
42	Geliebter, A., Melton, P. M., Gage, D., McCray, R. S., & Hashim, S. A. (1990). Gastric balloon to treat obesity: a double-blind study in nondieting subjects. The American journal of clinical nutrition, 51(4), 584–588. https://doi.org/10.1093/ajcn/51.4.584
43	Genco, A., Cipriano, M., Bacci, V., Cuzzolaro, M., Materia, A., Raparelli, L., Docimo, C., Lorenzo, M., & Basso, N. (2006). BioEnterics Intragastric Balloon (BIB): a short-term, double-blind, randomised, controlled, crossover study on weight reduction in morbidly obese patients. International journal of obesity (2005), 30(1), 129–133. https://doi.org/10.1038/sj.ijo.0803094
44	Gillespie, M. B., Wylie, P. E., Lee-Chiong, T., & Rapoport, D. M. (2011). Effect of palatal implants on continuous positive airway pressure and compliance. Otolaryngology--head and neck surgery: official journal of American Academy of Otolaryngology-Head and Neck Surgery, 144(2), 230–236. https://doi.org/10.1177/0194599810392173

45	Gross, R. E., Watts, R. L., Hauser, R. A., Bakay, R. A., Reichmann, H., von Kummer, R., Ondo, W. G., Reissig, E., Eisner, W., Steiner-Schulze, H., Siedentop, H., Fichte, K., Hong, W., Cornfeldt, M., Beebe, K., Sandbrink, R., & Spheramine Investigational Group (2011). Intrastriatal transplantation of microcarrier-bound human retinal pigment epithelial cells versus sham surgery in patients with advanced Parkinson's disease: a double-blind, randomised, controlled trial. The Lancet. Neurology, 10(6), 509–519. https://doi.org/10.1016/S1474-4422(11)70097-7
46	Guymer, R. H., Wu, Z., Hodgson, L. A. B., Caruso, E., Brassington, K. H., Tindill, N., Aung, K. Z., McGuinness, M. B., Fletcher, E. L., Chen, F. K., Chakravarthy, U., Arnold, J. J., Heriot, W. J., Durkin, S. R., Lek, J. J., Harper, C. A., Wickremasinghe, S. S., Sandhu, S. S., Baglin, E. K., Sharangan, P., … Laser Intervention in Early Stages of Age-Related Macular Degeneration Study Group (2019). Subthreshold Nanosecond Laser Intervention in Age-Related Macular Degeneration: The LEAD Randomized Controlled Clinical Trial. Ophthalmology, 126(6), 829–838. https://doi.org/10.1016/j.ophtha.2018.09.015
47	Guyuron, B., Reed, D., Kriegler, J. S., Davis, J., Pashmini, N., & Amini, S. (2009). A placebo-controlled surgical trial of the treatment of migraine headaches. Plastic and reconstructive surgery, 124(2), 461–468. https://doi.org/10.1097/PRS.0b013e3181adcf6a
48	Håkansson, B., Montgomery, M., Cadiere, G. B., Rajan, A., Bruley des Varannes, S., Lerhun, M., Coron, E., Tack, J., Bischops, R., Thorell, A., Arnelo, U., & Lundell, L. (2015). Randomised clinical trial: transoral incisionless fundoplication vs. sham intervention to control chronic GERD. Alimentary pharmacology & therapeutics, 42(11-12), 1261–1270. https://doi.org/10.1111/apt.13427
49	Hansen, E.J., Simony, A., Carreon, L., Rousing, R., Tropp, H., Andersen, M.O. (2019). Vertebroplasty vs. SHAM for Treating Osteoporotic Vertebral Compression Fractures: A Double Blind RCT. Integrative journal of orthopaedics and traumatology, 2(4), 1–6. https://doi.org/10.31038/IJOT.2019244
50	Harju, T., Kivekäs, I., Numminen, J., & Rautiainen, M. (2018). The effect of inferior turbinate surgery on ear symptoms. The Laryngoscope, 128(3), 568–572. https://doi.org/10.1002/lary.26823
51	Harju, T., Numminen, J., Kivekäs, I., & Rautiainen, M. (2018). A prospective, randomized, placebo-controlled study of inferior turbinate surgery. The Laryngoscope, 128(9), 1997–2003. https://doi.org/10.1002/lary.27103<br><br>Kankaanpää, A., Harju, T., & Numminen, J. (2021). The Effect of Inferior Turbinate Surgery on Quality of Life: A Randomized, Placebo-Controlled Study. Ear, nose, & throat journal, 100(10_suppl), 1107S–1112S. https://doi.org/10.1177/0145561320927944
52	Hartigan P. (1994). Sclerotherapy for male alcoholic cirrhotic patients who have bled from esophageal varices: results of a randomized, multicenter clinical trial. Hepatology, 20(3), 618–625. https://doi.org/10.1002/hep.1840200311
53	Heidari, M., Paknejad, M., Jamali, R., Nokhbatolfoghahaei, H., Fekrazad, R., & Moslemi, N. (2017). Effect of laser photobiomodulation on wound healing and postoperative pain following free gingival graft: A split-mouth triple-blind randomized controlled clinical trial. Journal of photochemistry and photobiology. B, Biology, 172, 109–114. https://doi.org/10.1016/j.jphotobiol.2017.05.022
54	Hersh, P. S., Stulting, R. D., Muller, D., Durrie, D. S., Rajpal, R. K., & U.S. Crosslinking Study Group (2017). U.S. Multicenter Clinical Trial of Corneal Collagen Crosslinking for Treatment of Corneal Ectasia after Refractive Surgery. Ophthalmology, 124(10), 1475–1484. https://doi.org/10.1016/j.ophtha.2017.05.036
55	Hogan, R. B., Johnston, J. H., Long, B. W., Sones, J. Q., Hinton, L. A., Bunge, J., & Corrigan, S. A. (1989). A double-blind, randomized, sham-controlled trial of the gastric bubble for obesity. Gastrointestinal endoscopy, 35(5), 381–385. https://doi.org/10.1016/s0016-5107(89)72839-x

56	Hollenbach, M., Prettin, C., Gundling, F., Schepp, W., Seufert, J., Stein, J., Rösch, T., Aberle, J., Feisthammel, J., Petroff, D., & Hoffmeister, A. (2018). Design of the Weight-loss Endoscopy Trial (WET): a multi-center, randomized, controlled trial comparing weight loss in endoscopically implanted duodenal-jejunal bypass liners vs. intragastric balloons vs. a sham procedure. BMC gastroenterology, 18(1), 118. https://doi.org/10.1186/s12876-018-0838-3
57	Holmlund, T., Levring-Jäghagen, E., Franklin, K. A., Lindkvist, M., & Berggren, D. (2014). Effects of Radiofrequency versus sham surgery of the soft palate on daytime sleepiness. The Laryngoscope, 124(10), 2422–2426. https://doi.org/10.1002/lary.24580
58	Hunter, J. G., Kahrilas, P. J., Bell, R. C., Wilson, E. B., Trad, K. S., Dolan, J. P., Perry, K. A., Oelschlager, B. K., Soper, N. J., Snyder, B. E., Burch, M. A., Melvin, W. S., Reavis, K. M., Turgeon, D. G., Hungness, E. S., & Diggs, B. S. (2015). Efficacy of transoral fundoplication vs omeprazole for treatment of regurgitation in a randomized controlled trial. Gastroenterology, 148(2), 324–333.e5. https://doi.org/10.1053/j.gastro.2014.10.009
59	Ikramuddin, S., Blackstone, R. P., Brancatisano, A., Toouli, J., Shah, S. N., Wolfe, B. M., Fujioka, K., Maher, J. W., Swain, J., Que, F. G., Morton, J. M., Leslie, D. B., Brancatisano, R., Kow, L., O'Rourke, R. W., Deveney, C., Takata, M., Miller, C. J., Knudson, M. B., Tweden, K. S., … Billington, C. J. (2014). Effect of reversible intermittent intra-abdominal vagal nerve blockade on morbid obesity: the ReCharge randomized clinical trial. JAMA, 312(9), 915–922. https://doi.org/10.1001/jama.2014.10540
60	Jarrell, J., Mohindra, R., Ross, S., Taenzer, P., & Brant, R. (2005). Laparoscopy and reported pain among patients with endometriosis. Journal of obstetrics and gynaecology Canada : JOGC = Journal d'obstetrique et gynecologie du Canada : JOGC, 27(5), 477–485. https://doi.org/10.1016/s1701-2163(16)30531-x
61	Kallmes, D. F., Comstock, B. A., Heagerty, P. J., Turner, J. A., Wilson, D. J., Diamond, T. H., Edwards, R., Gray, L. A., Stout, L., Owen, S., Hollingworth, W., Ghdoke, B., Annesley-Williams, D. J., Ralston, S. H., & Jarvik, J. G. (2009). A randomized trial of vertebroplasty for osteoporotic spinal fractures. The New England journal of medicine, 361(6), 569–579. https://doi.org/10.1056/NEJMoa0900563
62	Kalunian, K. C., Moreland, L. W., Klashman, D. J., Brion, P. H., Concoff, A. L., Myers, S., Singh, R., Ike, R. W., Seeger, L. L., Rich, E., & Skovron, M. L. (2000). Visually-guided irrigation in patients with early knee osteoarthritis: a multicenter randomized, controlled trial. Osteoarthritis and cartilage, 8(6), 412–418. https://doi.org/10.1053/joca.1999.0316
63	Kaminski, R., Kulinski, K., Kozar-Kaminska, K., Wasko, M. K., Langner, M., & Pomianowski, S. (2019). Repair Augmentation of Unstable, Complete Vertical Meniscal Tears With Bone Marrow Venting Procedure: A Prospective, Randomized, Double-Blind, Parallel-Group, Placebo-Controlled Study. Arthroscopy: the journal of arthroscopic & related surgery: official publication of the Arthroscopy Association of North America and the International Arthroscopy Association, 35(5), 1500–1508.e1. https://doi.org/10.1016/j.arthro.2018.11.056
64	Kang, J., Lee, G., Kim, J., Kim, Y., Park, S., & Lee, D. (2020). Effects and safety of intranasal phototherapy for allergic rhinitis: Study protocol for a single-center, randomized, double-blind, parallel, placebo-controlled, investigator-initiated, pilot study. Medicine, 99(30), e20835. https://doi.org/10.1097/MD.0000000000020835
65	Kang, T., Sung, C. M., & Yang, H. C. (2019). Radiofrequency ablation of turbinates after septoplasty has no effect on allergic rhinitis symptoms other than nasal obstruction. International forum of allergy & rhinology, 9(11), 1257–1262. https://doi.org/10.1002/alr.22420
66	Kern, R. C., Stolovitzky, J. P., Silvers, S. L., Singh, A., Lee, J. T., Yen, D. M., Iloreta, A. M. C., Jr, Langford, F. P. J., Karanfilov, B., Matheny, K. E., Stambaugh, J. W., Gawlicka, A. K., & RESOLVE II study investigators (2018). A phase 3 trial of mometasone furoate sinus implants for chronic sinusitis with recurrent nasal polyps. International forum of allergy & rhinology, 8(4), 471–481. https://doi.org/10.1002/alr.22084

67	Kernohan, R. M., Anderson, J. R., McKelvey, S. T., & Kennedy, T. L. (1984). A controlled trial of bipolar electrocoagulation in patients with upper gastrointestinal bleeding. The British journal of surgery, 71(11), 889–891. https://doi.org/10.1002/bjs.1800711128
68	Koutsourelakis, I., Georgoulopoulos, G., Perraki, E., Vagiakis, E., Roussos, C., & Zakynthinos, S. G. (2008). Randomised trial of nasal surgery for fixed nasal obstruction in obstructive sleep apnoea. The European respiratory journal, 31(1), 110–117. https://doi.org/10.1183/09031936.00087607
69	Krejs, G. J., Little, K. H., Westergaard, H., Hamilton, J. K., Spady, D. K., & Polter, D. E. (1987). Laser photocoagulation for the treatment of acute peptic-ulcer bleeding. A randomized controlled clinical trial. The New England journal of medicine, 316(26), 1618–1621. https://doi.org/10.1056/NEJM198706253162602
70	Kroslak, M., & Murrell, G. A. C. (2018). Surgical Treatment of Lateral Epicondylitis: A Prospective, Randomized, Double-Blinded, Placebo-Controlled Clinical Trial. The American journal of sports medicine, 46(5), 1106–1113. https://doi.org/10.1177/0363546517753385
71	Laine L. (1987). Multipolar electrocoagulation in the treatment of active upper gastrointestinal tract hemorrhage. A prospective controlled trial. The New England journal of medicine, 316(26), 1613–1617. https://doi.org/10.1056/NEJM198706253162601
72	Landers, S., Hely, A., Harrison, B., Maister, N., Hely, R., Lane, S. E., Gill, S. D., & Page, R. S. (2017). Protocol for a single-centre, parallel-arm, randomised controlled superiority trial evaluating the effects of transcatheter arterial embolisation of abnormal knee neovasculature on pain, function and quality of life in people with knee osteoarthritis. BMJ open, 7(5), e014266. https://doi.org/10.1136/bmjopen-2016-014266
73	Larrosa, F., Hernandez, L., Morello, A., Ballester, E., Quinto, L., & Montserrat, J. M. (2004). Laser-assisted uvulopalatoplasty for snoring: does it meet the expectations?. The European respiratory journal, 24(1), 66–70. https://doi.org/10.1183/09031936.04.00082903
74	Lee, P. E., Kung, R. C., & Drutz, H. P. (2001). Periurethral autologous fat injection as treatment for female stress urinary incontinence: a randomized double-blind controlled trial. The Journal of urology, 165(1), 153–158. https://doi.org/10.1097/00005392-200101000-00037
75	Leon, M. B., Kornowski, R., Downey, W. E., Weisz, G., Baim, D. S., Bonow, R. O., Hendel, R. C., Cohen, D. J., Gervino, E., Laham, R., Lembo, N. J., Moses, J. W., & Kuntz, R. E. (2005). A blinded, randomized, placebo-controlled trial of percutaneous laser myocardial revascularization to improve angina symptoms in patients with severe coronary disease. Journal of the American College of Cardiology, 46(10), 1812–1819. https://doi.org/10.1016/j.jacc.2005.06.079
76	Lindor, K. D., Hughes, R. W., Jr, Ilstrup, D. M., & Jensen, M. D. (1987). Intragastric balloons in comparison with standard therapy for obesity--a randomized, double-blind trial. Mayo Clinic proceedings, 62(11), 992–996. https://doi.org/10.1016/s0025-6196(12)65069-1
77	MacLeod, I. A., Mills, P. R., MacKenzie, J. F., Joffe, S. N., Russell, R. I., & Carter, D. C. (1983). Neodymium yttrium aluminium garnet laser photocoagulation for major haemorrhage from peptic ulcers and single vessels: a single blind controlled study. British medical journal (Clinical research ed.), 286(6362), 345–348. https://doi.org/10.1136/bmj.286.6362.345
78	Matache, B. A., Berdusco, R., Momoli, F., Lapner, P. L., & Pollock, J. W. (2016). A randomized, double-blind sham-controlled trial on the efficacy of arthroscopic tennis elbow release for the management of chronic lateral epicondylitis. BMC musculoskeletal disorders, 17, 239. https://doi.org/10.1186/s12891-016-1093-9
79	Mathus-Vliegen, E. M., Tytgat, G. N., & Veldhuyzen-Offermans, E. A. (1990). Intragastric balloon in the treatment of super-morbid obesity. Double-blind, sham-controlled, crossover evaluation of 500-milliliter balloon. Gastroenterology, 99(2), 362–369. https://doi.org/10.1016/0016-5085(90)91017-z
80	Maurer, J. T., Sommer, J. U., Hein, G., Hörmann, K., Heiser, C., & Stuck, B. A. (2012). Palatal implants in the treatment of obstructive sleep apnea: a randomised, placebo-controlled single-centre trial. European archives of oto-rhino-laryngology: official journal of the European Federation of Oto-Rhino-Laryngological Societies (EUFOS): affiliated with the German Society for Oto-Rhino-Laryngology - Head and Neck Surgery, 269(7), 1851–1856. https://doi.org/10.1007/s00405-011-1920-4

81	Mehta, V., Poply, K., Husband, M., Anwar, S., & Langford, R. (2018). The Effects of Radiofrequency Neurotomy Using a Strip-Lesioning Device on Patients with Sacroiliac Joint Pain: Results from a Single-Center, Randomized, Sham-Controlled Trial. Pain physician, 21(6), 607–618. (No DOI - https://pubmed.ncbi.nlm.nih.gov/30508988/)
82	Meshkinpour, H., Hsu, D., & Farivar, S. (1988). Effect of gastric bubble as a weight reduction device: a controlled, crossover study. Gastroenterology, 95(3), 589–592. https://doi.org/10.1016/s0016-5085(88)80002-7
83	Montgomery, M., Håkanson, B., Ljungqvist, O., Ahlman, B., & Thorell, A. (2006). Twelve months' follow-up after treatment with the EndoCinch endoscopic technique for gastro-oesophageal reflux disease: a randomized, placebo-controlled study. Scandinavian journal of gastroenterology, 41(12), 1382–1389. https://doi.org/10.1080/00365520600735738
84	Moseley, J. B., O'Malley, K., Petersen, N. J., Menke, T. J., Brody, B. A., Kuykendall, D. H., Hollingsworth, J. C., Ashton, C. M., & Wray, N. P. (2002). A controlled trial of arthroscopic surgery for osteoarthritis of the knee. The New England journal of medicine, 347(2), 81–88. https://doi.org/10.1056/NEJMoa013259
85	Nease, C. J., & Krempl, G. A. (2004). Radiofrequency treatment of turbinate hypertrophy: a randomized, blinded, placebo-controlled clinical trial. Otolaryngology--head and neck surgery: official journal of American Academy of Otolaryngology-Head and Neck Surgery, 130(3), 291–299. https://doi.org/10.1016/j.otohns.2003.11.003
86	Ninane, V., Geltner, C., Bezzi, M., Foccoli, P., Gottlieb, J., Welte, T., Seijo, L., Zulueta, J. J., Munavvar, M., Rosell, A., Lopez, M., Jones, P. W., Coxson, H. O., Springmeyer, S. C., & Gonzalez, X. (2012). Multicentre European study for the treatment of advanced emphysema with bronchial valves. The European respiratory journal, 39(6), 1319–1325. https://doi.org/10.1183/09031936.00019711
87	O'Brien, J. D., Day, S. J., & Burnham, W. R. (1986). Controlled trial of small bipolar probe in bleeding peptic ulcers. Lancet (London, England), 1(8479), 464–467. https://doi.org/10.1016/s0140-6736(86)92928-4
88	Olanow, C. W., Goetz, C. G., Kordower, J. H., Stoessl, A. J., Sossi, V., Brin, M. F., Shannon, K. M., Nauert, G. M., Perl, D. P., Godbold, J., & Freeman, T. B. (2003). A double-blind controlled trial of bilateral fetal nigral transplantation in Parkinson's disease. Annals of neurology, 54(3), 403–414. https://doi.org/10.1002/ana.10720
89	Paavola, M., Malmivaara, A., Taimela, S., Kanto, K., Järvinen, T. L., & FIMPACT Investigators (2017). Finnish Subacromial Impingement Arthroscopy Controlled Trial (FIMPACT): a protocol for a randomised trial comparing arthroscopic subacromial decompression and diagnostic arthroscopy (placebo control), with an exercise therapy control, in the treatment of shoulder impingement syndrome. BMJ open, 7(5), e014087. https://doi.org/10.1136/bmjopen-2016-014087
90	Pauza, K. J., Howell, S., Dreyfuss, P., Peloza, J. H., Dawson, K., & Bogduk, N. (2004). A randomized, placebo-controlled trial of intradiscal electrothermal therapy for the treatment of discogenic low back pain. The spine journal: official journal of the North American Spine Society, 4(1), 27–35. https://doi.org/10.1016/j.spinee.2003.07.001
91	Ponce, J., Woodman, G., Swain, J., Wilson, E., English, W., Ikramuddin, S., Bour, E., Edmundowicz, S., Snyder, B., Soto, F., Sullivan, S., Holcomb, R., Lehmann, J., & REDUCE Pivotal Trial Investigators (2015). The REDUCE pivotal trial: a prospective, randomized controlled pivotal trial of a dual intragastric balloon for the treatment of obesity. Surgery for obesity and related diseases: official journal of the American Society for Bariatric Surgery, 11(4), 874–881. https://doi.org/10.1016/j.soard.2014.12.006
92	Powell, N. B., Zonato, A. I., Weaver, E. M., Li, K., Troell, R., Riley, R. W., & Guilleminault, C. (2001). Radiofrequency treatment of turbinate hypertrophy in subjects using continuous positive airway pressure: a randomized, double-blind, placebo-controlled clinical pilot trial. The Laryngoscope, 111(10), 1783–1790. https://doi.org/10.1097/00005537-200110000-00023

93	Ramhamadany, E. M., Fowler, J., & Baird, I. M. (1989). Effect of the gastric balloon versus sham procedure on weight loss in obese subjects. Gut, 30(8), 1054–1057. https://doi.org/10.1136/gut.30.8.1054
94	Risberg, M. A., Ageberg, E., Nilstad, A., Lund, B., Nordsletten, L., Løken, S., Ludvigsen, T., Kierkegaard, S., Carsen, S., Kostogiannis, I., Crossley, K. M., Glyn-Jones, S., & Kemp, J. L. (2018). Arthroscopic Surgical Procedures Versus Sham Surgery for Patients With Femoroacetabular Impingement and/or Labral Tears: Study Protocol for a Randomized Controlled Trial (HIPARTI) and a Prospective Cohort Study (HARP). The Journal of orthopaedic and sports physical therapy, 48(4), 325–335. https://doi.org/10.2519/jospt.2018.7931
95	Roehrborn, C. G., Gange, S. N., Shore, N. D., Giddens, J. L., Bolton, D. M., Cowan, B. E., Brown, B. T., McVary, K. T., Te, A. E., Gholami, S. S., Rashid, P., Moseley, W. G., Chin, P. T., Dowling, W. T., Freedman, S. J., Incze, P. F., Coffield, K. S., Borges, F. D., & Rukstalis, D. B. (2013). The prostatic urethral lift for the treatment of lower urinary tract symptoms associated with prostate enlargement due to benign prostatic hyperplasia: the L.I.F.T. Study. The Journal of urology, 190(6), 2161–2167. https://doi.org/10.1016/j.juro.2013.05.116
96	Romanov, A., Cherniavskiy, A., Novikova, N., Edemskiy, A., Ponomarev, D., Shabanov, V., Losik, D., Elesin, D., Stenin, I., Mikheenko, I., Zhizhov, R., Kretov, E., Pokushalov, E., Po, S. S., Martynyuk, T. V., & Steinberg, J. S. (2020). Pulmonary Artery Denervation for Patients with Residual Pulmonary Hypertension After Pulmonary Endarterectomy. Journal of the American College of Cardiology, 76(8), 916–926. https://doi.org/10.1016/j.jacc.2020.06.064
97	Roos, E. M., Hare, K. B., Nielsen, S. M., Christensen, R., & Lohmander, L. S. (2018). Better outcome from arthroscopic partial meniscectomy than skin incisions only? A sham-controlled randomised trial in patients aged 35-55 years with knee pain and an MRI-verified meniscal tear. BMJ open, 8(2), e019461. https://doi.org/10.1136/bmjopen-2017-019461
98	Rothstein, R., Filipi, C., Caca, K., Pruitt, R., Mergener, K., Torquati, A., Haber, G., Chen, Y., Chang, K., Wong, D., Deviere, J., Pleskow, D., Lightdale, C., Ades, A., Kozarek, R., Richards, W., & Lembo, A. (2006). Endoscopic full-thickness plication for the treatment of gastroesophageal reflux disease: A randomized, sham-controlled trial. Gastroenterology, 131(3), 704–712. https://doi.org/10.1053/j.gastro.2006.07.004
99	Ryösä, A., Kukkonen, J., Björnsson Hallgren, H. C., Moosmayer, S., Holmgren, T., Ranebo, M., Bøe, B., Äärimaa, V., & ACCURATE study group (2019). Acute Cuff Tear Repair Trial (ACCURATE): protocol for a multicentre, randomised, placebo-controlled trial on the efficacy of arthroscopic rotator cuff repair. BMJ open, 9(5), e025022. https://doi.org/10.1136/bmjopen-2018-025022
100	Salem, M., Rotevatn, S., Stavnes, S., Brekke, M., Vollset, S. E., & Nordrehaug, J. E. (2004). Usefulness and safety of percutaneous myocardial laser revascularization for refractory angina pectoris. The American journal of cardiology, 93(9), 1086–1091. https://doi.org/10.1016/j.amjcard.2004.01.032
101	Sarr, M. G., Billington, C. J., Brancatisano, R., Brancatisano, A., Toouli, J., Kow, L., Nguyen, N. T., Blackstone, R., Maher, J. W., Shikora, S., Reeds, D. N., Eagon, J. C., Wolfe, B. M., O'Rourke, R. W., Fujioka, K., Takata, M., Swain, J. M., Morton, J. M., Ikramuddin, S., Schweitzer, M., … EMPOWER Study Group (2012). The EMPOWER study: randomized, prospective, double-blind, multicenter trial of vagal blockade to induce weight loss in morbid obesity. Obesity surgery, 22(11), 1771–1782. https://doi.org/10.1007/s11695-012-0751-8
102	Schrøder, C. P., Skare, Ø., Reikerås, O., Mowinckel, P., & Brox, J. I. (2017). Sham surgery versus labral repair or biceps tenodesis for type II SLAP lesions of the shoulder: a three-armed randomised clinical trial. British journal of sports medicine, 51(24), 1759–1766. https://doi.org/10.1136/bjsports-2016-097098
103	Schwartz, M. P., Wellink, H., Gooszen, H. G., Conchillo, J. M., Samsom, M., & Smout, A. J. (2007). Endoscopic gastroplication for the treatment of gastro-oesophageal reflux disease: a randomised, sham-controlled trial. Gut, 56(1), 20–28. https://doi.org/10.1136/gut.2006.096842

104	Schweitzer, C., Brezin, A., Cochener, B., Monnet, D., Germain, C., Roseng, S., Sitta, R., Maillard, A., Hayes, N., Denis, P., Pisella, P. J., Benard, A., & FEMCAT study group (2020). Femtosecond laser-assisted versus phacoemulsification cataract surgery (FEMCAT): a multicentre participant-masked randomised superiority and cost-effectiveness trial. Lancet (London, England), 395(10219), 212–224. https://doi.org/10.1016/S0140-6736(19)32481-X
105	Scolapio, J. S., Gostout, C. J., Schroeder, K. W., Mahoney, D. W., & Lindor, K. D. (2001). Dysphagia without endoscopically evident disease: to dilate or not?. The American journal of gastroenterology, 96(2), 327–330. https://doi.org/10.1111/j.1572-0241.2001.03514.x
106	Shaheen, N. J., Sharma, P., Overholt, B. F., Wolfsen, H. C., Sampliner, R. E., Wang, K. K., Galanko, J. A., Bronner, M. P., Goldblum, J. R., Bennett, A. E., Jobe, B. A., Eisen, G. M., Fennerty, M. B., Hunter, J. G., Fleischer, D. E., Sharma, V. K., Hawes, R. H., Hoffman, B. J., Rothstein, R. I., Gordon, S. R., … Lightdale, C. J. (2009). Radiofrequency ablation in Barrett's esophagus with dysplasia. The New England journal of medicine, 360(22), 2277–2288. https://doi.org/10.1056/NEJMoa0808145
107	Sihvonen, R., Paavola, M., Malmivaara, A., Itälä, A., Joukainen, A., Nurmi, H., Kalske, J., Järvinen, T. L., & Finnish Degenerative Meniscal Lesion Study (FIDELITY) Group (2013). Arthroscopic partial meniscectomy versus sham surgery for a degenerative meniscal tear. The New England journal of medicine, 369(26), 2515–2524. https://doi.org/10.1056/NEJMoa1305189
108	Sikand, A., Ehmer, D. R., Jr, Stolovitzky, J. P., McDuffie, C. M., Mehendale, N., & Albritton, F. D., 4th (2019). In-office balloon sinus dilation versus medical therapy for recurrent acute rhinosinusitis: a randomized, placebo-controlled study. International forum of allergy & rhinology, 9(2), 140–148. https://doi.org/10.1002/alr.22248
109	Silverberg, G. D., Mayo, M., Saul, T., Fellmann, J., Carvalho, J., & McGuire, D. (2008). Continuous CSF drainage in AD: results of a double-blind, randomized, placebo-controlled study. Neurology, 71(3), 202–209. https://doi.org/10.1212/01.wnl.0000316197.04157.6f
110	Soylu Özler G. (2014). Silver nitrate cauterization: a treatment option for aphthous stomatitis. Journal of cranio-maxillo-facial surgery : official publication of the European Association for Cranio-Maxillo-Facial Surgery, 42(5), e281–e283. https://doi.org/10.1016/j.jcms.2013.10.006
111	Steward, D. L., Huntley, T. C., Woodson, B. T., & Surdulescu, V. (2008). Palate implants for obstructive sleep apnea: multi-institution, randomized, placebo-controlled study. Otolaryngology--head and neck surgery : official journal of American Academy of Otolaryngology-Head and Neck Surgery, 139(4), 506–510. https://doi.org/10.1016/j.otohns.2008.07.021
112	Stone, G. W., Teirstein, P. S., Rubenstein, R., Schmidt, D., Whitlow, P. L., Kosinski, E. J., Mishkel, G., & Power, J. A. (2002). A prospective, multicenter, randomized trial of percutaneous transmyocardial laser revascularization in patients with nonrecanalizable chronic total occlusions. Journal of the American College of Cardiology, 39(10), 1581–1587. https://doi.org/10.1016/s0735-1097(02)01829-6
113	Stuck, B. A., Sauter, A., Hörmann, K., Verse, T., & Maurer, J. T. (2005). Radiofrequency surgery of the soft palate in the treatment of snoring. A placebo-controlled trial. Sleep, 28(7), 847–850. https://doi.org/10.1093/sleep/28.7.847
114	Sullivan, S., Swain, J., Woodman, G., Edmundowicz, S., Hassanein, T., Shayani, V., Fang, J. C., Noar, M., Eid, G., English, W. J., Tariq, N., Larsen, M., Jonnalagadda, S. S., Riff, D. S., Ponce, J., Early, D., Volckmann, E., Ibele, A. R., Spann, M. D., Krishnan, K., … Pryor, A. (2018). Randomized sham-controlled trial of the 6-month swallowable gas-filled intragastric balloon system for weight loss. Surgery for obesity and related diseases: official journal of the American Society for Bariatric Surgery, 14(12), 1876–1889. https://doi.org/10.1016/j.soard.2018.09.486
115	Sutton, C. J., Ewen, S. P., Whitelaw, N., & Haines, P. (1994). Prospective, randomized, double-blind, controlled trial of laser laparoscopy in the treatment of pelvic pain associated with minimal, mild, and moderate endometriosis. Fertility and sterility, 62(4), 696–700. https://doi.org/10.1016/s0015-0282(16)56990-8

116	Swain, C. P., Bown, S. G., Storey, D. W., Kirkham, J. S., Northfield, T. C., & Salmon, P. R. (1981). Controlled trial of argon laser photocoagulation in bleeding peptic ulcers. Lancet (London, England), 2(8259), 1313–1316. https://doi.org/10.1016/s0140-6736(81)91340-4
117	Swain, C. P., Kirkham, J. S., Salmon, P. R., Bown, S. G., & Northfield, T. C. (1986). Controlled trial of Nd-YAG laser photocoagulation in bleeding peptic ulcers. Lancet (London, England), 1(8490), 1113–1117. https://doi.org/10.1016/s0140-6736(86)91835-0
118	Swank, D. J., Swank-Bordewijk, S. C., Hop, W. C., van Erp, W. F., Janssen, I. M., Bonjer, H. J., & Jeekel, J. (2003). Laparoscopic adhesiolysis in patients with chronic abdominal pain: a blinded randomised controlled multi-centre trial. Lancet (London, England), 361(9365), 1247–1251. https://doi.org/10.1016/s0140-6736(03)12979-0
119	Thompson, C. C., Chand, B., Chen, Y. K., DeMarco, D. C., Miller, L., Schweitzer, M., Rothstein, R. I., Lautz, D. B., Slattery, J., Ryan, M. B., Brethauer, S., Schauer, P., Mitchell, M. C., Starpoli, A., Haber, G. B., Catalano, M. F., Edmundowicz, S., Fagnant, A. M., Kaplan, L. M., & Roslin, M. S. (2013). Endoscopic suturing for transoral outlet reduction increases weight loss after Roux-en-Y gastric bypass surgery. Gastroenterology, 145(1), 129–137.e3. https://doi.org/10.1053/j.gastro.2013.04.002
120	Thomsen, J., Bretlau, P., Tos, M., & Johnsen, N. J. (1981). Ménière's disease: endolymphatic sac decompression compared with sham (placebo) decompression. Annals of the New York Academy of Sciences, 374, 820–830. https://doi.org/10.1111/j.1749-6632.1981.tb30922.x
121	Toouli, J., Roberts-Thomson, I. C., Kellow, J., Dowsett, J., Saccone, G. T., Evans, P., Jeans, P., Cox, M., Anderson, P., Worthley, C., Chan, Y., Shanks, N., & Craig, A. (2000). Manometry based randomised trial of endoscopic sphincterotomy for sphincter of Oddi dysfunction. Gut, 46(1), 98–102. https://doi.org/10.1136/gut.46.1.98
122	Vallon, A. G., Cotton, P. B., Laurence, B. H., Armengol Miro, J. R., & Salord Oses, J. C. (1981). Randomised trial of endoscopic argon laser photocoagulation in bleeding peptic ulcers. Gut, 22(3), 228–233. https://doi.org/10.1136/gut.22.3.228
123	van Schie, C. H., Whalley, A., Vileikyte, L., Wignall, T., Hollis, S., & Boulton, A. J. (2000). Efficacy of injected liquid silicone in the diabetic foot to reduce risk factors for ulceration: a randomized double-blind placebo-controlled trial. Diabetes care, 23(5), 634–638. https://doi.org/10.2337/diacare.23.5.634
124	Verheye, S., Jolicœur, E. M., Behan, M. W., Pettersson, T., Sainsbury, P., Hill, J., Vrolix, M., Agostoni, P., Engstrom, T., Labinaz, M., de Silva, R., Schwartz, M., Meyten, N., Uren, N. G., Doucet, S., Tanguay, J. F., Lindsay, S., Henry, T. D., White, C. J., Edelman, E. R., … Banai, S. (2015). Efficacy of a device to narrow the coronary sinus in refractory angina. The New England journal of medicine, 372(6), 519–527. https://doi.org/10.1056/NEJMoa1402556
125	Viswanathan, A., Vedantam, A., Williams, L. A., Koyyalagunta, D., Abdi, S., Dougherty, P. M., Mendoza, T., Bassett, R. L., Hou, P., & Bruera, E. (2020). Percutaneous Cordotomy for Pain Palliation in Advanced Cancer: A Randomized Clinical Trial Study Protocol. Neurosurgery, 87(2), 394–402. https://doi.org/10.1093/neuros/nyz527
126	Walega, D., McCormick, Z., Manning, D., & Avram, M. (2019). Radiofrequency ablation of genicular nerves prior to total knee replacement has no effect on postoperative pain outcomes: a prospective randomized sham-controlled trial with 6-month follow-up. Regional anesthesia and pain medicine, 44, 646-651. https://doi.org/10.1136/rapm-2018-100094
127	Wang, S., Lu, J., Li, Y. A., Zhou, H., Ni, W. F., Zhang, X. L., Zhu, S. P., Chen, B. B., Xu, H., Wang, X. Y., Xiao, J., Huang, H., Chi, Y. L., & Xu, H. Z. (2016). Autologous Olfactory Lamina Propria Transplantation for Chronic Spinal Cord Injury: Three-Year Follow-Up Outcomes from a Prospective Double-Blinded Clinical Trial. Cell transplantation, 25(1), 141–157. https://doi.org/10.3727/096368915X688065

128	Wei, J. T., Nygaard, I., Richter, H. E., Nager, C. W., Barber, M. D., Kenton, K., Amundsen, C. L., Schaffer, J., Meikle, S. F., Spino, C., & Pelvic Floor Disorders Network (2012). A midurethral sling to reduce incontinence after vaginal prolapse repair. The New England journal of medicine, 366(25), 2358–2367. https://doi.org/10.1056/NEJMoa1111967
129	Wood, D. E., Nader, D. A., Springmeyer, S. C., Elstad, M. R., Coxson, H. O., Chan, A., Rai, N. S., Mularski, R. A., Cooper, C. B., Wise, R. A., Jones, P. W., Mehta, A. C., Gonzalez, X., Sterman, D. H., & IBV Valve Trial Research Team (2014). The IBV Valve trial: a multicenter, randomized, double-blind trial of endobronchial therapy for severe emphysema. Journal of bronchology & interventional pulmonology, 21(4), 288–297. https://doi.org/10.1097/LBR.0000000000000110
130	Wu, P. I., Szczesniak, M. M., Maclean, J., Graham, P. H., Quon, H., Choo, L., & Cook, I. J. (2019). Endoscopic dilatation improves long-term dysphagia following head and neck cancer therapies: a randomized control trial. Diseases of the esophagus: official journal of the International Society for Diseases of the Esophagus, 32(6), doy087. https://doi.org/10.1093/dote/doy087
131	Zamboni, P., Tesio, L., Galimberti, S., Massacesi, L., Salvi, F., D'Alessandro, R., Cenni, P., Galeotti, R., Papini, D., D'Amico, R., Simi, S., Valsecchi, M. G., Filippini, G., & Brave Dreams Research Group (2018). Efficacy and Safety of Extracranial Vein Angioplasty in Multiple Sclerosis: A Randomized Clinical Trial. JAMA neurology, 75(1), 35–43. https://doi.org/10.1001/jamaneurol.2017.3825
