# Supplementary material for: How do pilot and feasibility studies inform randomised placebo-controlled trials in surgery? A systematic review
Source: BMJ Open. 2023 Nov 20;13(11):e071094. doi: 10.1136/bmjopen-2022-071094 (PMC10660967; doi:10.1136/bmjopen-2022-071094)
Supplement: Author's manuscript [file bmjopen-2022-071094.draft_revisions.pdf]

BMJ Open

BMJ Open is committed to open peer review. As part of this commitment we make the peer review history of every article we publish publicly available.

When an article is published we post the peer reviewers' comments and the authors' responses online. We also post the versions of the paper that were used during peer review. These are the versions that the peer review comments apply to.

The versions of the paper that follow are the versions that were submitted during the peer review process. They are not the versions of record or the final published versions. They should not be cited or distributed as the published version of this manuscript.

BMJ Open is an open access journal and the full, final, typeset and author-corrected version of record of the manuscript is available on our site with no access controls, subscription charges or pay-per-view fees (http://bmjopen.bmj.com).

If you have any questions on BMJ Open's open peer review process please email info.bmjopen@bmj.com

How do pilot and feasibility studies inform randomised placebo-controlled trials in surgery? A systematic review

Journal:	*BMJ Open*
Manuscript ID	bmjopen-2022-071094
Article Type:	Original research
Date Submitted by the Author:	16-Dec-2022

Complete List of Authors:	Cousins, Sian; University of Bristol, Population Health Sciences<br>Gormley, Alexander; University of Bristol, Dental School<br>Chalmers, Katy; University of Bristol<br>Campbell, Marion; University of Aberdeen, Health Services Research Unit<br>Beard, David; University of Oxford, Nuffield Dept of Orthopaedics, Rheumatology and Musculoskeletal Sciences<br>Blencowe, Natalie; University of Bristol, Bristol Centre for Surgical Research<br>Blazeby, Jane; University of Bristol

Keywords:	SURGERY, Orthopaedic & trauma surgery < SURGERY, Clinical trials < THERAPEUTICS

SCHOLARONE™ Manuscripts

I, the Submitting Author has the right to grant and does grant on behalf of all authors of the Work (as defined in the below author licence), an exclusive licence and/or a non-exclusive licence for contributions from authors who are: i) UK Crown employees; ii) where BMJ has agreed a CC-BY licence shall apply, and/or iii) in accordance with the terms applicable for US Federal Government officers or employees acting as part of their official duties; on a worldwide, perpetual, irrevocable, royalty-free basis to BMJ Publishing Group Ltd ("BMJ") its licensees and where the relevant Journal is co-owned by BMJ to the co-owners of the Journal, to publish the Work in this journal and any other BMJ products and to exploit all rights, as set out in our licence.

The Submitting Author accepts and understands that any supply made under these terms is made by BMJ to the Submitting Author unless you are acting as an employee on behalf of your employer or a postgraduate student of an affiliated institution which is paying any applicable article publishing charge ("APC") for Open Access articles. Where the Submitting Author wishes to make the Work available on an Open Access basis (and intends to pay the relevant APC), the terms of reuse of such Open Access shall be governed by a Creative Commons licence – details of these licences and which Creative Commons licence will apply to this Work are set out in our licence referred to above.

Other than as permitted in any relevant BMJ Author's Self Archiving Policies, I confirm this Work has not been accepted for publication elsewhere, is not being considered for publication elsewhere and does not duplicate material already published. I confirm all authors consent to publication of this Work and authorise the granting of this licence.

For peer review only

How do pilot and feasibility studies inform randomised placebo-controlled trials in surgery? A systematic review

S. Cousins[1], A. Gormley[1], K. Chalmers[1], M. K. Campbell[2], D. J. Beard[3,4,5], N. S Blencowe[1], J. M. Blazeby[1]

[1]Surgical innovation theme, Bristol National Institute for Health and Care Research (NIHR) Biomedical Research Centre, Centre for Surgical Research, Population Health Sciences, Bristol Medical School, Canynge Hall, Whatley Road, Bristol. BS8 2PS

[2]Health Services Research Unit, University of Aberdeen, Aberdeen, UK

[3]Nuffield Department of Orthopaedics, Rheumatology and Musculoskeletal Sciences, NIHR Biomedical Research Centre, University of Oxford, Oxford, UK

[4]NIHR Biomedical Research Centre, Oxford University Hospitals NHS Foundation Trust, Oxford, UK

[5]Royal College of Surgeons (England) Surgical Interventional Trials Unit (SITU), Botnar Research Centre, University of Oxford, Headington, Oxford, UK

Corresponding author - Sian.cousins@bristol.ac.uk

Sources of funding: National Institute for Health and Care Research Biomedical Research Centre at University Hospitals Bristol and Weston NHS Foundation Trust and the University of Bristol. The views expressed are those of the author(s) and not necessarily those of the NIHR or the Department of Health and Social Care. The study funders had no role in the design of the study, data collection, data analysis, interpretation of data, writing of the report or the decision to submit the article for publication.

Submission category – Review

Keywords – pilot, feasibility, surgery, invasive procedures, placebo, complex intervention

For peer review only - http://bmjopen.bmj.com/site/about/guidelines.xhtml

ABSTRACT

Introduction

Randomised controlled trials with a placebo comparator are considered the gold standard study design when evaluating healthcare interventions. These are challenging to design and deliver in surgery. Guidance recommends pilot and feasibility work to optimise main trial design and conduct, however, the extent to which this occurs in surgery is unknown.

Method

A systematic review identified randomised placebo-controlled surgical trials. Articles published from database inception to 31st December 2020 were retrieved from Ovid-MEDLINE, Ovid-EMBASE and CENTRAL electronic databases, hand-searching and expert knowledge. Pilot/feasibility work conducted prior to the RCTs was then identified from examining citations and reference lists. Where studies explicitly stated their intent to inform the design and/or conduct of the future main placebo-controlled surgical trial, they were included. Publication type, clinical area, treatment intervention, number of centres, sample size, comparators, aims, and text about the invasive placebo intervention were extracted.

Results

From 132 placebo surgical RCTs included in the systematic review, 47 potentially eligible pilot/feasibility studies were identified. Of these, four were included as true pilot/feasibility work. Three were original articles, one a conference abstract; three were conducted in orthopaedic surgery and one in oral and maxillofacial surgery. All four included pilot RCTs, with an invasive surgical placebo intervention, randomising 9 to 49 participants in 1 or 2 centres. They explored the acceptability of recruitment and the invasive placebo intervention to patients and trial personnel, and whether blinding was possible. One study examined the characteristics of the proposed invasive placebo intervention using in-depth interviews.

Conclusion

For peer review only - http://bmjopen.bmj.com/site/about/guidelines.xhtml

Published studies reporting feasibility/pilot work undertaken to inform main placebo surgical trials are scarce. In view of the difficulties of undertaking placebo surgical trials, it is recommended that pilot/feasibility studies are conducted, and more are reported to share key findings and optimise the design of main RCTs.

PROSPERO REGISTRATION NUMBER – CRD42021287371

STRENGTHS AND LIMITATIONS OF THIS STUDY

- To our knowledge, this is the first review to systematically identify and examine feasibility and pilot work to inform main surgical placebo RCTs
- It provides an updated systematic review of main surgical placebo RCTs.
- Most studies performed before main trials, which self-identified as ‘feasibility’ or ‘pilot studies’, did not explicitly state intentions or outline aims relevant to informing main placebo surgical RCTs.
- Exemplar feasibility/pilot studies were identified that examined the acceptability, design and ability of invasive placebo comparators to blind patients and trial persons.
- The current review is limited to the published literature and it is possible that feasibility and pilot work was conducted but not reported

For peer review only - http://bmjopen.bmj.com/site/about/guidelines.xhtml

INTRODUCTION

Randomised controlled trials (RCTs) with a placebo comparator have the potential to answer key clinical questions regarding the effectiveness of invasive procedures, including surgery. Indeed, exemplar studies have been undertaken that have informed clinical practice.(1, 2) Placebo-controlled trials in surgery and other invasive procedures are, however, challenging. There are ethical issues (3, 4) related to potential risk to patients, and practical issues regarding the acceptability of the design of the invasive placebo comparator to patients and trial personnel.

Current guidance for the development of complex healthcare interventions (5) and for the design of placebo surgical trials (6) supports feasibility and pilot work to optimise evaluation in a main RCT. Pilot and feasibility work can address uncertainties related to the integrity of the study protocol, recruitment and retention, outcome measures, randomisation procedures, as well as development and acceptability of the intervention itself.(7, 8) This is particularly important for placebo surgical trials where acceptability and feasibility may be more challenging. It is unknown to what extent such preparatory work is conducted prior to main surgical placebo trials.

The aim of this review is to examine the extent and type of feasibility and pilot work conducted prior to main placebo-controlled trials of surgery, and identify exemplar studies to inform future work.

METHODS

Feasibility/pilot work conducted in preparation for main randomised placebo-controlled surgical trials was identified through examination of RCTs retrieved by a systematic review (PROSPERO registration CRD42021287371).

Systematic review of placebo-controlled randomised surgical trials

An existing review (9) which identified placebo surgical trials was updated by extending the searches to 31st December 2020. Searches used the same search terms and electronic databases (Ovid

For peer review only - http://bmjopen.bmj.com/site/about/guidelines.xhtml

MEDLINE, Ovid EMBASE, and CENTRAL).(10) Additional articles, with no restriction on publication date were identified by hand searching and expert knowledge.

Eligibility criteria – Placebo-controlled randomised surgical trials

Eligibility criteria are described fully in Cousins et al. 2019.(9) Briefly, articles reporting randomised trials (including follow-ups, protocols and any self-reported as 'pilot' RCTs) comparing surgery with placebo interventions were included. Surgery was defined as any invasive procedure that changes the anatomy and requires a skin incision or use of endoscopic techniques.(11) Placebo interventions referred to any surgical procedure that was intended to mimic the treatment intervention under evaluation. Pharmaceutical or dental interventions and reviews were excluded. Protocols of all included studies were retrieved, where available.

Identifying published pilot/feasibility studies conducted to inform placebo-controlled randomised surgical trials

Main trial publications identified by the systematic review were read fully, including protocols and clinical trials registry entries, where available. Potentially eligible feasibility/pilot studies were identified in two ways. In the first instance, studies referred to explicitly in the main trial text using the terms 'feasibility' or 'pilot', with associated reference(s) were retrieved. Where references were not provided, publication lists of the corresponding author were hand-searched to identify any publications related to work undertaken before the main trial that were cited as being relevant to the feasibility/pilot work mentioned. Secondly, reference lists of the main trials were hand-searched to identify studies self-reported in the title as 'feasibility' or 'pilot' studies. The use of these terms to identify feasibility/pilot work is supported by a study conducted by Eldridge et al 2018 that aimed to develop a conceptual framework for defining feasibility and pilot studies. The study found that both terms are usually included in the titles and abstracts of studies undertaken in preparation for effectiveness RCTs.(7) Main trial texts were examined by two reviewers (SC and AG) independently. RCTs included in the systematic review update that were self-reported as 'pilot' were also examined

For peer review only - http://bmjopen.bmj.com/site/about/guidelines.xhtml

for eligibility. Scoping searches on Medline (Ovid SP) electronic database using the search concepts 'feasibility/pilot', 'surgery' and 'placebo', combined with 'and' but with no additional filters, did not retrieve any relevant results. Therefore, additional systematic searches of electronic databases for pilot and feasibility studies were not conducted.

Eligibility criteria – Feasibility and pilot studies to inform main RCTs

For the purposes of this review, eligible studies were those that included an explicit statement that the work was to inform the design and/or conduct of a future main placebo-controlled surgical trial (irrespective of whether the study was labelled or entitled pilot/feasibility or if it had only been referred to as pilot/feasibility work in the main trial text). Studies of any design, with aims including, but not limited to, the acceptability of interventions to patients and clinicians, recruitment and retention, and development of invasive placebo interventions, were included. Internal pilot studies (those which solely tested the finalised design as part of the main trial) and those primarily assessing efficacy/effectiveness/safety outcomes were excluded. Original articles and conference abstracts were included; letters, editorials, and reviews were excluded. Two reviewers (SC, AG, KC) screened identified studies independently. Disagreements were resolved by consensus or by a 3rd senior reviewer (JMB), if necessary. Where included feasibility/pilot publications referenced additional related publications, these were retrieved to inform data extraction.

Data extraction and analysis

Data extracted from included RCTs identified by the systematic review update were: year of publication, region, clinical area (e.g., gastrointestinal), number of centres, number of patients randomised, and treatment intervention. Data extracted from included feasibility/pilot studies were: publication type (original article or abstract), clinical area and treatment intervention, study design, number of centres, sample size, comparison groups, reported study aims, and any text related to the invasive placebo intervention, specifically regarding work done to inform its development and whether studies reported criteria against which decisions would be made to progress to a main RCT.

Data were extracted by one reviewer using a standardised data extraction form. A second reviewer extracted data for 20% of articles to identify any potential systematic errors in data extraction. Where multiple articles related to the same study, they were grouped into a single set, and data extraction was conducted on a "per trial" (rather than "per article") basis. Descriptive statistics summarised basic data where appropriate and verbatim text was summarised descriptively.

Patient and public involvement

No patient involved

RESULTS

Placebo-controlled randomised trials of invasive procedures

The 96 articles identified by the previous review(9) were added to those identified in the current review update. Searches retrieved 1918 articles, of which 113 full texts were screened and 36 trials included (Figure 1). Finally included were 132 trials; this included four RCTs that were self-reported as 'pilot' identified by the systematic review update. Characteristics are shown in Table 1.

For peer review only

For peer review only - http://bmjopen.bmj.com/site/about/guidelines.xhtml

Table 1. Characteristics of placebo-controlled randomised controlled trials of invasive procedures identified in the systematic review update

Characteristic	Number of RCTs, n=132 (%)
Year of publication	
≤2000	29 (22)
2001-2010	39 (30)
2011-2020	64 (48)
Region	
United States	44 (33)
Mainland Europe	32 (24)
United Kingdom	17 (13)
Australia	10 (8)
Asia	7 (5)
Canada	3 (2)
South America	1 (1)
Multi-region	18 (14)
Clinical area	
Gastrointestinal	44 (33)
Orthopaedics and trauma	29 (22)
Oral and maxillofacial	22 (17)
Interventional cardiology	10 (8)
Cardiothoracic	7 (5)
Neurosurgery	6 (4)
Gynaecology and obstetrics	5 (4)
Ophthalmology	4 (3)
Podiatry	3 (3)
Urology	2 (2)
Number of centres	
1	44 (33)
2-5	25 (19)
6-10	9 (7)
>10	22 (17)
Not reported	32 (24)
Number of patients randomised[a]	
1-100	83 (63)
101-200	23 (17)
>200	20 (15)
Treatment intervention	
Endoscopic	50 (38)
Minimal access	38 (29)
Percutaneous	31 (23)
Open surgery	13 (10)

[a]n=6 included protocols (number of patients randomised not reported)

Feasibility and pilot studies conducted to inform main placebo-controlled surgical RCTs

Of the 128 main placebo RCTs identified (Figure 2), 29 referenced studies explicitly referred to as ‘feasibility’ or ‘pilot’ studies in the main trial text. Hand-searching references of the papers found a further 14 studies self-reported as ‘feasibility’ or ’pilot’ studies in the title. These were combined with the four RCTs self-reported as ‘pilot’ identified in the systematic review (total 47 papers examined).

For peer review only - http://bmjopen.bmj.com/site/about/guidelines.xhtml

For peer review only

202 In-depth reading of these found that the majority (n=37) did not report pilot or feasibility work to inform a placebo-controlled main RCT and so were excluded. Although they had been referred to, or were self-reported as, pilot or feasibility studies, there was no explicit statement in the whole report that the work was intended to inform a subsequent main placebo-controlled surgical trial. All the papers wrote about intentions to examine treatment effects. Two papers reporting 'internal pilot' studies, two duplicates (15, 16) (papers that were identified twice, and are included below) and one review (17) and letter (18) were also excluded.

209 Four studies were explicit in their intention to inform main placebo surgical RCTs design and conduct and were included (15, 16, 19, 20). All self-reported as a pilot or feasibility study in the title and outlined aims related to their intention to inform a future main placebo-controlled surgical RCT in their introduction. Study details are summarised in Table 2 and described individually in detail below.

For peer review only

Table 2. Study details of feasibility and pilot studies identified to inform main placebo surgical RCTs

Author (Year)	Publication type	Clinical area (Treatment intervention)	Study design (Comparator group)	# centers	Sample size	Reported study aims
Campbell (2011)	Original article	Orthopaedics (Arthroscopic lavage for knee osteoarthritis)	Qualitative interviews and focus groups	NA	257	Examine the need for a placebo-controlled main RCT; whether an appropriate placebo can be designed (including surgical and anesthetic components); and the acceptability of an invasive placebo to patients, clinicians and ethics committee chairs
			Survey	NA	780	Examine acceptability of an invasive placebo to health professionals
			Pilot RCT (Invasive placebo and no treatment)	2	49	Feasibility of recruitment
Kallmes (2002)	Conference abstract	Orthopaedics (Percutaneous vertebroplasty for osteoporotic spinal fractures)	Pilot RCT (Invasive placebo)	NR	5	Feasibility of recruitment
Powell (2001)	Original article	Oral and maxillofacial (Radiofrequency reduction of turbinate hypertrophy for sleep-disordered breathing)	Pilot RCT (Invasive placebo)	1	22	Estimate treatment effect and impact of study design<br>Determine appropriate study design of main trial<br>Feasibility of conducting main RCT
Moseley (1996)	Original article	Orthopaedics (Arthroscopic debridement for knee osteoarthritis)	Pilot RCT (Invasive placebo)	1	10	Examine the need for a placebo-controlled main RCT<br>Feasibility of recruitment<br>Develop and test outcome measures<br>Acceptability of placebo<br>Ability of placebo to blind patients

NA, Not applicable; RCT, Randomised controlled trial; NR, Not reported

For peer review only

For peer review only - http://bmjopen.bmj.com/site/about/guidelines.xhtml

1. *Campbell et al. 2011* (20, 21)

This mixed methods study, published as an original article, examined the feasibility of a main trial evaluating arthroscopic lavage compared with placebo surgery and non-operative management for patients with knee osteoarthritis. An initial exploratory phase consisted of in-depth qualitative (interviews and focus groups) (n=257) and quantitative (postal surveys) work (n=780) to explore views and opinions about a main trial. Issues of the design of the invasive placebo comparator, including surgical and anaesthetic components, and the acceptability of this to key stakeholders, including patients, health professionals and chairs of ethics committees were explored. The final phase was a pilot RCT. This randomised 49 patients from two centres to examine the feasibility of the proposed placebo-controlled design, including gaining ethical and local approvals and recruitment of patients and delivering placebo surgery. Decisions about whether to progress to a main RCT were reported. The authors concluded that a main placebo surgical trial could be successfully designed and was generally acceptable, however the considerable barriers faced in conducting the trial in practice (mainly concerning barriers to recruitment of centres, especially securing management permissions at sites) meant a main RCT was not feasible and did not take place.

2. *Kallmes et al 2002 (19)*

This study, published as a conference abstract, examined whether recruitment to a trial comparing vertebroplasty or a placebo for osteoporotic spinal fractures was possible. This was assessed within a pilot RCT that randomised five patients in one centre to treatment or placebo interventions. Although not specified as an explicit aim, the study reported blinding success by asking participants to guess which procedure they had received. The authors concluded that enrolment of patients into a main placebo-controlled trial was feasible.(22)

3. *Powell et al 2001* (15)

This single centre study aimed to assess the impact of inclusion of an invasive placebo comparator on estimates of treatment effect. A pilot RCT randomised 22 participants with sleep disordered breathing

For peer review only - http://bmjopen.bmj.com/site/about/guidelines.xhtml

For peer review only

to temperature-controlled radiofrequency reduction of turbinate hypertrophy or an invasive placebo (with no radiofrequency applied). The study compared outcomes between blinded and unblinded assessors. It found that unblinded assessment yielded greater treatment effect (bias) and this was used as rationale by the authors for the need to include a placebo comparator in a main trial. Although not explicitly outlined as a study aim authors commented that it was feasible to design a placebo procedure with only the 'active ingredient' withheld. The authors concluded that a future definitive study was feasible and inclusion of a placebo was critical, however it is unclear whether it took place as no main trial publication could be found.

4. *Moseley et al 1996* (16)

This study, published as an original article, aimed to determine whether a placebo control was necessary in the main RCT, the feasibility of recruitment, the ability of the placebo to blind participants and outcome assessors, and the satisfaction of patients allocated to placebo. They conducted a pilot RCT of 10 patients in one centre. The study interventions included arthroscopic debridement of the knee for osteoarthritis, arthroscopic lavage or an invasive placebo procedure. Authors did not comment specifically on the development of the invasive placebo intervention. Patient satisfaction was assessed by asking the questions 'would you recommend the surgery to your friends and family?' and 'do you think the operation was worthwhile?'. The success of blinding participants and personnel was examined by asking participants and physicians to guess which procedure was performed at all post-operative visits. Patients and outcome assessors were unable to consistently guess which procedure had been delivered. The authors commented that getting study approvals from the necessary committees and institutions was a slow process, with approvals gained only after it was made clear that participants would be fully informed about the placebo-controlled nature of the study. The authors concluded that the main RCT should include an invasive placebo procedure and that recruitment was feasible, as was the ability of the placebo to blind patients. A main trial was completed, randomising 180 patients.(1)

For peer review only - http://bmjopen.bmj.com/site/about/guidelines.xhtml

For peer review only

DISCUSSION

Placebo controlled trials in surgery are challenging to design and deliver and can be contentious. Guidance recommends that pre-trial pilot and feasibility work be undertaken to optimise the design and conduct of the trial. Placebo-controlled surgical RCTs identified by the systematic review were rigorously and systematically examined to identify feasibility or pilot work conducted and published to inform the main trial design and conduct. Of the 132 RCTs identified, 47 referred to or referenced 'feasibility' or 'pilot' studies. On detailed scrutiny the vast majority did not state any intention to inform a main placebo-controlled randomised trial, assessing instead the effectiveness/efficacy of the treatment intervention. Four feasibility/pilot studies did outline aims to inform methodological aspects of the main RCT. These focussed on key uncertainties of recruitment, the need for a placebo comparator in the main RCT, the ability of the invasive placebo to blind participants and trial persons, the acceptability of an invasive placebo comparator to patients and clinicians, and in one study, the potential components/design of the invasive placebo. All four of the feasibility/pilot studies included a pilot RCT. One (20, 21) also employed interviews and focus groups, and a postal survey to examine the design and acceptability of an invasive placebo. The identified studies highlighted the importance of preparatory work and how it can have a major influence on the design of the definitive placebo-surgical trial – both in shaping the final design and informing the feasibility (or otherwise) of progressing to a main trial. The current work found that main placebo-controlled surgical trials are not often informed by published pilot and feasibility studies. It is recommended that feasibility work is conducted and published, not only to optimise the design and conduct of main placebo surgical RCTs, but also to reduce research waste and share lessons learnt.

The limited feasibility and pilot work assessing methodological considerations of placebo surgical RCTs may be due to a historical lack of clarity about the meaning and design of 'pilot' and 'feasibility' studies.(16) Commonly pilot studies have been labelled as such to justify small sample sizes, rather than explore the feasibility of conducting a main RCT. It may also be due to challenges in publishing these studies due to editorial policy; so these studies may have been conducted but not published.(18)

For peer review only - http://bmjopen.bmj.com/site/about/guidelines.xhtml

292 Recent work has made strides in clarifying definitions (26, 27) and highlights feasibility aims appropriate for assessment in feasibility/pilot studies.(6) The publication of guidance for the reporting of pilot and feasibility studies, including the pilot and feasibility extension of the Consolidated Standards of Reporting Trials (27-29), and the emergence of journals specific to this area (30) may facilitate the optimisation of the design, reporting and publication of these studies. Guidance for using qualitative research in feasibility studies for trials has also been published.(31) This consists of a list of 16 items within five domains (research questions, data collection, analysis, teamworking and reporting) that should be considered when assessing or undertaking qualitative research within feasibility studies for RCTs. Qualitative work has the propensity to add rich information about intervention acceptability and feasibility of delivery; issues paramount when developing and piloting invasive placebo interventions.

303 Specific to the design and conduct of surgical placebo-controlled trials, published guidelines (6) recommend pilot work to inform the design of invasive placebo procedures, and provides details about how treatment interventions may be deconstructed to identify critical surgical element/s (that can then be omitted from the placebo).(23) The controversial nature of surgical and invasive interventions means that feasibility and pilot work is of the utmost importance. There are issues around the acceptability of the trial to patients, trial personnel and ethics committees due to perceived potential risk. Designing an invasive placebo is also challenging and feasibility work should include examination of which components of the surgery should be included (and omitted) from the invasive placebo intervention and whether it is able to effectively blind participants and trial personnel to trial group allocation.

313 Inappropriate emphasis on hypothesis testing within pilot and feasibility work, rather than the methodological aspects of the design of a future main RCT is shown in other reviews of published literature. Shanyinde (24) identified studies with 'pilot' or 'feasibility' in the title published between 2000-2009 and found that of the 50 papers sampled 56% examined methodological issues in-depth

For peer review only - http://bmjopen.bmj.com/site/about/guidelines.xhtml

and 18% discussed a future trial. Similarly, Arain et al (14), who examined published studies found with the keywords 'pilot' and 'feasibility' between 2007 and 2008, found that of the 54 studies identified, only 12 were actually undertaken in preparation for a main trial. Arain et al also searched the United Kingdom Clinical Research Network portfolio for feasibility/pilot studies and of the 34 identified, only 12 tested some component of the research process. A review examining feasibility and pilot studies of surgical interventions funded by the United Kingdom National Institute for Health and Care Research programmes from 2005 to 2015 (25) found that although over half of the 35 studies identified examined methodological components, such as recruitment or the current intervention, fewer looked at aspects specific to surgery (n=10). Another review examining published 'pilot' RCTs in orthopaedic surgery found that of the 49 studies included, the majority (n=28) evaluated the efficacy of the intervention.(26)

The current work provides an update to previous systematic reviews of placebo-controlled randomised surgical trials. Main trial documents, including protocols and clinical trials registries, were rigorously examined by two independent reviewers to identify feasibility and pilot work. This review is however, restricted to published feasibility and pilot studies and did not search for pilot and feasibility studies specifically using electronic databases; although scope searches using search concepts 'feasibility/pilot', 'surgery' and 'placebo' did not identify any additional relevant feasibility/pilot studies. It is also possible that studies may have been conducted and not published. The exemplar papers identified may be used to inform future feasibility studies in this area. These studies provide useful details about the design and conduct of placebo surgical RCTs specifically, including potential study aims (e.g., the ability of the proposed invasive placebo to blind participants and trial persons) and the use of qualitative methods.

There is a dearth of feasibility and pilot work conducted and published to inform the design and conduct of placebo surgical RCTs. Given the challenging nature of these studies, including practical and ethical considerations, feasibility and pilot studies are needed. These will ensure main RCTs are

For peer review only - http://bmjopen.bmj.com/site/about/guidelines.xhtml

For peer review only

feasible and that the proposed invasive placebo interventions are acceptable and effective in blinding participants and trial persons.

AUTHOR CONTRIBUTIONS

SC: conceptualisation; methodology; formal analysis; supervision; writing – original draft; AG: formal analysis; writing – review and editing; KC: formal analysis; writing – review and editing; MKC: conceptualisation; methodology; writing – review and editing; DJB: conceptualisation; methodology; writing – review and editing; NSB: conceptualisation; methodology; writing – review and editing; JMB: funding acquisition; conceptualisation; metholodology; formal analysis; supervision; writing – review and editing.

COMPETING INTERESTS

The authors declare no conflicts of interest and the funder of this study had no involvement in study design, data collection, data analysis, or manuscript preparation.

FUNDING

This work was supported by the National Institute for Health and Care Research Biomedical Research Centre at University Hospitals Bristol and Weston NHS Foundation Trust and the University of Bristol (BRC-1215-20011).

DATA SHARING STATEMENT

Data extracted is presented in tables 1 and 2 and citations included for articles included in the review for reference.

ETHICS APPROVAL STATEMENT

The study did not include human participants and ethical approval was not required.

REFERENCES

1. Moseley JB, O'Malley K, Petersen NJ, Menke TJ, Brody BA, Kuykendall DH, et al. A controlled trial of arthroscopic surgery for osteoarthritis of the knee. N Engl J Med. 2002;347(2):81-8.

For peer review only - http://bmjopen.bmj.com/site/about/guidelines.xhtml

2. Beard DJ, Rees JL, Cook JA, Rombach I, Cooper C, Merritt N, et al. Arthroscopic subacromial decompression for subacromial shoulder pain (CSAW): a multicentre, pragmatic, parallel group, placebo-controlled, three-group, randomised surgical trial. Lancet. 2018;391(10118):329-38.

3. Horng S, Miller FG. Ethical framework for the use of sham procedures in clinical trials. Crit Care Med. 2003;31(3 Suppl):S126-30.

4. Savulescu J, Wartolowska K, Carr A. Randomised placebo-controlled trials of surgery: ethical analysis and guidelines. J Med Ethics. 2016;42(12):776-83.

5. Skivington K, Matthews L, Simpson SA, Craig P, Baird J, Blazeby JM, et al. A new framework for developing and evaluating complex interventions: update of Medical Research Council guidance. Bmj. 2021;374:n2061.

6. Beard DJ, Campbell MK, Blazeby JM, Carr AJ, Weijer C, Cuthbertson BH, et al. Considerations and methods for placebo controls in surgical trials (ASPIRE guidelines). Lancet. 2020;395(10226):828-38.

7. Eldridge SM, Lancaster GA, Campbell MJ, Thabane L, Hopewell S, Coleman CL, et al. Defining Feasibility and Pilot Studies in Preparation for Randomised Controlled Trials: Development of a Conceptual Framework. PLoS One. 2016;11(3):e0150205.

8. Lancaster GA, Dodd S, Williamson PR. Design and analysis of pilot studies: recommendations for good practice. J Eval Clin Pract. 2004;10(2):307-12.

9. Cousins S, Blencowe NS, Tsang C, Lorenc A, Chalmers K, Carr AJ, et al. Reporting of key methodological issues in placebo-controlled trials of surgery needs improvement: a systematic review. J Clin Epidemiol. 2020;119:109-16.

10. Goossen K, Tenckhoff S, Probst P, Grummich K, Mihaljevic AL, Buchler MW, et al. Optimal literature search for systematic reviews in surgery. Langenbecks Arch Surg. 2018;403(1):119-29.

11. Wartolowska K, Collins GS, Hopewell S, Judge A, Dean BJ, Rombach I, et al. Feasibility of surgical randomised controlled trials with a placebo arm: a systematic review. BMJ Open. 2016;6(3):e010194.

For peer review only

For peer review only - http://bmjopen.bmj.com/site/about/guidelines.xhtml

392 12. Whitehead AL, Sully BG, Campbell MJ. Pilot and feasibility studies: is there a difference from each other and from a randomised controlled trial? Contemporary clinical trials. 2014;38(1):130-3.

394 13. Thabane L, Ma J, Chu R, Cheng J, Ismaila A, Rios LP, et al. A tutorial on pilot studies: the what, why and how. BMC medical research methodology. 2010;10(1):1.

396 14. Arain M, Campbell MJ, Cooper CL, Lancaster GA. What is a pilot or feasibility study? A review of current practice and editorial policy. BMC medical research methodology. 2010;10:67.

398 15. Powell NB, Zonato AI, Weaver EM, Li K, Troell R, Riley RW, et al. Radiofrequency treatment of turbinate hypertrophy in subjects using continuous positive airway pressure: a randomized, double-blind, placebo-controlled clinical pilot trial. Laryngoscope. 2001;111(10):1783-90.

401 16. Moseley JB, Jr., Wray NP, Kuykendall D, Willis K, Landon G. Arthroscopic treatment of osteoarthritis of the knee: a prospective, randomized, placebo-controlled trial. Results of a pilot study. The American journal of sports medicine. 1996;24(1):28-34.

404 17. Cook IJ. Diagnostic evaluation of dysphagia. Nature clinical practice Gastroenterology & hepatology. 2008;5(7):393-403.

406 18. Malek F, Neuzil P, Gustafsson F, Kaye DM, Walton A, Mates M, et al. Clinical outcome of transcatheter treatment of heart failure with preserved or mildly reduced ejection fraction using a novel implant. International journal of cardiology. 2015;187:227-8.

409 19. Kallmes DF, Jensen ME, Marx WF, Sinha Roy S, Schweickert PA, Jarvik JG. A Pilot Study for a Sham-Controlled, Randomized, Prospective, Crossover Trial of Percutaneous Vertebroplasty. American Society of Neuroradiology; May 13th; Vancouver2002.

412 20. Campbell MK, Entwistle VA, Cuthbertson BH, Skea ZC, Sutherland AG, McDonald AM, et al. Developing a placebo-controlled trial in surgery: issues of design, acceptability and feasibility. Trials. 2011;12:50.

415 21. Campbell MK, Skea ZC, Sutherland AG, Cuthbertson BH, Entwistle VA, McDonald AM, et al. Effectiveness and cost-effectiveness of arthroscopic lavage in the treatment of osteoarthritis of the

For peer review only

For peer review only - http://bmjopen.bmj.com/site/about/guidelines.xhtml

knee: a mixed methods study of the feasibility of conducting a surgical placebo-controlled trial (the KORAL study). Health technology assessment (Winchester, England). 2010;14(5):1-180.

22. Kallmes DF, Comstock BA, Heagerty PJ, Turner JA, Wilson DJ, Diamond TH, et al. A randomized trial of vertebroplasty for osteoporotic spinal fractures. N Engl J Med. 2009;361(6):569-79.

23. Cousins S, Blencowe NS, Tsang C, Chalmers K, Mardanpour A, Carr AJ, et al. Optimizing the design of invasive placebo interventions in randomized controlled trials. Br J Surg. 2020.

24. Shanyinde M, Pickering RM, Weatherall M. Questions asked and answered in pilot and feasibility randomized controlled trials. BMC medical research methodology. 2011;11:117.

25. Fairhurst K, Blazeby JM, Potter S, Gamble C, Rowlands C, Avery KNL. Value of surgical pilot and feasibility study protocols. Br J Surg. 2019;106(8):968-78.

26. Desai B, Desai V, Shah S, Srinath A, Saleh A, Simunovic N, et al. Pilot randomized controlled trials in the orthopaedic surgery literature: a systematic review. BMC Musculoskelet Disord. 2018;19(1):412.

For peer review only

For peer review only - http://bmjopen.bmj.com/site/about/guidelines.xhtml

Figure 1. PRISMA diagram showing screening process of retrieved articles

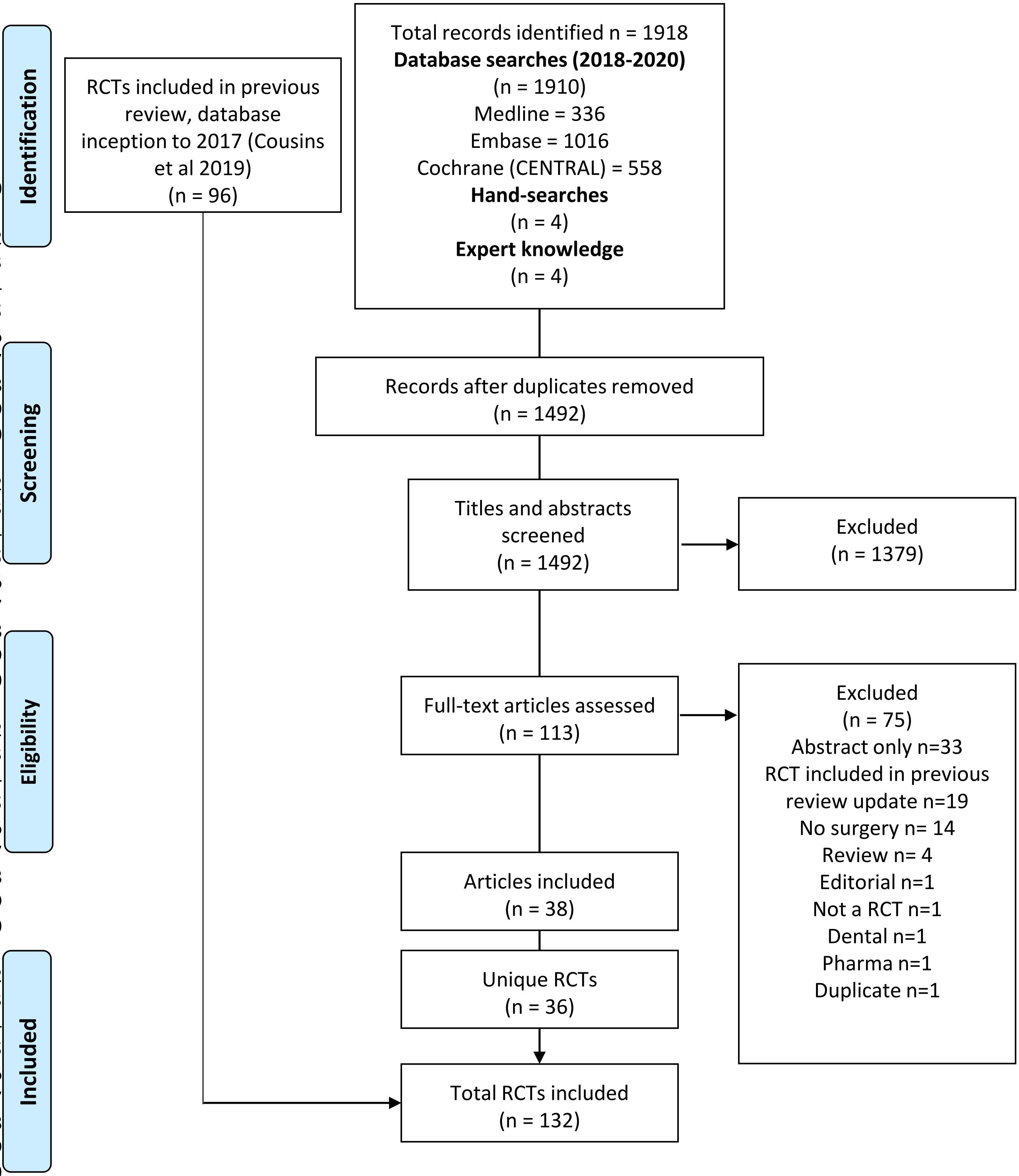

RCTs, Randomised Controlled Trials

Figure 2. PRISMA diagram showing screening process of feasibility and pilot studies

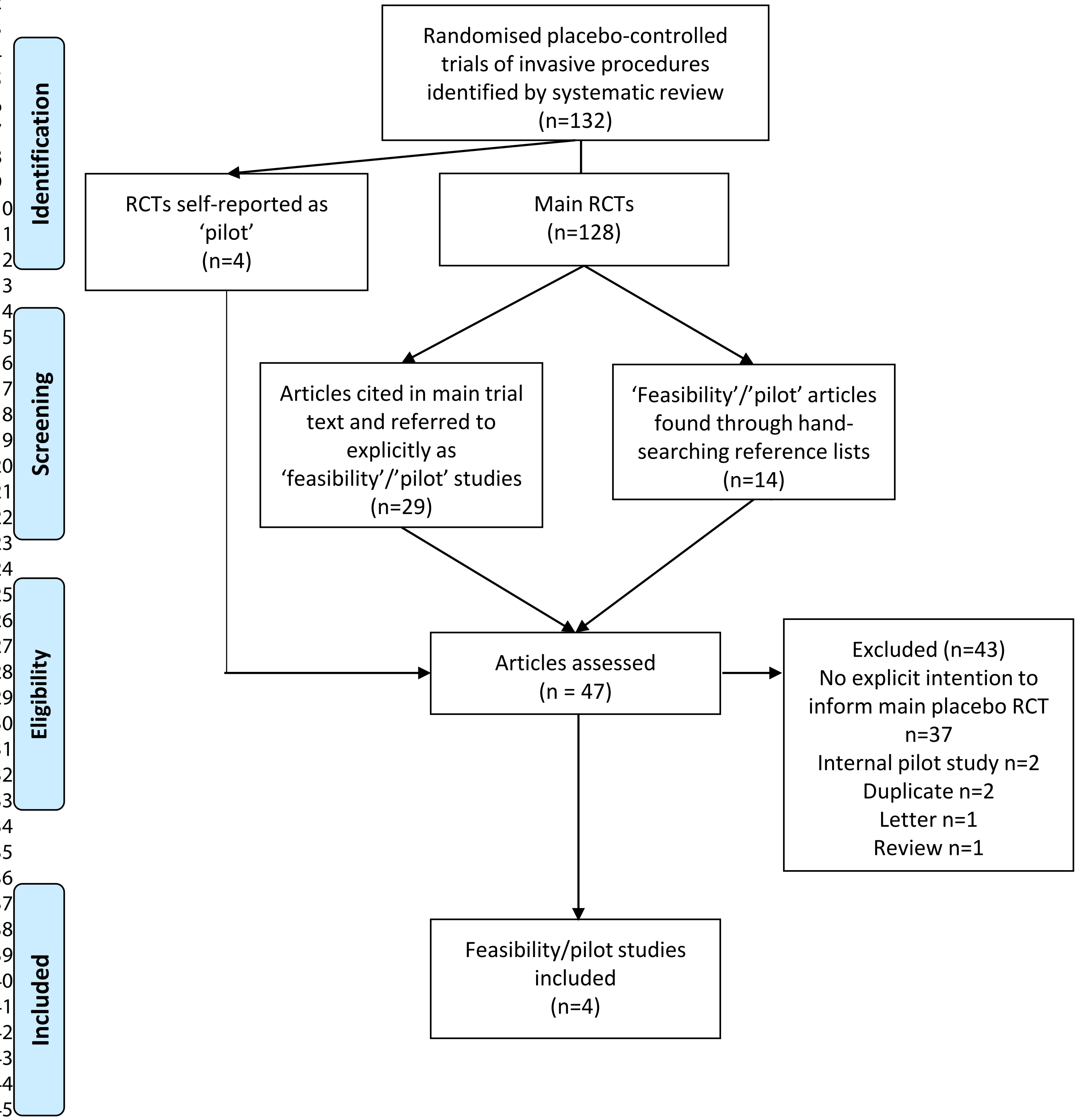

RCTs, Randomised Controlled Trials

PRISMA 2020 Checklist

Section and Topic	Item #	Checklist item	Location where item is reported
**TITLE**			
Title	1	Identify the report as a systematic review.	1
**ABSTRACT**			
Abstract	2	See the PRISMA 2020 for Abstracts checklist.	yes
**INTRODUCTION**			
Rationale	3	Describe the rationale for the review in the context of existing knowledge.	4
Objectives	4	Provide an explicit statement of the objective(s) or question(s) the review addresses.	4
**METHODS**			
Eligibility criteria	5	Specify the inclusion and exclusion criteria for the review and how studies were grouped for the syntheses.	5-6
Information sources	6	Specify all databases, registers, websites, organisations, reference lists and other sources searched or consulted to identify studies. Specify the date when each source was last searched or consulted.	5-6
Search strategy	7	Present the full search strategies for all databases, registers and websites, including any filters and limits used.	5
Selection process	8	Specify the methods used to decide whether a study met the inclusion criteria of the review, including how many reviewers screened each record and each report retrieved, whether they worked independently, and if applicable, details of automation tools used in the process.	5-6
Data collection process	9	Specify the methods used to collect data from reports, including how many reviewers collected data from each report, whether they worked independently, any processes for obtaining or confirming data from study investigators, and if applicable, details of automation tools used in the process.	6-7
Data items	10a	List and define all outcomes for which data were sought. Specify whether all results that were compatible with each outcome domain in each study were sought (e.g. for all measures, time points, analyses), and if not, the methods used to decide which results to collect.	6-7
	10b	List and define all other variables for which data were sought (e.g. participant and intervention characteristics, funding sources). Describe any assumptions made about any missing or unclear information.	6-7
Study risk of bias assessment	11	Specify the methods used to assess risk of bias in the included studies, including details of the tool(s) used, how many reviewers assessed each study and whether they worked independently, and if applicable, details of automation tools used in the process.	na
Effect measures	12	Specify for each outcome the effect measure(s) (e.g. risk ratio, mean difference) used in the synthesis or presentation of results.	na
Synthesis methods	13a	Describe the processes used to decide which studies were eligible for each synthesis (e.g. tabulating the study intervention characteristics and comparing against the planned groups for each synthesis (item #5)).	na
	13b	Describe any methods required to prepare the data for presentation or synthesis, such as handling of missing summary statistics, or data conversions.	na
	13c	Describe any methods used to tabulate or visually display results of individual studies and syntheses.	na
	13d	Describe any methods used to synthesize results and provide a rationale for the choice(s). If meta-analysis was performed, describe the model(s), method(s) to identify the presence and extent of statistical heterogeneity, and software package(s) used.	na
	13e	Describe any methods used to explore possible causes of heterogeneity among study results (e.g. subgroup analysis, meta-regression).	na
	13f	Describe any sensitivity analyses conducted to assess robustness of the synthesized results.	na
Reporting bias assessment	14	Describe any methods used to assess risk of bias due to missing results in a synthesis (arising from reporting biases).	na
Certainty	15	Describe any methods used to assess certainty (or confidence) in the body of evidence for an outcome.	na

For peer review only - http://bmjopen.bmj.com/site/about/guidelines.xhtml

PRISMA 2020 Checklist

Section and Topic	Item #	Checklist item	Location where item is reported
assessment			
**RESULTS**			
Study selection	16a	Describe the results of the search and selection process, from the number of records identified in the search to the number of studies included in the review, ideally using a flow diagram.	7
	16b	Cite studies that might appear to meet the inclusion criteria, but which were excluded, and explain why they were excluded.	7
Study characteristics	17	Cite each included study and present its characteristics.	8-10
Risk of bias in studies	18	Present assessments of risk of bias for each included study.	na
Results of individual studies	19	For all outcomes, present, for each study: (a) summary statistics for each group (where appropriate) and (b) an effect estimate and its precision (e.g. confidence/credible interval), ideally using structured tables or plots.	na
Results of syntheses	20a	For each synthesis, briefly summarise the characteristics and risk of bias among contributing studies.	na
	20b	Present results of all statistical syntheses conducted. If meta-analysis was done, present for each the summary estimate and its precision (e.g. confidence/credible interval) and measures of statistical heterogeneity. If comparing groups, describe the direction of the effect.	na
	20c	Present results of all investigations of possible causes of heterogeneity among study results.	na
	20d	Present results of all sensitivity analyses conducted to assess the robustness of the synthesized results.	na
Reporting biases	21	Present assessments of risk of bias due to missing results (arising from reporting biases) for each synthesis assessed.	na
Certainty of evidence	22	Present assessments of certainty (or confidence) in the body of evidence for each outcome assessed.	na
**DISCUSSION**			
Discussion	23a	Provide a general interpretation of the results in the context of other evidence.	10
	23b	Discuss any limitations of the evidence included in the review.	12
	23c	Discuss any limitations of the review processes used.	12
	23d	Discuss implications of the results for practice, policy, and future research.	13
**OTHER INFORMATION**			
Registration and protocol	24a	Provide registration information for the review, including register name and registration number, or state that the review was not registered.	4
	24b	Indicate where the review protocol can be accessed, or state that a protocol was not prepared.	4
	24c	Describe and explain any amendments to information provided at registration or in the protocol.	na
Support	25	Describe sources of financial or non-financial support for the review, and the role of the funders or sponsors in the review.	13
Competing interests	26	Declare any competing interests of review authors.	13
Availability of data, code and other materials	27	Report which of the following are publicly available and where they can be found: template data collection forms; data extracted from included studies; data used for all analyses; analytic code; any other materials used in the review.	Table 2 and na

For peer review only - http://bmjopen.bmj.com/site/about/guidelines.xhtml

From: Page MJ, McKenzie JE, Bossuyt PM, Boutron I, Hoffmann TC, Mulrow CD, et al. The PRISMA 2020 statement: an updated guideline for reporting systematic reviews. BMJ 2021;372:n71. doi: 10.1136/bmj.n71

For peer review only

PRISMA 2020 Checklist

For more information, visit: http://www.prisma-statement.org/

For peer review only

How do pilot and feasibility studies inform randomised placebo-controlled trials in surgery? A systematic review

Journal:	*BMJ Open*
Manuscript ID	bmjopen-2022-071094.R1
Article Type:	Original research
Date Submitted by the Author:	25-May-2023
Complete List of Authors:	Cousins, Sian; University of Bristol, Population Health Sciences<br>Gormley, Alexander; University of Bristol, Dental School<br>Chalmers, Katy; University of Bristol<br>Campbell, Marion; University of Aberdeen, Health Services Research Unit<br>Beard, David; University of Oxford, Nuffield Dept of Orthopaedics, Rheumatology and Musculoskeletal Sciences<br>Blencowe, Natalie; University of Bristol, Bristol Centre for Surgical Research<br>Blazeby, Jane; University of Bristol
**Primary Subject Heading**:	Surgery
Secondary Subject Heading:	Research methods
Keywords:	SURGERY, Clinical Trial, Feasibility Studies

SCHOLARONE™ Manuscripts

I, the Submitting Author has the right to grant and does grant on behalf of all authors of the Work (as defined in the below author licence), an exclusive licence and/or a non-exclusive licence for contributions from authors who are: i) UK Crown employees; ii) where BMJ has agreed a CC-BY licence shall apply, and/or iii) in accordance with the terms applicable for US Federal Government officers or employees acting as part of their official duties; on a worldwide, perpetual, irrevocable, royalty-free basis to BMJ Publishing Group Ltd ("BMJ") its licensees and where the relevant Journal is co-owned by BMJ to the co-owners of the Journal, to publish the Work in this journal and any other BMJ products and to exploit all rights, as set out in our licence.

The Submitting Author accepts and understands that any supply made under these terms is made by BMJ to the Submitting Author unless you are acting as an employee on behalf of your employer or a postgraduate student of an affiliated institution which is paying any applicable article publishing charge ("APC") for Open Access articles. Where the Submitting Author wishes to make the Work available on an Open Access basis (and intends to pay the relevant APC), the terms of reuse of such Open Access shall be governed by a Creative Commons licence – details of these licences and which Creative Commons licence will apply to this Work are set out in our licence referred to above.

Other than as permitted in any relevant BMJ Author's Self Archiving Policies, I confirm this Work has not been accepted for publication elsewhere, is not being considered for publication elsewhere and does not duplicate material already published. I confirm all authors consent to publication of this Work and authorise the granting of this licence.

For peer review only

How do pilot and feasibility studies inform randomised placebo-controlled trials in surgery? A systematic review

S. Cousins[1], A. Gormley[1], K. Chalmers[1], M. K. Campbell[2], D. J. Beard[3,4,5], N. S Blencowe[1], J. M. Blazeby[1]

[1]Surgical innovation theme, Bristol National Institute for Health and Care Research (NIHR) Biomedical Research Centre, Centre for Surgical Research, Population Health Sciences, Bristol Medical School, Canynge Hall, Whatley Road, Bristol. BS8 2PS

[2]Health Services Research Unit, University of Aberdeen, Aberdeen, UK

[3]Nuffield Department of Orthopaedics, Rheumatology and Musculoskeletal Sciences, NIHR Biomedical Research Centre, University of Oxford, Oxford, UK

[4]NIHR Biomedical Research Centre, Oxford University Hospitals NHS Foundation Trust, Oxford, UK

[5]Royal College of Surgeons (England) Surgical Interventional Trials Unit (SITU), Botnar Research Centre, University of Oxford, Headington, Oxford, UK

Corresponding author - Sian.cousins@bristol.ac.uk

Sources of funding: National Institute for Health and Care Research Biomedical Research Centre at University Hospitals Bristol and Weston NHS Foundation Trust and the University of Bristol. The views expressed are those of the author(s) and not necessarily those of the NIHR or the Department of Health and Social Care. The study funders had no role in the design of the study, data collection, data analysis, interpretation of data, writing of the report or the decision to submit the article for publication.

Submission category – Review

Keywords – pilot, feasibility, surgery, invasive procedures, placebo, complex intervention

For peer review only

ABSTRACT

Introduction

Randomised controlled trials with a placebo comparator are considered the gold standard study design when evaluating healthcare interventions. These are challenging to design and deliver in surgery. Guidance recommends pilot and feasibility work to optimise main trial design and conduct, however, the extent to which this occurs in surgery is unknown.

Method

A systematic review identified randomised placebo-controlled surgical trials. Articles published from database inception to 31st December 2020 were retrieved from Ovid-MEDLINE, Ovid-EMBASE and CENTRAL electronic databases, hand-searching and expert knowledge. Pilot/feasibility work conducted prior to the RCTs was then identified from examining citations and reference lists. Where studies explicitly stated their intent to inform the design and/or conduct of the future main placebo-controlled surgical trial, they were included. Publication type, clinical area, treatment intervention, number of centres, sample size, comparators, aims, and text about the invasive placebo intervention were extracted.

Results

From 131 placebo surgical RCTs included in the systematic review, 47 potentially eligible pilot/feasibility studies were identified. Of these, four were included as true pilot/feasibility work. Three were original articles, one a conference abstract; three were conducted in orthopaedic surgery and one in oral and maxillofacial surgery. All four included pilot RCTs, with an invasive surgical placebo intervention, randomising 9 to 49 participants in 1 or 2 centres. They explored the acceptability of recruitment and the invasive placebo intervention to patients and trial personnel, and whether blinding was possible. One study examined the characteristics of the proposed invasive placebo intervention using in-depth interviews.

Conclusion

For peer review only - http://bmjopen.bmj.com/site/about/guidelines.xhtml

Published studies reporting feasibility/pilot work undertaken to inform main placebo surgical trials are scarce. In view of the difficulties of undertaking placebo surgical trials, it is recommended that pilot/feasibility studies are conducted, and more are reported to share key findings and optimise the design of main RCTs.

PROSPERO REGISTRATION NUMBER – CRD42021287371

STRENGTHS AND LIMITATIONS OF THIS STUDY

- To our knowledge, this work is original and is the first review to use systematic methods to identify and examine feasibility and pilot work to inform main surgical placebo RCTs
- This work has been performed to a high standard; rigorous searches were undertaken and all articles were screened by two reviewers
- The current review searches are limited to the published literature and it is possible that feasibility and pilot work that may have been conducted but not published was not included.

INTRODUCTION

Randomised controlled trials (RCTs) with a placebo comparator have the potential to answer key clinical questions regarding the effectiveness of invasive procedures, including surgery. Indeed, exemplar studies have been undertaken that have informed clinical practice.(1, 2) Placebo-controlled trials in surgery and other invasive procedures are, however, challenging. There are ethical issues (3, 4) related to potential risk to patients, and practical issues regarding the acceptability of the design of the invasive placebo comparator to patients and trial personnel.

For peer review only - http://bmjopen.bmj.com/site/about/guidelines.xhtml

Current guidance for the development of complex healthcare interventions (5) and for the design of placebo surgical trials (6) supports feasibility and pilot work to optimise evaluation in a main RCT. Pilot and feasibility work can address uncertainties related to the integrity of the study protocol, recruitment and retention, outcome measures, randomisation procedures, as well as development and acceptability of the intervention itself.(7, 8) This is particularly important for placebo surgical trials where acceptability and feasibility may be more challenging. It is unknown to what extent such preparatory work is conducted prior to main surgical placebo trials.

The aim of this review is to examine the extent and type of publications reporting feasibility and pilot work conducted to inform main placebo-controlled trials of surgery, and identify exemplar studies to inform future work.

METHODS

Published studies reporting feasibility/pilot work conducted in preparation for main randomised placebo-controlled surgical trials was identified through examination of RCTs retrieved by a systematic review (PROSPERO registration CRD42021287371). Feasibility/pilot studies identified were then examined in-depth, as detailed below.

Systematic review of placebo-controlled randomised surgical trials

An existing review (9) which identified placebo surgical trials was updated by extending the searches to 31st December 2020. Searches used the same search terms and electronic databases (Ovid MEDLINE, Ovid EMBASE, and CENTRAL).(10)(Supplementary material 1). Additional articles, with no restriction on publication date were identified by hand searching and expert knowledge.

Eligibility criteria – Placebo-controlled randomised surgical trials

Eligibility criteria are described fully in Cousins et al. 2019.(9) Briefly, articles reporting randomised trials (including follow-ups, protocols and any self-reported as 'pilot' RCTs) comparing surgery with placebo interventions were included. Surgery was defined as any invasive procedure that changes the

For peer review only - http://bmjopen.bmj.com/site/about/guidelines.xhtml

anatomy and requires a skin incision or use of endoscopic techniques.(11) Placebo interventions referred to any surgical procedure that was intended to mimic the treatment intervention under evaluation. This included placebo interventions of all types regardless of the degree of invasiveness. Pharmaceutical or dental interventions and reviews were excluded. Protocols of all included studies were retrieved, where available.

Identifying published pilot/feasibility studies conducted to inform placebo-controlled randomised surgical trials

Main trial publications identified by the systematic review were read fully, including protocols and clinical trials registry entries, where available. Potentially eligible feasibility/pilot studies were identified in two ways. In the first instance, studies referred to explicitly in the main trial text using the terms 'feasibility' or 'pilot', with associated reference(s) were retrieved. Where references were not provided, publication lists of the corresponding author were hand-searched to identify any publications related to work undertaken before the main trial that were cited as being relevant to the feasibility/pilot work mentioned. Secondly, reference lists of the main trials were hand-searched to identify studies self-reported in the title as 'feasibility' or 'pilot' studies. The use of these terms to identify feasibility/pilot work is supported by a study conducted by Eldridge et al 2016 that aimed to develop a conceptual framework for defining feasibility and pilot studies.(7) The study found that of 27 studies identified undertaken in preparation for a RCT, all used at least one of these terms in their titles..(7) Main trial texts were examined by two reviewers (SC and AG) independently. RCTs included in the systematic review update that were self-reported as 'pilot' were also examined for eligibility. Scoping searches on Medline (Ovid SP) electronic database using the search concepts 'feasibility/pilot', 'surgery' and 'placebo', combined with 'and' but with no additional filters, did not retrieve any relevant results. Therefore, additional systematic searches of electronic databases for pilot and feasibility studies were not conducted.

Eligibility criteria – Feasibility and pilot studies to inform main RCTs

For peer review only - http://bmjopen.bmj.com/site/about/guidelines.xhtml

For the purposes of this review, eligible studies were those that included an explicit statement that the work was to inform the design and/or conduct of a future main placebo-controlled surgical trial (irrespective of whether the study was labelled or entitled pilot/feasibility or if it had been referred to as pilot/feasibility work in the main trial text). Studies of any design, with aims including, but not limited to, the acceptability of interventions to patients and clinicians, recruitment and retention, and development of invasive placebo interventions, were included. Internal pilot studies (those which solely tested the finalised design as part of the main trial) and those primarily assessing efficacy/effectiveness/safety outcomes were excluded. Original articles and conference abstracts were included; letters, editorials, and reviews were excluded. At least two reviewers (SC, AG, KC) screened identified studies independently to ensure they met the above eligibility criteria. Disagreements were resolved by consensus or by a 3rd senior reviewer (JMB), if necessary. Where included feasibility/pilot publications referenced additional related publications, these were retrieved to inform data extraction.

Data extraction and analysis

Data extracted from included RCTs identified by the systematic review update were: year of publication, region, clinical area (e.g., gastrointestinal), number of centres, number of patients randomised, and treatment intervention. Data extracted from included feasibility/pilot studies were: publication type (original article or abstract), clinical area and treatment intervention, study design, number of centres, sample size, comparison groups, reported study aims, and any text related to the invasive placebo intervention, specifically regarding work done to inform its development and whether studies reported criteria against which decisions would be made to progress to a main RCT. Data were extracted by one reviewer using a standardised data extraction form. A second reviewer extracted data for 20% of articles to identify any potential systematic errors in data extraction. Where multiple articles related to the same study, they were grouped into a single set, and data extraction was conducted on a "per trial" (rather than "per article") basis. Descriptive statistics summarised basic data where appropriate and verbatim text was summarised descriptively.

For peer review only - http://bmjopen.bmj.com/site/about/guidelines.xhtml

Patient and public involvement

No patient involved

RESULTS

Placebo-controlled randomised trials of invasive procedures

The 96 articles identified by the previous review(9) were added to those identified in the current review update. Searches retrieved 1918 articles, of which 113 full texts were screened and 35 trials included (Figure 1). Finally included were 131 trials (Supplementary material 2); this included four RCTs that were self-reported as 'pilot' identified by the systematic review update. Characteristics are shown in Table 1.

For peer review only

For peer review only - http://bmjopen.bmj.com/site/about/guidelines.xhtml

Table 1. Characteristics of placebo-controlled randomised controlled trials of invasive procedures identified in the systematic review update

Characteristic	Number of RCTs, n=131 (%)
Year of publication	
≤2000	28 (21)
2001-2010	38 (29)
2011-2020	65 (50)
Region	
United States	44 (34)
Mainland Europe	32 (24)
United Kingdom	17 (13)
Australia	9 (7)
Asia	7 (5)
Canada	3 (2)
South America	1 (1)
Multi-region	18 (14)
Clinical area	
Gastrointestinal	44 (34)
Orthopaedics and trauma	28 (21)
Oral and maxillofacial	22 (17)
Interventional cardiology	10 (8)
Cardiothoracic	7 (5)
Neurosurgery	6 (5)
Gynaecology and obstetrics	5 (4)
Ophthalmology	4 (3)
Podiatry	3 (2)
Urology	2 (2)
Number of centres	
1	44 (34)
2-5	25 (19)
6-10	9 (7)
>10	22 (17)
Not reported	31 (24)
Number of patients randomised[a]	
1-100	82 (63)
101-200	23 (18)
>200	20 (15)
Treatment intervention	
Endoscopic	50 (38)
Minimal access	36 (27)
Percutaneous	31 (24)
Open surgery	14 (11)

[a]n=6 included protocols (number of patients randomised not reported)

Feasibility and pilot studies conducted to inform main placebo-controlled surgical RCTs

Of the 127 main placebo RCTs identified (Figure 2), 29 referenced studies explicitly referred to as ‘feasibility’ or ‘pilot’ studies in the main trial text. Hand-searching references of the papers found a further 14 studies self-reported as ‘feasibility’ or ’pilot’ studies in the title. These were combined with the four RCTs self-reported as ‘pilot’ identified in the systematic review (total 47 papers examined).

For peer review only - http://bmjopen.bmj.com/site/about/guidelines.xhtml

206 In-depth reading of these found that the majority (n=37) did not report pilot or feasibility work to inform a placebo-controlled main RCT and so were excluded. Although they had been referred to, or were self-reported as, pilot or feasibility studies, there was no explicit statement in the whole report that the work was intended to inform a subsequent main placebo-controlled surgical trial. All of these 37 papers wrote about intentions to examine treatment effects. Two papers reporting 'internal pilot' studies, two duplicates (12, 13) (papers that were identified twice, and are included below) and one review (14) and letter (15) were also excluded.

213 Four studies were explicit in their intention to inform main placebo surgical RCTs design and conduct and were included (12, 13, 16, 17). All self-reported as a pilot or feasibility study in the title and outlined aims related to their intention to inform a future main placebo-controlled surgical RCT in their introduction. Study details are summarised in Table 2 and described individually in detail below.

For peer review only

Table 2. Study details of feasibility and pilot studies identified to inform main placebo surgical RCTs

Author (Year)	Publication type	Clinical area (Treatment intervention)	Study design (Comparator group)	# centers	Sample size	Reported study aims
Campbell (2011)	Original article	Orthopaedics (Arthroscopic lavage for knee osteoarthritis)	Qualitative interviews and focus groups	NA	257	Examine the need for a placebo-controlled main RCT; whether an appropriate placebo can be designed (including surgical and anesthetic components); and the acceptability of an invasive placebo to patients, clinicians and ethics committee chairs
			Survey	NA	780	Examine acceptability of an invasive placebo to health professionals
			Pilot RCT (Invasive placebo and no treatment)	2	49	Feasibility of recruitment
Kallmes (2002)	Conference abstract	Orthopaedics (Percutaneous vertebroplasty for osteoporotic spinal fractures)	Pilot RCT (Invasive placebo)	NR	5	Feasibility of recruitment
Powell (2001)	Original article	Oral and maxillofacial (Radiofrequency reduction of turbinate hypertrophy for sleep-disordered breathing)	Pilot RCT (Invasive placebo)	1	22	Estimate treatment effect and impact of study design<br>Determine appropriate study design of main trial<br>Feasibility of conducting main RCT
Moseley (1996)	Original article	Orthopaedics (Arthroscopic debridement for knee osteoarthritis)	Pilot RCT (Invasive placebo)	1	10	Examine the need for a placebo-controlled main RCT<br>Feasibility of recruitment<br>Develop and test outcome measures<br>Acceptability of placebo<br>Ability of placebo to blind patients

NA, Not applicable; RCT, Randomised controlled trial; NR, Not reported

For peer review only

For peer review only - http://bmjopen.bmj.com/site/about/guidelines.xhtml

1. Campbell et al. 2011 (17, 18)

This mixed methods study, published as an original article and identified by the systematic review update, examined the feasibility of a main trial evaluating arthroscopic lavage compared with placebo surgery and non-operative management for patients with knee osteoarthritis. An initial exploratory phase consisted of in-depth qualitative (interviews and focus groups) (n=257) and quantitative (postal surveys) work (n=780) to explore views and opinions about a main trial. Issues of the design of the invasive placebo comparator, including surgical and anaesthetic components, and the acceptability of this to key stakeholders, including patients, health professionals and chairs of ethics committees were explored. Broadly, all stakeholders agreed there was a need to investigate arthroscopic lavage further and surgeons and patients expressed uncertainty about the overall effectiveness of the technique. Discussion regarding the design of a placebo intervention centred around which invasive components were required ("The consensus emerged fairly readily that three superficial skin incisions were needed, that these should only pierce the epidermis, and that any penetration of the knee capsule should be avoided", and what form anesthesia should take. There was general agreement that, assuming general anesthesia was adopted, inclusion should be limited to low risk patients. Findings from the survey supported the insights gleaned from discussion in the focus groups. A pilot RCT was also conducted. This randomised 49 patients from two centres to examine the feasibility of the proposed placebo-controlled design, including gaining ethical and local approvals and recruitment of patients and delivering placebo surgery. Decisions about whether to progress to a main RCT were reported. The authors concluded that a main placebo surgical trial could be successfully designed and was generally acceptable, however the considerable barriers faced in conducting the trial in practice meant a main RCT was not feasible and did not take place. These barriers mainly concerned gaining local clinical approval at sites, even after ethical approval was secured. There were concerns at regarding indemnity and who would pay for placebo procedures, as well as about the inclusion of surgeons or centres that do not usually offer arthroscopic lavage.

For peer review only - http://bmjopen.bmj.com/site/about/guidelines.xhtml

2. *Kallmes et al 2002 (16)*

This study, published as a conference abstract, examined whether recruitment to a trial comparing vertebroplasty or a placebo for osteoporotic spinal fractures was possible. This was assessed within a pilot RCT that randomised five patients in one centre to treatment or placebo interventions. The placebo intervention included fluoroscopically guided placement of a 25-gauge needle and infiltration of the pedicle with Bupivicaine, without placebo of either the vertebroplasty needle or cement, as in the treatment group. Although the methacrylate monomer was opened in the procedure room to give patients olfactory cues and simulate cement preparation. Localised pressure was also placed on the back of patients in the placebo group and operators gave verbal cues typical during cement injection. Although not specified as an explicit aim, the study reported blinding success by asking participants to guess which procedure they had received; all 5 patients guessed they had received the placebo intervention. The authors concluded that enrolment of patients into a main placebo-controlled trial was feasible.(19)

3. *Powell et al 2001* (12)

This single centre study identified by the systematic review update aimed to assess the impact of inclusion of an invasive placebo comparator on estimates of treatment effect. A pilot RCT randomised 22 participants with sleep disordered breathing to temperature-controlled radiofrequency reduction of turbinate hypertrophy or an invasive placebo. The placebo intervention was identical to treatment, with electrode placement into the anterior inferior turbinate, except that a separate unblinded investigator used a covert radiofrequency energy cut-off switch, to ensure none was applied. The study compared outcomes between blinded and unblinded assessors. It found that unblinded assessment yielded greater treatment effect (bias) and this was used as rationale by the authors for the need to include a placebo comparator in a main trial. Although not explicitly outlined as a study aim authors commented that it was feasible to design a placebo procedure with only the 'active ingredient' withheld. The authors argued that the treatment creates minimal morbidity and it was ethical to conduct a placebo trial because the treatment was not yet the standard of care and there

For peer review only - http://bmjopen.bmj.com/site/about/guidelines.xhtml

was clinical equipoise. A placebo intervention was also deemed feasible because there were no obvious distinguishable characteristics of treatment, in that its effect was subtle. The authors concluded that a future definitive study was feasible and inclusion of a placebo was critical, however it is unclear whether it took place as no main trial publication could be found.

4. *Moseley et al 1996* (13)

This study, published as an original article, aimed to determine whether a placebo control was necessary in the main RCT, the feasibility of recruitment, the ability of the placebo to blind participants and outcome assessors, and the satisfaction of patients allocated to placebo. They conducted a pilot RCT of 10 patients in one centre. The study interventions included arthroscopic debridement of the knee for osteoarthritis, arthroscopic lavage or an invasive placebo procedure. Patients randmoised to the placebo received a lesser anesthetic and did not have an endotracheal tube placed, compared to the two treatment groups that underwent general anesthetic and placement of an endotracheal tube. The authors reflected that using sedation and local anesthetic in the placebo group minimised potential complications. Three incisions were made with a scalpel in the placebo intervention, but no instruments were placed in the knee. The knee was, however, manipulated and instruments requested by operators with saline splashed to simulate arthroscopic debridement. Post-operative management was the same across all groups. Surgeons also dictated two operative notes, one for the hospital chart not specifying which procedure was undertaken, and one kept separately by the surgeon detailing the procedure delivered. Although these measures were taken to maintain blinding, authors did not comment specifically on how the invasive placebo was developed. Patient satisfaction was assessed by asking the questions 'would you recommend the surgery to your friends and family?' and 'do you think the operation was worthwhile?'. Most patients were satisfied in the postoperative period, and at 6 months, seven of the nine patients would recommend the surgery to friends or family. The success of blinding participants and personnel was examined by asking participants and physicians to guess which procedure was performed at all post-operative visits. Patients and outcome assessors were unable to consistently guess which procedure had been delivered. The authors commented that

For peer review only - http://bmjopen.bmj.com/site/about/guidelines.xhtml

getting study approvals from the necessary committees and institutions was a slow process, with approvals gained only after it was made clear that participants would be fully informed about the placebo-controlled nature of the study. The authors concluded that the main RCT should include an invasive placebo procedure, indeed that failure to use a placebo would “seriously impair our ability to draw valid inference from the proposed study”, and that recruitment was feasible, as was the ability of the placebo to blind patients. A main trial was completed, randomising 180 patients.(1)

DISCUSSION

Placebo controlled trials in surgery are challenging to design and deliver and can be contentious. Guidance recommends that pre-trial pilot and feasibility work be undertaken to optimise the design and conduct of the trial. Placebo-controlled surgical RCTs identified by the systematic review were rigorously and systematically examined to identify feasibility or pilot work conducted and published to inform the main trial design and conduct. Of the 132 RCTs identified, 47 referred to or referenced ‘feasibility’ or ‘pilot’ studies. On detailed scrutiny the vast majority did not state any intention to inform a main placebo-controlled randomised trial, assessing instead the effectiveness/efficacy of the treatment intervention. Four feasibility/pilot studies did outline aims to inform methodological aspects of the main RCT. These focussed on key uncertainties of recruitment, the need for a placebo comparator in the main RCT, the ability of the invasive placebo to blind participants and trial persons, the acceptability of an invasive placebo comparator to patients and clinicians, and in one study, the potential components/design of the invasive placebo. All four of the feasibility/pilot studies included a pilot RCT. One (17, 18) also employed interviews and focus groups, and a postal survey to examine the design and acceptability of an invasive placebo. The identified studies highlighted the importance of preparatory work and how it can have a major influence on the design of the definitive placebo-surgical trial, both in shaping the final design of the placebo ensuring that it is fit for purpose and able to blind trial persons effectively and informing the feasibility (or otherwise) of progressing to a main trial. Indeed, one of the studies (18) asserted that a main trial was not feasible; avoiding valuable research resources if a main trial had been conducted that was not successful. The current work found

For peer review only - http://bmjopen.bmj.com/site/about/guidelines.xhtml

that main placebo-controlled surgical trials are not often informed by published pilot and feasibility studies. It is recommended that feasibility work is conducted and published, not only to optimise the design and conduct of main placebo surgical RCTs, but also to reduce research waste and share lessons learnt.

The limited feasibility and pilot work assessing methodological considerations of placebo surgical RCTs may be due to a historical lack of clarity about the meaning and design of 'pilot' and 'feasibility' studies.(20) Commonly pilot studies have been labelled as such to justify small sample sizes, rather than explore the feasibility of conducting a main RCT. It may also be due to challenges in publishing these studies due to editorial policy; so these studies may have been conducted but not published.(21) Recent work has made strides in clarifying definitions (22, 23) and highlights feasibility aims appropriate for assessment in feasibility/pilot studies.(7) The publication of guidance for the reporting of pilot and feasibility studies, including the pilot and feasibility extension of the Consolidated Standards of Reporting Trials (23-25), and the emergence of journals specific to this area (26) may facilitate the optimisation of the design, reporting and publication of these studies. Guidance for using qualitative research in feasibility studies for trials has also been published.(27)) This consists of a list of 16 items within five domains (research questions, data collection, analysis, teamworking and reporting) that should be considered when assessing or undertaking qualitative research within feasibility studies for RCTs. Qualitative work has the propensity to add rich information about intervention acceptability and feasibility of delivery; issues paramount when developing and piloting invasive placebo interventions.

Specific to the design and conduct of surgical placebo-controlled trials, published guidelines (6) recommend pilot work to inform the design of invasive placebo procedures, and provides details about how treatment interventions may be deconstructed to identify critical surgical element/s (that can then be omitted from the placebo).(28) The controversial nature of surgical and invasive interventions means that feasibility and pilot work is of the utmost importance. There are issues

For peer review only - http://bmjopen.bmj.com/site/about/guidelines.xhtml

around the acceptability of the trial to patients, trial personnel and ethics committees due to perceived potential risk. Designing an invasive placebo is also challenging and feasibility work should include examination of which components of the surgery should be included (and omitted) from the invasive placebo intervention and whether it is able to effectively blind participants and trial personnel to trial group allocation.

Inappropriate emphasis on hypothesis testing within pilot and feasibility work, rather than the methodological aspects of the design of a future main RCT is shown in other reviews of published literature. Shanyinde (29) identified studies with 'pilot' or 'feasibility' in the title published between 2000-2009 and found that of the 50 papers sampled 56% examined methodological issues in-depth and 18% discussed a future trial. Arain et al (21), who examined published studies found with the keywords 'pilot' and 'feasibility' between 2007 and 2008, found that of the 54 studies identified, 21 reported hypothesis testing and performed statistics to report significant results. Arain et al also searched the United Kingdom Clinical Research Network portfolio for feasibility/pilot studies and of the 34 identified, only 12 tested some component of the research process. A review examining feasibility and pilot studies of surgical interventions funded by the United Kingdom National Institute for Health and Care Research programmes from 2005 to 2015 (30) found that although over half of the 35 studies identified examined methodological components, such as recruitment or the current intervention, fewer looked at aspects specific to surgery (n=10). Another review examining published 'pilot' RCTs in orthopaedic surgery found that of the 49 studies included, the majority (n=28) evaluated the efficacy of the intervention.(31)

The current work provides an update to previous systematic reviews of placebo-controlled randomised surgical trials. Further examination of the trials identified by the review update is needed to examine in-depth the methodological aspects, especially as they relate to the ASPIRE recommendations(6), which was published after the previous review(9). This was outside the scope of this work, but is currently underway. Main trial documents, including protocols and clinical trials

For peer review only - http://bmjopen.bmj.com/site/about/guidelines.xhtml

registries, were rigorously examined by two independent reviewers to identify feasibility and pilot work. This review did not however, search for pilot and feasibility studies specifically using electronic databases; although scope searches using search concepts ‘feasibility/pilot’, ‘surgery’ and ‘placebo’ did not identify any additional relevant feasibility/pilot studies. This review was also restricted to published feasibility and pilot studies; it is possible that studies may have been conducted and not published and contacting authors of main placebo controlled trials may be one way to examine this. The exemplar papers identified may be used to inform future feasibility studies in this area. These studies provide useful details about the design and conduct of placebo surgical RCTs specifically, including potential study aims (e.g., the ability of the proposed invasive placebo to blind participants and trial persons) and the use of qualitative methods that may inform future RCTs.

There is a dearth of feasibility and pilot work conducted and published to inform the design and conduct of placebo surgical RCTs. Given the challenging nature of these studies, including practical and ethical considerations, feasibility and pilot studies are needed. These will ensure main RCTs are feasible and that the proposed invasive placebo interventions are acceptable and effective in blinding participants and trial persons.

AUTHOR CONTRIBUTIONS

SC: conceptualisation; methodology; formal analysis; supervision; writing – original draft; AG: formal analysis; writing – review and editing; KC: formal analysis; writing – review and editing; MKC: conceptualisation; methodology; writing – review and editing; DJB: conceptualisation; methodology; writing – review and editing; NSB: conceptualisation; methodology; writing – review and editing; JMB: funding acquisition; conceptualisation; metholodology; formal analysis; supervision; writing – review and editing.

COMPETING INTERESTS

The authors declare no conflicts of interest and the funder of this study had no involvement in study design, data collection, data analysis, or manuscript preparation.

For peer review only - http://bmjopen.bmj.com/site/about/guidelines.xhtml

FUNDING

This work was supported by the National Institute for Health and Care Research Biomedical Research Centre at University Hospitals Bristol and Weston NHS Foundation Trust and the University of Bristol (BRC-1215-20011).

DATA SHARING STATEMENT

All data relevant to the study are included in the article or uploaded as supplementary information

ETHICS APPROVAL STATEMENT

The study did not include human participants and ethical approval was not required.

References

1. Moseley JB, O'Malley K, Petersen NJ, Menke TJ, Brody BA, Kuykendall DH, et al. A controlled trial of arthroscopic surgery for osteoarthritis of the knee. N Engl J Med. 2002;347(2):81-8.
2. Beard DJ, Rees JL, Cook JA, Rombach I, Cooper C, Merritt N, et al. Arthroscopic subacromial decompression for subacromial shoulder pain (CSAW): a multicentre, pragmatic, parallel group, placebo-controlled, three-group, randomised surgical trial. Lancet. 2018;391(10118):329-38.
3. Horng S, Miller FG. Ethical framework for the use of sham procedures in clinical trials. Crit Care Med. 2003;31(3 Suppl):S126-30.
4. Savulescu J, Wartolowska K, Carr A. Randomised placebo-controlled trials of surgery: ethical analysis and guidelines. J Med Ethics. 2016;42(12):776-83.
5. Skivington K, Matthews L, Simpson SA, Craig P, Baird J, Blazeby JM, et al. A new framework for developing and evaluating complex interventions: update of Medical Research Council guidance. Bmj. 2021;374:n2061.
6. Beard DJ, Campbell MK, Blazeby JM, Carr AJ, Weijer C, Cuthbertson BH, et al. Considerations and methods for placebo controls in surgical trials (ASPIRE guidelines). Lancet. 2020;395(10226):828-38.
7. Eldridge SM, Lancaster GA, Campbell MJ, Thabane L, Hopewell S, Coleman CL, et al. Defining Feasibility and Pilot Studies in Preparation for Randomised Controlled Trials: Development of a Conceptual Framework. PLoS One. 2016;11(3):e0150205.
8. Lancaster GA, Dodd S, Williamson PR. Design and analysis of pilot studies: recommendations for good practice. J Eval Clin Pract. 2004;10(2):307-12.
9. Cousins S, Blencowe NS, Tsang C, Lorenc A, Chalmers K, Carr AJ, et al. Reporting of key methodological issues in placebo-controlled trials of surgery needs improvement: a systematic review. J Clin Epidemiol. 2020;119:109-16.
10. Goossen K, Tenckhoff S, Probst P, Grummich K, Mihaljevic AL, Buchler MW, et al. Optimal literature search for systematic reviews in surgery. Langenbecks Arch Surg. 2018;403(1):119-29.
11. Wartolowska K, Collins GS, Hopewell S, Judge A, Dean BJ, Rombach I, et al. Feasibility of surgical randomised controlled trials with a placebo arm: a systematic review. BMJ Open. 2016;6(3):e010194.
12. Powell NB, Zonato AI, Weaver EM, Li K, Troell R, Riley RW, et al. Radiofrequency treatment of turbinate hypertrophy in subjects using continuous positive airway pressure: a randomized, double-blind, placebo-controlled clinical pilot trial. Laryngoscope. 2001;111(10):1783-90.

For peer review only - http://bmjopen.bmj.com/site/about/guidelines.xhtml

13. Moseley JB, Jr., Wray NP, Kuykendall D, Willis K, Landon G. Arthroscopic treatment of osteoarthritis of the knee: a prospective, randomized, placebo-controlled trial. Results of a pilot study. The American journal of sports medicine. 1996;24(1):28-34.
14. Cook IJ. Diagnostic evaluation of dysphagia. Nature clinical practice Gastroenterology & hepatology. 2008;5(7):393-403.
15. Malek F, Neuzil P, Gustafsson F, Kaye DM, Walton A, Mates M, et al. Clinical outcome of transcatheter treatment of heart failure with preserved or mildly reduced ejection fraction using a novel implant. International journal of cardiology. 2015;187:227-8.
16. Kallmes DF, Jensen ME, Marx WF, Sinha Roy S, Schweickert PA, Jarvik JG. A Pilot Study for a Sham-Controlled, Randomized, Prospective, Crossover Trial of Percutaneous Vertebroplasty. American Society of Neuroradiology; May 13th; Vancouver2002.
17. Campbell MK, Entwistle VA, Cuthbertson BH, Skea ZC, Sutherland AG, McDonald AM, et al. Developing a placebo-controlled trial in surgery: issues of design, acceptability and feasibility. Trials. 2011;12:50.
18. Campbell MK, Skea ZC, Sutherland AG, Cuthbertson BH, Entwistle VA, McDonald AM, et al. Effectiveness and cost-effectiveness of arthroscopic lavage in the treatment of osteoarthritis of the knee: a mixed methods study of the feasibility of conducting a surgical placebo-controlled trial (the KORAL study). Health technology assessment (Winchester, England). 2010;14(5):1-180.
19. Kallmes DF, Comstock BA, Heagerty PJ, Turner JA, Wilson DJ, Diamond TH, et al. A randomized trial of vertebroplasty for osteoporotic spinal fractures. N Engl J Med. 2009;361(6):569-79.
20. Whitehead AL, Sully BG, Campbell MJ. Pilot and feasibility studies: is there a difference from each other and from a randomised controlled trial? Contemporary clinical trials. 2014;38(1):130-3.
21. Arain M, Campbell MJ, Cooper CL, Lancaster GA. What is a pilot or feasibility study? A review of current practice and editorial policy. BMC medical research methodology. 2010;10:67.
22. Research NIfH. Guidance on applying for feasibility studies 2021 [01/08/2021]. Available from: https://www.nihr.ac.uk/documents/nihr-research-for-patient-benefit-rfpb-programme-guidance-on-applying-for-feasibility-studies/20474.
23. Eldridge SM, Chan CL, Campbell MJ, Bond CM, Hopewell S, Thabane L, et al. CONSORT 2010 statement: extension to randomised pilot and feasibility trials. 2016;355:i5239.
24. Lancaster GA, Thabane L. Guidelines for reporting non-randomised pilot and feasibility studies. Pilot Feasibility Stud. 2019;5:114.
25. Thabane L, Lancaster G. A guide to the reporting of protocols of pilot and feasibility trials. Pilot Feasibility Stud. 2019;5:37.
26. Lancaster GA. Pilot and feasibility studies come of age! Pilot Feasibility Stud. 2015;1(1):1.
27. O'Cathain A, Hoddinott P, Lewin S, Thomas KJ, Young B, Adamson J, et al. Maximising the impact of qualitative research in feasibility studies for randomised controlled trials: guidance for researchers. Pilot Feasibility Stud. 2015;1:32.
28. Cousins S, Blencowe NS, Tsang C, Chalmers K, Mardanpour A, Carr AJ, et al. Optimizing the design of invasive placebo interventions in randomized controlled trials. Br J Surg. 2020.
29. Shanyinde M, Pickering RM, Weatherall M. Questions asked and answered in pilot and feasibility randomized controlled trials. BMC medical research methodology. 2011;11:117.
30. Fairhurst K, Blazeby JM, Potter S, Gamble C, Rowlands C, Avery KNL. Value of surgical pilot and feasibility study protocols. Br J Surg. 2019;106(8):968-78.
31. Desai B, Desai V, Shah S, Srinath A, Saleh A, Simunovic N, et al. Pilot randomized controlled trials in the orthopaedic surgery literature: a systematic review. BMC Musculoskelet Disord. 2018;19(1):412.

For peer review only - http://bmjopen.bmj.com/site/about/guidelines.xhtml

FIGURE LEGENDS

Figure 1. PRISMA diagram showing screening process of retrieved articles

Figure 2. PRISMA diagram showing screening process of feasibility and pilot studies

For peer review only

For peer review only - http://bmjopen.bmj.com/site/about/guidelines.xhtml

Figure 1. PRISMA diagram showing screening process of retrieved articles

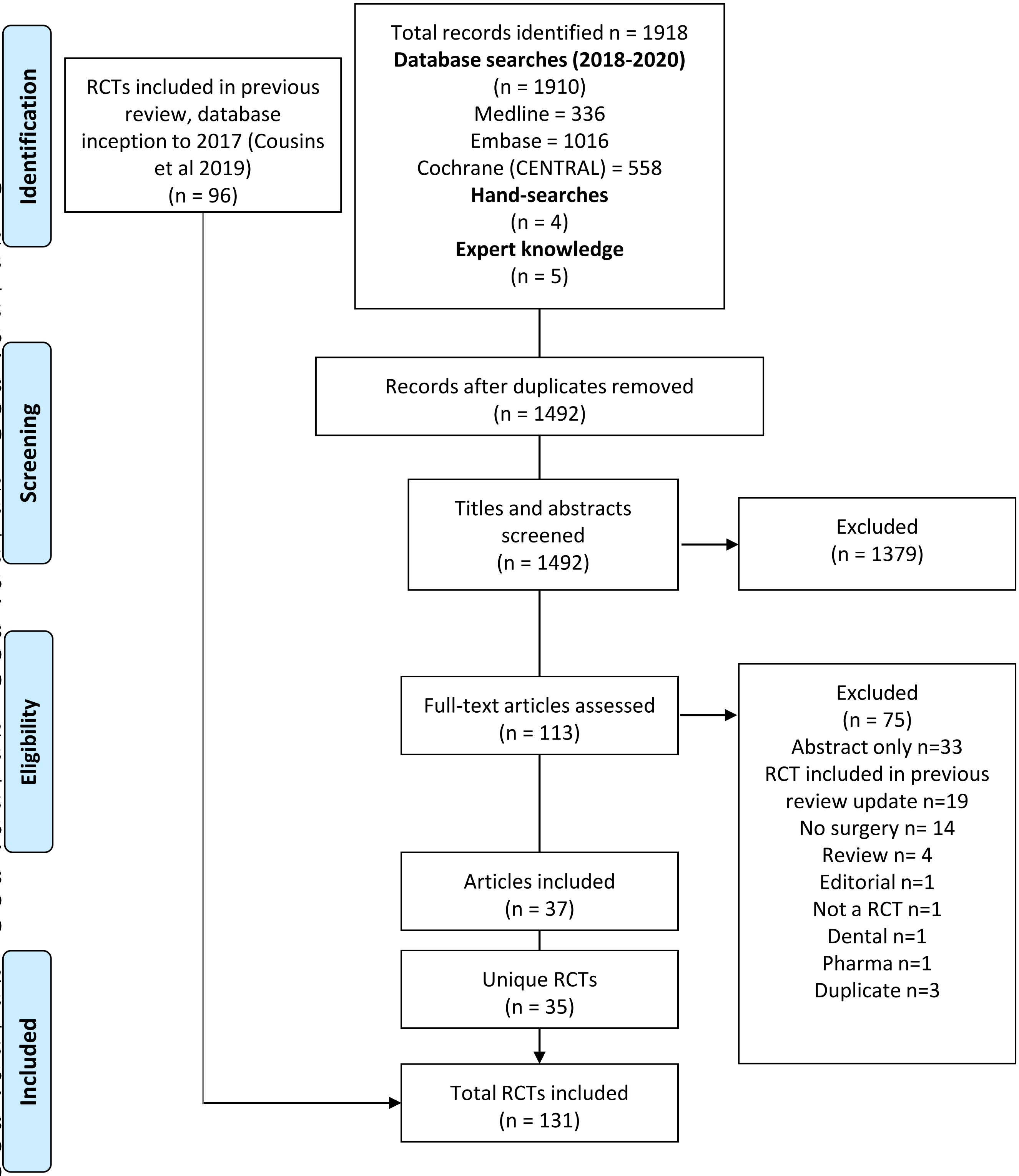

RCTs, Randomised Controlled Trials

Figure 2. PRISMA diagram showing screening process of feasibility and pilot studies

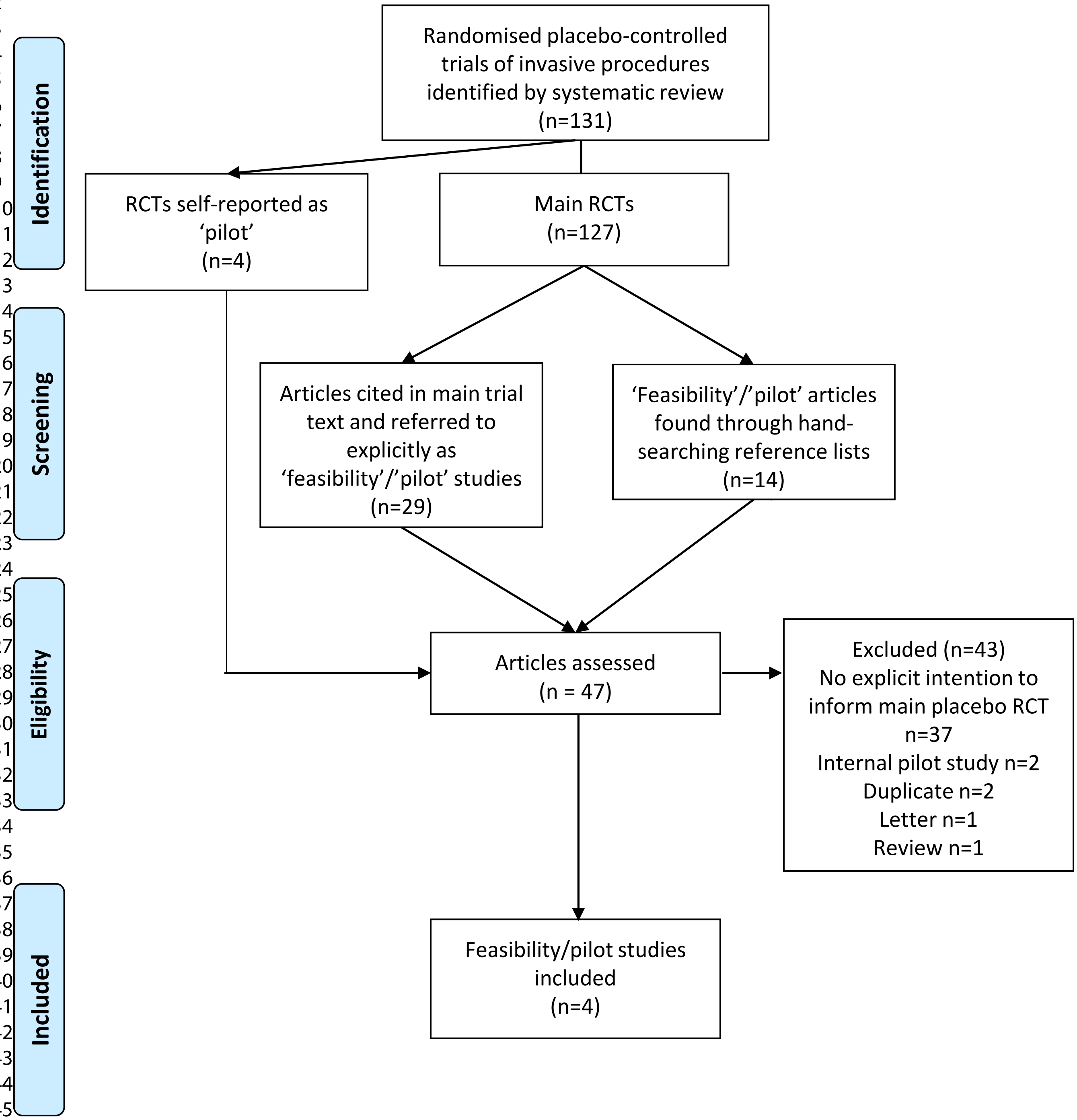

RCTs, Randomised Controlled Trials

Appendix A – Search terms uses in Ovid MEDLINE, Ovid EMBASE and CENTRAL databases

Ovid MEDLINE

1. Clinical trial/
2. Randomized controlled trial/
3. Randomization/
4. Rct.tw.
5. random allocation.tw.
6. Randomly allocated.tw.
7. Allocated randomly.tw.
8. Randomized Controlled Trials as Topic/
9. randomized controlled trial/
10. Double Blind Method/
11. Single Blind Method/
12. clinical trial/
13. controlled clinical trial.pt.
14. randomized controlled trial.pt.
15. clinical trial.pt.
16. exp Clinical Trials as topic/
17. or/1-16
18. PLACEBOS/
19. placebo$.tw.
20. sham.tw.
21. immitation.tw.
22. placebo effect$.tw.
23. or/18-22
24. surgery.tw.
25. surgical.tw.
26. arthroscopy.tw.
27. endoscopy.tw.
28. transplantation.tw.
29. $scopy.tw.

For peer review only

30. $scopic.tw.

31. laparoscopy.tw.

32. Meta-Analysis as Topic/

33. meta analy$.tw.

34. metaanaly$.tw.

35. Review/

36. Comment/

37. Letter/

38. Editorial/

39. animal/

40. dose$.tw.

41. pre$medication.tw.

42. an$esthesia.tw.

43. an$esthetic$.tw.

44. antibiotic$.tw.

45. steroid$.tw.

46. prophylaxis.tw.

47. prevention.tw.

48. preoperative.tw.

49. preanaesthetic$.tw.

50. pre$emptive.tw.

51. pre-operative.tw.

52. post-operative.tw.

53. postoperative.tw.

54. post$surgery.tw.

55. (analgesic adj trial).tw.

56. oral$.tw.

57. acupuncture.tw.

58. acupressure.tw.

59. scar.tw.

60. infection.tw.

For peer review only

61. dental.tw.

62. post$surgical.tw.

63. pre$surgical.tw.

64. case report.tw.

65. case study.tw.

66. pacing.tw.

67. stimulation.tw.

68. growth factor$.tw.

69. hormon$.tw.

70. or/24-31

71. or/32-69

72. 17 and 23

73. 72 and 70

74. 73 not 71

Ovid EMBASE

1. Clinical trial/

2. Randomized controlled trial/

3. Randomization/

4. Single blind procedure/

5. Double blind procedure/

6. Crossover procedure/

7. Randomi?ed controlled trial$.tw.

8. Rct.tw.

9. random allocation.tw.

10. Randomly allocated.tw.

11. Allocated randomly.tw.

12. (allocated adj2 random).tw.

13. Single blind$.tw.

14. Single blind$.tw.

15. or/1-14

16. Placebo$.tw.

For peer review only

17. placebo effect$.tw.

18. sham.tw.

19. placebo.tw.

20. or/16-19

21. surgery.tw.

22. surgical.tw.

23. arthroscopy.tw.

24. endoscopy.tw.

25. $scopy.tw.

26. $scopic.tw.

27. laparoscopy.tw.

28. transplantation.tw.

29. or/21-28

30. letter/

31. Review/

32. animal/

33. editorial/

34. ((meta adj analy$) or metaanalys$).tw.

35. (analgesic adj trial).tw.

36. meta$analysis.tw.

37. dose$.tw.

38. oral$.tw.

39. orally.tw.

40. dental.tw.

41. pre$medication.tw.

42. pre$surgical.tw.

43. post$surgical.tw.

44. pre$surgery.tw.

45. post$surgery.tw.

46. antibiotic$.tw.

47. an$esthetic$.tw.

For peer review only

48. steroid$.tw.

49. peri$operative.tw.

50. pre$emptive.tw.

51. preanesthetic$.tw.

52. post$operative.tw.

53. prophylaxis.tw.

54. prevention.tw.

55. acupuncture.tw.

56. accupressure.tw.

57. scar$.tw.

58. infection$.tw.

59. acupressure.tw.

60. pre$operative.tw.

61. growth factor$.tw.

62. pacing.tw.

63. stimulation.tw.

64. hormon$.tw.

65. case report$.tw.

66. case study.tw.

67. or/30-66

68. 15 and 20

69. 68 and 29

70. 69 not 67

Cochrane Central Register of Controlled Trials

http://onlinelibrary.wiley.com/o/cochrane/cochrane_clcentral_articles_fs.html

(placebo OR placebo effect OR sham OR imitation):ti,ab,kw and (surgery OR surgical OR laparoscopy OR endoscopy

OR arthroscopy OR transplantation OR scopy):ti,ab,kw and (clinical trial OR randomised clinical trail OR RCT OR

randomised controlled trial OR randomisation):ti,ab,kw not (drug OR dental OR oral OR infection OR steroids OR

For peer review only

hormones OR growth factor OR prophylaxis OR anaesthesia OR pre-surgical OR post-surgical OR pre-emptive OR

post-operative OR preoperative OR antibiotics OR acupuncture OR acupressure OR scar OR infection OR

prevention):ti,ab,kw not (review OR animal OR stimulation):ti,ab,kw in Trials

For peer review only

Supplementary table 2. List of references of 131 included RCTs

Trial #	References (alphabetical order)
1	Abbott, J., Hawe, J., Hunter, D., Holmes, M., Finn, P., & Garry, R. (2004). Laparoscopic excision of endometriosis: a randomized, placebo-controlled trial. Fertility and sterility, 82(4), 878–884. https://doi.org/10.1016/j.fertnstert.2004.03.046
2	Al-Lamee, R., Thompson, D., Dehbi, H. M., Sen, S., Tang, K., Davies, J., Keeble, T., Mielewczik, M., Kaprielian, R., Malik, I. S., Nijjer, S. S., Petraco, R., Cook, C., Ahmad, Y., Howard, J., Baker, C., Sharp, A., Gerber, R., Talwar, S., Assomull, R., … ORBITA investigators (2018). Percutaneous coronary intervention in stable angina (ORBITA): a double-blind, randomised controlled trial. Lancet (London, England), 391(10115), 31–40. https://doi.org/10.1016/S0140-6736(17)32714-9
3	Anderson, D. B., Ferreira, M. L., Harris, I. A., Davis, G. A., Stanford, R., Beard, D., Li, Q., Jan, S., Mobbs, R. J., Maher, C. G., Yong, R., Zammit, T., Latimer, J., & Buchbinder, R. (2019). SUcceSS, SUrgery for Spinal Stenosis: protocol of a randomised, placebo-controlled trial. BMJ open, 9(2), e024944. https://doi.org/10.1136/bmjopen-2018-024944
4	Arts, J., Bisschops, R., Blondeau, K., Farré, R., Vos, R., Holvoet, L., Caenepeel, P., Lerut, A., & Tack, J. (2012). A double-blind sham-controlled study of the effect of radiofrequency energy on symptoms and distensibility of the gastro-esophageal junction in GERD. The American journal of gastroenterology, 107(2), 222–230. https://doi.org/10.1038/ajg.2011.395
5	Bäck, L. J., Liukko, T., Rantanen, I., Peltola, J. S., Partinen, M., Ylikoski, J., & Mäkitie, A. A. (2009). Radiofrequency surgery of the soft palate in the treatment of mild obstructive sleep apnea is not effective as a single-stage procedure: A randomized single-blinded placebo-controlled trial. The Laryngoscope, 119(8), 1621–1627. https://doi.org/10.1002/lary.20562
6	Bajbouj, M., Becker, V., Eckel, F., Miehlke, S., Pech, O., Prinz, C., Schmid, R. M., & Meining, A. (2009). Argon plasma coagulation of cervical heterotopic gastric mucosa as an alternative treatment for globus sensations. Gastroenterology, 137(2), 440–444. https://doi.org/10.1053/j.gastro.2009.04.053
7	Beard, D. J., Rees, J. L., Cook, J. A., Rombach, I., Cooper, C., Merritt, N., Shirkey, B. A., Donovan, J. L., Gwilym, S., Savulescu, J., Moser, J., Gray, A., Jepson, M., Tracey, I., Judge, A., Wartolowska, K., Carr, A. J., & CSAW Study Group (2018). Arthroscopic subacromial decompression for subacromial shoulder pain (CSAW): a multicentre, pragmatic, parallel group, placebo-controlled, three-group, randomised surgical trial. Lancet (London, England), 391(10118), 329–338. https://doi.org/10.1016/S0140-6736(17)32457-1
8	Benjamin, S. B., Maher, K. A., Cattau, E. L., Jr, Collen, M. J., Fleischer, D. E., Lewis, J. H., Ciarleglio, C. A., Earll, J. M., Schaffer, S., & Mirkin, K. (1988). Double-blind controlled trial of the Garren-Edwards gastric bubble: an adjunctive treatment for exogenous obesity. Gastroenterology, 95(3), 581–588. https://doi.org/10.1016/s0016-5085(88)80001-5
9	Blaganje, M., Šćepanović, D., Žgur, L., Verdenik, I., Pajk, F., & Lukanović, A. (2018). Non-ablative Er:YAG laser therapy effect on stress urinary incontinence related to quality of life and sexual function: A randomized controlled trial. European journal of obstetrics, gynecology, and reproductive biology, 224, 153–158. https://doi.org/10.1016/j.ejogrb.2018.03.038
10	Böhm, M., Kario, K., Kandzari, D. E., Mahfoud, F., Weber, M. A., Schmieder, R. E., Tsioufis, K., Pocock, S., Konstantinidis, D., Choi, J. W., East, C., Lee, D. P., Ma, A., Ewen, S., Cohen, D. L., Wilensky, R., Devireddy, C. M., Lea, J., Schmid, A., Weil, J., … SPYRAL HTN-OFF MED Pivotal Investigators (2020). Efficacy of catheter-based renal denervation in the absence of antihypertensive medications (SPYRAL HTN-OFF MED Pivotal): a multicentre, randomised, sham-controlled trial. Lancet (London, England), 395(10234), 1444–1451. https://doi.org/10.1016/S0140-6736(20)30554-7

For peer review only

11	Bradley, J. D., Heilman, D. K., Katz, B. P., Gsell, P., Wallick, J. E., & Brandt, K. D. (2002). Tidal irrigation as treatment for knee osteoarthritis: a sham-controlled, randomized, double-blinded evaluation. Arthritis and rheumatism, 46(1), 100–108. https://doi.org/10.1002/1529-0131(200201)46:1<100::aid-art10037>3.0.co;2-v
12	Buchbinder, R., Osborne, R. H., Ebeling, P. R., Wark, J. D., Mitchell, P., Wriedt, C., Graves, S., Staples, M. P., & Murphy, B. (2009). A randomized trial of vertebroplasty for painful osteoporotic vertebral fractures. The New England journal of medicine, 361(6), 557–568. https://doi.org/10.1056/NEJMoa0900429
13	Campbell, M. K., Entwistle, V. A., Cuthbertson, B. H., Skea, Z. C., Sutherland, A. G., McDonald, A. M., Norrie, J. D., Carlson, R. V., Bridgman, S., & KORAL study group (2011). Developing a placebo-controlled trial in surgery: issues of design, acceptability and feasibility. Trials, 12, 50. https://doi.org/10.1186/1745-6215-12-50
14	Castro, M., Rubin, A. S., Laviolette, M., Fiterman, J., De Andrade Lima, M., Shah, P. L., Fiss, E., Olivenstein, R., Thomson, N. C., Niven, R. M., Pavord, I. D., Simoff, M., Duhamel, D. R., McEvoy, C., Barbers, R., Ten Hacken, N. H., Wechsler, M. E., Holmes, M., Phillips, M. J., Erzurum, S., … AIR2 Trial Study Group (2010). Effectiveness and safety of bronchial thermoplasty in the treatment of severe asthma: a multicenter, randomized, double-blind, sham-controlled clinical trial. American journal of respiratory and critical care medicine, 181(2), 116–124. https://doi.org/10.1164/rccm.200903-0354OC
15	Chan, D. L., Cruz, J. R., Mui, W. L., Wong, S. K. H., & Ng, E. K. W. (2021). Outcomes with Intra-gastric Balloon Therapy in BMI < 35 Non-morbid Obesity: 10-Year Follow-Up Study of an RCT. Obesity surgery, 31(2), 781–786. https://doi.org/10.1007/s11695-020-04986-3
16	Clark, W., Bird, P., Gonski, P., Diamond, T. H., Smerdely, P., McNeil, H. P., Schlaphoff, G., Bryant, C., Barnes, E., & Gebski, V. (2016). Safety and efficacy of vertebroplasty for acute painful osteoporotic fractures (VAPOUR): a multicentre, randomised, double-blind, placebo-controlled trial. Lancet (London, England), 388(10052), 1408–1416. https://doi.org/10.1016/S0140-6736(16)31341-1
17	Cobb, L. A., Thomas, G. I., Dillard, D. H., Merendino, K. A., & Bruce, R. A. (1959). An evaluation of internal-mammary-artery ligation by a double-blind technic. The New England journal of medicine, 260(22), 1115–1118. https://doi.org/10.1056/NEJM195905282602204
18	Corley, D. A., Katz, P., Wo, J. M., Stefan, A., Patti, M., Rothstein, R., Edmundowicz, S., Kline, M., Mason, R., & Wolfe, M. M. (2003). Improvement of gastroesophageal reflux symptoms after radiofrequency energy: a randomized, sham-controlled trial. Gastroenterology, 125(3), 668–676. https://doi.org/10.1016/s0016-5085(03)01052-7
19	Cotton, P. B., Durkalski, V., Romagnuolo, J., Pauls, Q., Fogel, E., Tarnasky, P., Aliperti, G., Freeman, M., Kozarek, R., Jamidar, P., Wilcox, M., Serrano, J., Brawman-Mintzer, O., Elta, G., Mauldin, P., Thornhill, A., Hawes, R., Wood-Williams, A., Orrell, K., Drossman, D., … Robuck, P. (2014). Effect of endoscopic sphincterotomy for suspected sphincter of Oddi dysfunction on pain-related disability following cholecystectomy: the EPISOD randomized clinical trial. JAMA, 311(20), 2101–2109. https://doi.org/10.1001/jama.2014.5220
20	Davey, C., Zoumot, Z., Jordan, S., McNulty, W. H., Carr, D. H., Hind, M. D., Hansell, D. M., Rubens, M. B., Banya, W., Polkey, M. I., Shah, P. L., & Hopkinson, N. S. (2015). Bronchoscopic lung volume reduction with endobronchial valves for patients with heterogeneous emphysema and intact interlobar fissures (the BeLieVeR-HIFi study): a randomised controlled trial. Lancet (London, England), 386(9998), 1066–1073. https://doi.org/10.1016/S0140-6736(15)60001-0
21	Davys, H. J., Turner, D. E., Helliwell, P. S., Conaghan, P. G., Emery, P., & Woodburn, J. (2005). Debridement of plantar callosities in rheumatoid arthritis: a randomized controlled trial. Rheumatology (Oxford, England), 44(2), 207–210. https://doi.org/10.1093/rheumatology/keh435
22	Dawes, P. T., Kirlew, C., & Haslock, I. (1987). Saline washout for knee osteoarthritis: results of a controlled study. Clinical rheumatology, 6(1), 61–63. https://doi.org/10.1007/BF02201002

For peer review only

23	de Quadros, L. G., Neto, M. G., Marchesini, J. C., Teixeira, A., Grecco, E., Junior, R. L. K., Zundel, N., Filho, I. J. Z., de Souza, T. F., Filho, A. C., da Silva, L. B., Ramos, A. C., Ferraz, Á. A. B., & Campos, J. M. (2020). Endoscopic Argon Plasma Coagulation vs. Multidisciplinary Evaluation in the Management of Weight Regain After Gastric Bypass Surgery: a Randomized Controlled Trial with SHAM Group. Obesity surgery, 30(5), 1904–1916. https://doi.org/10.1007/s11695-020-04414-6
24	Devière, J., Costamagna, G., Neuhaus, H., Voderholzer, W., Louis, H., Tringali, A., Marchese, M., Fiedler, T., Darb-Esfahani, P., & Schumacher, B. (2005). Nonresorbable copolymer implantation for gastroesophageal reflux disease: a randomized sham-controlled multicenter trial. Gastroenterology, 128(3), 532–540. https://doi.org/10.1053/j.gastro.2004.12.005
25	Dimond, E. G., Kittle, C. F., & Crockett, J. E. (1960). Comparison of internal mammary artery ligation and sham operation for angina pectoris. The American journal of cardiology, 5, 483–486. https://doi.org/10.1016/0002-9149(60)90105-3
26	Donnenfeld, E. D., Holland, E. J., & Solomon, K. D. (2021). Safety and efficacy of nepafenac punctal plug delivery system in controlling postoperative ocular pain and inflammation after cataract surgery. Journal of cataract and refractive surgery, 47(2), 158–164. https://doi.org/10.1097/j.jcrs.0000000000000414
27	Dowson, A., Mullen, M. J., Peatfield, R., Muir, K., Khan, A. A., Wells, C., Lipscombe, S. L., Rees, T., De Giovanni, J. V., Morrison, W. L., Hildick-Smith, D., Elrington, G., Hillis, W. S., Malik, I. S., & Rickards, A. (2008). Migraine Intervention With STARFlex Technology (MIST) trial: a prospective, multicenter, double-blind, sham-controlled trial to evaluate the effectiveness of patent foramen ovale closure with STARFlex septal repair implant to resolve refractory migraine headache. Circulation, 117(11), 1397–1404. https://doi.org/10.1161/CIRCULATIONAHA.107.727271
28	Eid, G. M., McCloskey, C. A., Eagleton, J. K., Lee, L. B., & Courcoulas, A. P. (2014). StomaphyX vs a sham procedure for revisional surgery to reduce regained weight in Roux-en-Y gastric bypass patients: a randomized clinical trial. JAMA surgery, 149(4), 372–379. https://doi.org/10.1001/jamasurg.2013.4051
29	Engholm, M., Bertelsen, J. B., Mathiassen, O. N., Bøtker, H. E., Vase, H., Peters, C. D., Bech, J. N., Buus, N. H., Schroeder, A. P., Rickers, H., Hansen, K. W., Poulsen, P. L., Kaltoft, A., & Christensen, K. L. (2018). Effects of renal denervation on coronary flow reserve and forearm dilation capacity in patients with treatment-resistant hypertension. A randomized, double-blinded, sham-controlled clinical trial. International journal of cardiology, 250, 29–34. https://doi.org/10.1016/j.ijcard.2017.09.200
30	Feldman, T., Mauri, L., Kahwash, R., Litwin, S., Ricciardi, M. J., van der Harst, P., Penicka, M., Fail, P. S., Kaye, D. M., Petrie, M. C., Basuray, A., Hummel, S. L., Forde-McLean, R., Nielsen, C. D., Lilly, S., Massaro, J. M., Burkhoff, D., Shah, S. J., & REDUCE LAP-HF I Investigators and Study Coordinators (2018). Transcatheter Interatrial Shunt Device for the Treatment of Heart Failure With Preserved Ejection Fraction (REDUCE LAP-HF I [Reduce Elevated Left Atrial Pressure in Patients With Heart Failure]): A Phase 2, Randomized, Sham-Controlled Trial. Circulation, 137(4), 364–375. https://doi.org/10.1161/CIRCULATIONAHA.117.032094
31	Firanescu, C. E., de Vries, J., Lodder, P., Venmans, A., Schoemaker, M. C., Smeets, A. J., Donga, E., Juttmann, J. R., Klazen, C. A. H., Elgersma, O. E. H., Jansen, F. H., Tielbeek, A. V., Boukrab, I., Schonenberg, K., van Rooij, W. J. J., Hirsch, J. A., & Lohle, P. N. M. (2018). Vertebroplasty versus sham procedure for painful acute osteoporotic vertebral compression fractures (VERTOS IV): randomised sham controlled clinical trial. BMJ (Clinical research ed.), 361, k1551. https://doi.org/10.1136/bmj.k1551<br><br>Firanescu, C. E., de Vries, J., Lodder, P., Schoemaker, M. C., Smeets, A. J., Donga, E., Juttmann, J. R., Klazen, C. A. H., Elgersma, O. E. H., Jansen, F. H., van der Horst, I., Blonk, M., Venmans, A., & Lohle, P. N. M. (2019). Percutaneous Vertebroplasty is no Risk Factor for New Vertebral Fractures and Protects Against Further Height Loss (VERTOS IV). Cardiovascular and interventional radiology, 42(7), 991–1000. https://doi.org/10.1007/s00270-019-02205-w

32	Fischgrund, J. S., Rhyne, A., Franke, J., Sasso, R., Kitchel, S., Bae, H., Yeung, C., Truumees, E., Schaufele, M., Yuan, P., Vajkoczy, P., DePalma, M., Anderson, D. G., Thibodeau, L., & Meyer, B. (2018). Intraosseous basivertebral nerve ablation for the treatment of chronic low back pain: a prospective randomized double-blind sham-controlled multi-center study. European spine journal: official publication of the European Spine Society, the European Spinal Deformity Society, and the European Section of the Cervical Spine Research Society, 27(5), 1146–1156. https://doi.org/10.1007/s00586-018-5496-1
33	Fleischer D. (1985). Endoscopic Nd:YAG laser therapy for active esophageal variceal bleeding. A randomized controlled study. Gastrointestinal endoscopy, 31(1), 4–9. https://doi.org/10.1016/s0016-5107(85)71954-2
34	Fockens, P., Cohen, L., Edmundowicz, S. A., Binmoeller, K., Rothstein, R. I., Smith, D., Lin, E., Nickl, N., Overholt, B., Kahrilas, P. J., Vakil, N., Abdel Aziz Hassan, A. M., & Lehman, G. A. (2010). Prospective randomized controlled trial of an injectable esophageal prosthesis versus a sham procedure for endoscopic treatment of gastroesophageal reflux disease. Surgical endoscopy, 24(6), 1387–1397. https://doi.org/10.1007/s00464-009-0784-9
35	Freed, C. R., Greene, P. E., Breeze, R. E., Tsai, W. Y., DuMouchel, W., Kao, R., Dillon, S., Winfield, H., Culver, S., Trojanowski, J. Q., Eidelberg, D., & Fahn, S. (2001). Transplantation of embryonic dopamine neurons for severe Parkinson's disease. The New England journal of medicine, 344(10), 710–719. https://doi.org/10.1056/NEJM200103083441002
36	Freeman, B. J., Fraser, R. D., Cain, C. M., Hall, D. J., & Chapple, D. C. (2005). A randomized, double-blind, controlled trial: intradiscal electrothermal therapy versus placebo for the treatment of chronic discogenic low back pain. Spine, 30(21), 2369–2378. https://doi.org/10.1097/01.brs.0000186587.43373.f2
37	Freitas, D., Donato, A., & Monteiro, J. G. (1985). Controlled trial of liquid monopolar electrocoagulation in bleeding peptic ulcers. The American journal of gastroenterology, 80(11), 853–857.
38	Friedman, M., Schalch, P., Lin, H. C., Kakodkar, K. A., Joseph, N. J., & Mazloom, N. (2008). Palatal implants for the treatment of snoring and obstructive sleep apnea/hypopnea syndrome. Otolaryngology--head and neck surgery : official journal of American Academy of Otolaryngology-Head and Neck Surgery, 138(2), 209–216. https://doi.org/10.1016/j.otohns.2007.10.026
39	Fullarton, G. M., Birnie, G. G., Macdonald, A., & Murray, W. R. (1989). Controlled trial of heater probe treatment in bleeding peptic ulcers. The British journal of surgery, 76(6), 541–544. https://doi.org/10.1002/bjs.1800760606
40	Garcia, C. A., Soler, F. C. (2018). The Effect of Plantar Hyperkeratosis Debridement on Self-Perception of Pain Levels in older People. International journal of gerontology, 12(4), 314–318. https://doi.org/10.1016/j.ijge.2018.05.002
41	Geenen, J. E., Hogan, W. J., Dodds, W. J., Toouli, J., & Venu, R. P. (1989). The efficacy of endoscopic sphincterotomy after cholecystectomy in patients with sphincter-of-Oddi dysfunction. The New England journal of medicine, 320(2), 82–87. https://doi.org/10.1056/NEJM198901123200203
42	Geliebter, A., Melton, P. M., Gage, D., McCray, R. S., & Hashim, S. A. (1990). Gastric balloon to treat obesity: a double-blind study in nondieting subjects. The American journal of clinical nutrition, 51(4), 584–588. https://doi.org/10.1093/ajcn/51.4.584
43	Genco, A., Cipriano, M., Bacci, V., Cuzzolaro, M., Materia, A., Raparelli, L., Docimo, C., Lorenzo, M., & Basso, N. (2006). BioEnterics Intragastric Balloon (BIB): a short-term, double-blind, randomised, controlled, crossover study on weight reduction in morbidly obese patients. International journal of obesity (2005), 30(1), 129–133. https://doi.org/10.1038/sj.ijo.0803094
44	Gillespie, M. B., Wylie, P. E., Lee-Chiong, T., & Rapoport, D. M. (2011). Effect of palatal implants on continuous positive airway pressure and compliance. Otolaryngology--head and neck surgery: official journal of American Academy of Otolaryngology-Head and Neck Surgery, 144(2), 230–236. https://doi.org/10.1177/0194599810392173

For peer review only

45	Gross, R. E., Watts, R. L., Hauser, R. A., Bakay, R. A., Reichmann, H., von Kummer, R., Ondo, W. G., Reissig, E., Eisner, W., Steiner-Schulze, H., Siedentop, H., Fichte, K., Hong, W., Cornfeldt, M., Beebe, K., Sandbrink, R., & Spheramine Investigational Group (2011). Intrastriatal transplantation of microcarrier-bound human retinal pigment epithelial cells versus sham surgery in patients with advanced Parkinson's disease: a double-blind, randomised, controlled trial. The Lancet. Neurology, 10(6), 509–519. https://doi.org/10.1016/S1474-4422(11)70097-7
46	Guymer, R. H., Wu, Z., Hodgson, L. A. B., Caruso, E., Brassington, K. H., Tindill, N., Aung, K. Z., McGuinness, M. B., Fletcher, E. L., Chen, F. K., Chakravarthy, U., Arnold, J. J., Heriot, W. J., Durkin, S. R., Lek, J. J., Harper, C. A., Wickremasinghe, S. S., Sandhu, S. S., Baglin, E. K., Sharangan, P., … Laser Intervention in Early Stages of Age-Related Macular Degeneration Study Group (2019). Subthreshold Nanosecond Laser Intervention in Age-Related Macular Degeneration: The LEAD Randomized Controlled Clinical Trial. Ophthalmology, 126(6), 829–838. https://doi.org/10.1016/j.ophtha.2018.09.015
47	Guyuron, B., Reed, D., Kriegler, J. S., Davis, J., Pashmini, N., & Amini, S. (2009). A placebo-controlled surgical trial of the treatment of migraine headaches. Plastic and reconstructive surgery, 124(2), 461–468. https://doi.org/10.1097/PRS.0b013e3181adcf6a
48	Håkansson, B., Montgomery, M., Cadiere, G. B., Rajan, A., Bruley des Varannes, S., Lerhun, M., Coron, E., Tack, J., Bischops, R., Thorell, A., Arnelo, U., & Lundell, L. (2015). Randomised clinical trial: transoral incisionless fundoplication vs. sham intervention to control chronic GERD. Alimentary pharmacology & therapeutics, 42(11-12), 1261–1270. https://doi.org/10.1111/apt.13427
49	Hansen, E.J., Simony, A., Carreon, L., Rousing, R., Tropp, H., Andersen, M.O. (2019). Vertebroplasty vs. SHAM for Treating Osteoporotic Vertebral Compression Fractures: A Double Blind RCT. Integrative journal of orthopaedics and traumatology, 2(4), 1–6. https://doi.org/10.31038/IJOT.2019244
50	Harju, T., Kivekäs, I., Numminen, J., & Rautiainen, M. (2018). The effect of inferior turbinate surgery on ear symptoms. The Laryngoscope, 128(3), 568–572. https://doi.org/10.1002/lary.26823
51	Harju, T., Numminen, J., Kivekäs, I., & Rautiainen, M. (2018). A prospective, randomized, placebo-controlled study of inferior turbinate surgery. The Laryngoscope, 128(9), 1997–2003. https://doi.org/10.1002/lary.27103<br><br>Kankaanpää, A., Harju, T., & Numminen, J. (2021). The Effect of Inferior Turbinate Surgery on Quality of Life: A Randomized, Placebo-Controlled Study. Ear, nose, & throat journal, 100(10_suppl), 1107S–1112S. https://doi.org/10.1177/0145561320927944
52	Hartigan P. (1994). Sclerotherapy for male alcoholic cirrhotic patients who have bled from esophageal varices: results of a randomized, multicenter clinical trial. Hepatology, 20(3), 618–625. https://doi.org/10.1002/hep.1840200311
53	Heidari, M., Paknejad, M., Jamali, R., Nokhbatolfoghahaei, H., Fekrazad, R., & Moslemi, N. (2017). Effect of laser photobiomodulation on wound healing and postoperative pain following free gingival graft: A split-mouth triple-blind randomized controlled clinical trial. Journal of photochemistry and photobiology. B, Biology, 172, 109–114. https://doi.org/10.1016/j.jphotobiol.2017.05.022
54	Hersh, P. S., Stulting, R. D., Muller, D., Durrie, D. S., Rajpal, R. K., & U.S. Crosslinking Study Group (2017). U.S. Multicenter Clinical Trial of Corneal Collagen Crosslinking for Treatment of Corneal Ectasia after Refractive Surgery. Ophthalmology, 124(10), 1475–1484. https://doi.org/10.1016/j.ophtha.2017.05.036
55	Hogan, R. B., Johnston, J. H., Long, B. W., Sones, J. Q., Hinton, L. A., Bunge, J., & Corrigan, S. A. (1989). A double-blind, randomized, sham-controlled trial of the gastric bubble for obesity. Gastrointestinal endoscopy, 35(5), 381–385. https://doi.org/10.1016/s0016-5107(89)72839-x

56	Hollenbach, M., Prettin, C., Gundling, F., Schepp, W., Seufert, J., Stein, J., Rösch, T., Aberle, J., Feisthammel, J., Petroff, D., & Hoffmeister, A. (2018). Design of the Weight-loss Endoscopy Trial (WET): a multi-center, randomized, controlled trial comparing weight loss in endoscopically implanted duodenal-jejunal bypass liners vs. intragastric balloons vs. a sham procedure. BMC gastroenterology, 18(1), 118. https://doi.org/10.1186/s12876-018-0838-3
57	Holmlund, T., Levring-Jäghagen, E., Franklin, K. A., Lindkvist, M., & Berggren, D. (2014). Effects of Radiofrequency versus sham surgery of the soft palate on daytime sleepiness. The Laryngoscope, 124(10), 2422–2426. https://doi.org/10.1002/lary.24580
58	Hunter, J. G., Kahrilas, P. J., Bell, R. C., Wilson, E. B., Trad, K. S., Dolan, J. P., Perry, K. A., Oelschlager, B. K., Soper, N. J., Snyder, B. E., Burch, M. A., Melvin, W. S., Reavis, K. M., Turgeon, D. G., Hungness, E. S., & Diggs, B. S. (2015). Efficacy of transoral fundoplication vs omeprazole for treatment of regurgitation in a randomized controlled trial. Gastroenterology, 148(2), 324–333.e5. https://doi.org/10.1053/j.gastro.2014.10.009
59	Ikramuddin, S., Blackstone, R. P., Brancatisano, A., Toouli, J., Shah, S. N., Wolfe, B. M., Fujioka, K., Maher, J. W., Swain, J., Que, F. G., Morton, J. M., Leslie, D. B., Brancatisano, R., Kow, L., O'Rourke, R. W., Deveney, C., Takata, M., Miller, C. J., Knudson, M. B., Tweden, K. S., … Billington, C. J. (2014). Effect of reversible intermittent intra-abdominal vagal nerve blockade on morbid obesity: the ReCharge randomized clinical trial. JAMA, 312(9), 915–922. https://doi.org/10.1001/jama.2014.10540
60	Jarrell, J., Mohindra, R., Ross, S., Taenzer, P., & Brant, R. (2005). Laparoscopy and reported pain among patients with endometriosis. Journal of obstetrics and gynaecology Canada : JOGC = Journal d'obstetrique et gynecologie du Canada : JOGC, 27(5), 477–485. https://doi.org/10.1016/s1701-2163(16)30531-x
61	Kallmes, D. F., Comstock, B. A., Heagerty, P. J., Turner, J. A., Wilson, D. J., Diamond, T. H., Edwards, R., Gray, L. A., Stout, L., Owen, S., Hollingworth, W., Ghdoke, B., Annesley-Williams, D. J., Ralston, S. H., & Jarvik, J. G. (2009). A randomized trial of vertebroplasty for osteoporotic spinal fractures. The New England journal of medicine, 361(6), 569–579. https://doi.org/10.1056/NEJMoa0900563
62	Kalunian, K. C., Moreland, L. W., Klashman, D. J., Brion, P. H., Concoff, A. L., Myers, S., Singh, R., Ike, R. W., Seeger, L. L., Rich, E., & Skovron, M. L. (2000). Visually-guided irrigation in patients with early knee osteoarthritis: a multicenter randomized, controlled trial. Osteoarthritis and cartilage, 8(6), 412–418. https://doi.org/10.1053/joca.1999.0316
63	Kaminski, R., Kulinski, K., Kozar-Kaminska, K., Wasko, M. K., Langner, M., & Pomianowski, S. (2019). Repair Augmentation of Unstable, Complete Vertical Meniscal Tears With Bone Marrow Venting Procedure: A Prospective, Randomized, Double-Blind, Parallel-Group, Placebo-Controlled Study. Arthroscopy: the journal of arthroscopic & related surgery: official publication of the Arthroscopy Association of North America and the International Arthroscopy Association, 35(5), 1500–1508.e1. https://doi.org/10.1016/j.arthro.2018.11.056
64	Kang, J., Lee, G., Kim, J., Kim, Y., Park, S., & Lee, D. (2020). Effects and safety of intranasal phototherapy for allergic rhinitis: Study protocol for a single-center, randomized, double-blind, parallel, placebo-controlled, investigator-initiated, pilot study. Medicine, 99(30), e20835. https://doi.org/10.1097/MD.0000000000020835
65	Kang, T., Sung, C. M., & Yang, H. C. (2019). Radiofrequency ablation of turbinates after septoplasty has no effect on allergic rhinitis symptoms other than nasal obstruction. International forum of allergy & rhinology, 9(11), 1257–1262. https://doi.org/10.1002/alr.22420
66	Kern, R. C., Stolovitzky, J. P., Silvers, S. L., Singh, A., Lee, J. T., Yen, D. M., Iloreta, A. M. C., Jr, Langford, F. P. J., Karanfilov, B., Matheny, K. E., Stambaugh, J. W., Gawlicka, A. K., & RESOLVE II study investigators (2018). A phase 3 trial of mometasone furoate sinus implants for chronic sinusitis with recurrent nasal polyps. International forum of allergy & rhinology, 8(4), 471–481. https://doi.org/10.1002/alr.22084

67	Kernohan, R. M., Anderson, J. R., McKelvey, S. T., & Kennedy, T. L. (1984). A controlled trial of bipolar electrocoagulation in patients with upper gastrointestinal bleeding. The British journal of surgery, 71(11), 889–891. https://doi.org/10.1002/bjs.1800711128
68	Koutsourelakis, I., Georgoulopoulos, G., Perraki, E., Vagiakis, E., Roussos, C., & Zakynthinos, S. G. (2008). Randomised trial of nasal surgery for fixed nasal obstruction in obstructive sleep apnoea. The European respiratory journal, 31(1), 110–117. https://doi.org/10.1183/09031936.00087607
69	Krejs, G. J., Little, K. H., Westergaard, H., Hamilton, J. K., Spady, D. K., & Polter, D. E. (1987). Laser photocoagulation for the treatment of acute peptic-ulcer bleeding. A randomized controlled clinical trial. The New England journal of medicine, 316(26), 1618–1621. https://doi.org/10.1056/NEJM198706253162602
70	Kroslak, M., & Murrell, G. A. C. (2018). Surgical Treatment of Lateral Epicondylitis: A Prospective, Randomized, Double-Blinded, Placebo-Controlled Clinical Trial. The American journal of sports medicine, 46(5), 1106–1113. https://doi.org/10.1177/0363546517753385
71	Laine L. (1987). Multipolar electrocoagulation in the treatment of active upper gastrointestinal tract hemorrhage. A prospective controlled trial. The New England journal of medicine, 316(26), 1613–1617. https://doi.org/10.1056/NEJM198706253162601
72	Landers, S., Hely, A., Harrison, B., Maister, N., Hely, R., Lane, S. E., Gill, S. D., & Page, R. S. (2017). Protocol for a single-centre, parallel-arm, randomised controlled superiority trial evaluating the effects of transcatheter arterial embolisation of abnormal knee neovasculature on pain, function and quality of life in people with knee osteoarthritis. BMJ open, 7(5), e014266. https://doi.org/10.1136/bmjopen-2016-014266
73	Larrosa, F., Hernandez, L., Morello, A., Ballester, E., Quinto, L., & Montserrat, J. M. (2004). Laser-assisted uvulopalatoplasty for snoring: does it meet the expectations?. The European respiratory journal, 24(1), 66–70. https://doi.org/10.1183/09031936.04.00082903
74	Lee, P. E., Kung, R. C., & Drutz, H. P. (2001). Periurethral autologous fat injection as treatment for female stress urinary incontinence: a randomized double-blind controlled trial. The Journal of urology, 165(1), 153–158. https://doi.org/10.1097/00005392-200101000-00037
75	Leon, M. B., Kornowski, R., Downey, W. E., Weisz, G., Baim, D. S., Bonow, R. O., Hendel, R. C., Cohen, D. J., Gervino, E., Laham, R., Lembo, N. J., Moses, J. W., & Kuntz, R. E. (2005). A blinded, randomized, placebo-controlled trial of percutaneous laser myocardial revascularization to improve angina symptoms in patients with severe coronary disease. Journal of the American College of Cardiology, 46(10), 1812–1819. https://doi.org/10.1016/j.jacc.2005.06.079
76	Lindor, K. D., Hughes, R. W., Jr, Ilstrup, D. M., & Jensen, M. D. (1987). Intragastric balloons in comparison with standard therapy for obesity--a randomized, double-blind trial. Mayo Clinic proceedings, 62(11), 992–996. https://doi.org/10.1016/s0025-6196(12)65069-1
77	MacLeod, I. A., Mills, P. R., MacKenzie, J. F., Joffe, S. N., Russell, R. I., & Carter, D. C. (1983). Neodymium yttrium aluminium garnet laser photocoagulation for major haemorrhage from peptic ulcers and single vessels: a single blind controlled study. British medical journal (Clinical research ed.), 286(6362), 345–348. https://doi.org/10.1136/bmj.286.6362.345
78	Matache, B. A., Berdusco, R., Momoli, F., Lapner, P. L., & Pollock, J. W. (2016). A randomized, double-blind sham-controlled trial on the efficacy of arthroscopic tennis elbow release for the management of chronic lateral epicondylitis. BMC musculoskeletal disorders, 17, 239. https://doi.org/10.1186/s12891-016-1093-9
79	Mathus-Vliegen, E. M., Tytgat, G. N., & Veldhuyzen-Offermans, E. A. (1990). Intragastric balloon in the treatment of super-morbid obesity. Double-blind, sham-controlled, crossover evaluation of 500-milliliter balloon. Gastroenterology, 99(2), 362–369. https://doi.org/10.1016/0016-5085(90)91017-z
80	Maurer, J. T., Sommer, J. U., Hein, G., Hörmann, K., Heiser, C., & Stuck, B. A. (2012). Palatal implants in the treatment of obstructive sleep apnea: a randomised, placebo-controlled single-centre trial. European archives of oto-rhino-laryngology: official journal of the European Federation of Oto-Rhino-Laryngological Societies (EUFOS): affiliated with the German Society for Oto-Rhino-Laryngology - Head and Neck Surgery, 269(7), 1851–1856. https://doi.org/10.1007/s00405-011-1920-4

For peer review only - http://bmjopen.bmj.com/site/about/guidelines.xhtml

81	Mehta, V., Poply, K., Husband, M., Anwar, S., & Langford, R. (2018). The Effects of Radiofrequency Neurotomy Using a Strip-Lesioning Device on Patients with Sacroiliac Joint Pain: Results from a Single-Center, Randomized, Sham-Controlled Trial. Pain physician, 21(6), 607–618. (No DOI - https://pubmed.ncbi.nlm.nih.gov/30508988/)
82	Meshkinpour, H., Hsu, D., & Farivar, S. (1988). Effect of gastric bubble as a weight reduction device: a controlled, crossover study. Gastroenterology, 95(3), 589–592. https://doi.org/10.1016/s0016-5085(88)80002-7
83	Montgomery, M., Håkanson, B., Ljungqvist, O., Ahlman, B., & Thorell, A. (2006). Twelve months' follow-up after treatment with the EndoCinch endoscopic technique for gastro-oesophageal reflux disease: a randomized, placebo-controlled study. Scandinavian journal of gastroenterology, 41(12), 1382–1389. https://doi.org/10.1080/00365520600735738
84	Moseley, J. B., O'Malley, K., Petersen, N. J., Menke, T. J., Brody, B. A., Kuykendall, D. H., Hollingsworth, J. C., Ashton, C. M., & Wray, N. P. (2002). A controlled trial of arthroscopic surgery for osteoarthritis of the knee. The New England journal of medicine, 347(2), 81–88. https://doi.org/10.1056/NEJMoa013259
85	Nease, C. J., & Krempl, G. A. (2004). Radiofrequency treatment of turbinate hypertrophy: a randomized, blinded, placebo-controlled clinical trial. Otolaryngology--head and neck surgery: official journal of American Academy of Otolaryngology-Head and Neck Surgery, 130(3), 291–299. https://doi.org/10.1016/j.otohns.2003.11.003
86	Ninane, V., Geltner, C., Bezzi, M., Foccoli, P., Gottlieb, J., Welte, T., Seijo, L., Zulueta, J. J., Munavvar, M., Rosell, A., Lopez, M., Jones, P. W., Coxson, H. O., Springmeyer, S. C., & Gonzalez, X. (2012). Multicentre European study for the treatment of advanced emphysema with bronchial valves. The European respiratory journal, 39(6), 1319–1325. https://doi.org/10.1183/09031936.00019711
87	O'Brien, J. D., Day, S. J., & Burnham, W. R. (1986). Controlled trial of small bipolar probe in bleeding peptic ulcers. Lancet (London, England), 1(8479), 464–467. https://doi.org/10.1016/s0140-6736(86)92928-4
88	Olanow, C. W., Goetz, C. G., Kordower, J. H., Stoessl, A. J., Sossi, V., Brin, M. F., Shannon, K. M., Nauert, G. M., Perl, D. P., Godbold, J., & Freeman, T. B. (2003). A double-blind controlled trial of bilateral fetal nigral transplantation in Parkinson's disease. Annals of neurology, 54(3), 403–414. https://doi.org/10.1002/ana.10720
89	Paavola, M., Malmivaara, A., Taimela, S., Kanto, K., Järvinen, T. L., & FIMPACT Investigators (2017). Finnish Subacromial Impingement Arthroscopy Controlled Trial (FIMPACT): a protocol for a randomised trial comparing arthroscopic subacromial decompression and diagnostic arthroscopy (placebo control), with an exercise therapy control, in the treatment of shoulder impingement syndrome. BMJ open, 7(5), e014087. https://doi.org/10.1136/bmjopen-2016-014087
90	Pauza, K. J., Howell, S., Dreyfuss, P., Peloza, J. H., Dawson, K., & Bogduk, N. (2004). A randomized, placebo-controlled trial of intradiscal electrothermal therapy for the treatment of discogenic low back pain. The spine journal: official journal of the North American Spine Society, 4(1), 27–35. https://doi.org/10.1016/j.spinee.2003.07.001
91	Ponce, J., Woodman, G., Swain, J., Wilson, E., English, W., Ikramuddin, S., Bour, E., Edmundowicz, S., Snyder, B., Soto, F., Sullivan, S., Holcomb, R., Lehmann, J., & REDUCE Pivotal Trial Investigators (2015). The REDUCE pivotal trial: a prospective, randomized controlled pivotal trial of a dual intragastric balloon for the treatment of obesity. Surgery for obesity and related diseases: official journal of the American Society for Bariatric Surgery, 11(4), 874–881. https://doi.org/10.1016/j.soard.2014.12.006
92	Powell, N. B., Zonato, A. I., Weaver, E. M., Li, K., Troell, R., Riley, R. W., & Guilleminault, C. (2001). Radiofrequency treatment of turbinate hypertrophy in subjects using continuous positive airway pressure: a randomized, double-blind, placebo-controlled clinical pilot trial. The Laryngoscope, 111(10), 1783–1790. https://doi.org/10.1097/00005537-200110000-00023

For peer review only

93	Ramhamadany, E. M., Fowler, J., & Baird, I. M. (1989). Effect of the gastric balloon versus sham procedure on weight loss in obese subjects. Gut, 30(8), 1054–1057. https://doi.org/10.1136/gut.30.8.1054
94	Risberg, M. A., Ageberg, E., Nilstad, A., Lund, B., Nordsletten, L., Løken, S., Ludvigsen, T., Kierkegaard, S., Carsen, S., Kostogiannis, I., Crossley, K. M., Glyn-Jones, S., & Kemp, J. L. (2018). Arthroscopic Surgical Procedures Versus Sham Surgery for Patients With Femoroacetabular Impingement and/or Labral Tears: Study Protocol for a Randomized Controlled Trial (HIPARTI) and a Prospective Cohort Study (HARP). The Journal of orthopaedic and sports physical therapy, 48(4), 325–335. https://doi.org/10.2519/jospt.2018.7931
95	Roehrborn, C. G., Gange, S. N., Shore, N. D., Giddens, J. L., Bolton, D. M., Cowan, B. E., Brown, B. T., McVary, K. T., Te, A. E., Gholami, S. S., Rashid, P., Moseley, W. G., Chin, P. T., Dowling, W. T., Freedman, S. J., Incze, P. F., Coffield, K. S., Borges, F. D., & Rukstalis, D. B. (2013). The prostatic urethral lift for the treatment of lower urinary tract symptoms associated with prostate enlargement due to benign prostatic hyperplasia: the L.I.F.T. Study. The Journal of urology, 190(6), 2161–2167. https://doi.org/10.1016/j.juro.2013.05.116
96	Romanov, A., Cherniavskiy, A., Novikova, N., Edemskiy, A., Ponomarev, D., Shabanov, V., Losik, D., Elesin, D., Stenin, I., Mikheenko, I., Zhizhov, R., Kretov, E., Pokushalov, E., Po, S. S., Martynyuk, T. V., & Steinberg, J. S. (2020). Pulmonary Artery Denervation for Patients with Residual Pulmonary Hypertension After Pulmonary Endarterectomy. Journal of the American College of Cardiology, 76(8), 916–926. https://doi.org/10.1016/j.jacc.2020.06.064
97	Roos, E. M., Hare, K. B., Nielsen, S. M., Christensen, R., & Lohmander, L. S. (2018). Better outcome from arthroscopic partial meniscectomy than skin incisions only? A sham-controlled randomised trial in patients aged 35-55 years with knee pain and an MRI-verified meniscal tear. BMJ open, 8(2), e019461. https://doi.org/10.1136/bmjopen-2017-019461
98	Rothstein, R., Filipi, C., Caca, K., Pruitt, R., Mergener, K., Torquati, A., Haber, G., Chen, Y., Chang, K., Wong, D., Deviere, J., Pleskow, D., Lightdale, C., Ades, A., Kozarek, R., Richards, W., & Lembo, A. (2006). Endoscopic full-thickness plication for the treatment of gastroesophageal reflux disease: A randomized, sham-controlled trial. Gastroenterology, 131(3), 704–712. https://doi.org/10.1053/j.gastro.2006.07.004
99	Ryösä, A., Kukkonen, J., Björnsson Hallgren, H. C., Moosmayer, S., Holmgren, T., Ranebo, M., Bøe, B., Äärimaa, V., & ACCURATE study group (2019). Acute Cuff Tear Repair Trial (ACCURATE): protocol for a multicentre, randomised, placebo-controlled trial on the efficacy of arthroscopic rotator cuff repair. BMJ open, 9(5), e025022. https://doi.org/10.1136/bmjopen-2018-025022
100	Salem, M., Rotevatn, S., Stavnes, S., Brekke, M., Vollset, S. E., & Nordrehaug, J. E. (2004). Usefulness and safety of percutaneous myocardial laser revascularization for refractory angina pectoris. The American journal of cardiology, 93(9), 1086–1091. https://doi.org/10.1016/j.amjcard.2004.01.032
101	Sarr, M. G., Billington, C. J., Brancatisano, R., Brancatisano, A., Toouli, J., Kow, L., Nguyen, N. T., Blackstone, R., Maher, J. W., Shikora, S., Reeds, D. N., Eagon, J. C., Wolfe, B. M., O'Rourke, R. W., Fujioka, K., Takata, M., Swain, J. M., Morton, J. M., Ikramuddin, S., Schweitzer, M., … EMPOWER Study Group (2012). The EMPOWER study: randomized, prospective, double-blind, multicenter trial of vagal blockade to induce weight loss in morbid obesity. Obesity surgery, 22(11), 1771–1782. https://doi.org/10.1007/s11695-012-0751-8
102	Schrøder, C. P., Skare, Ø., Reikerås, O., Mowinckel, P., & Brox, J. I. (2017). Sham surgery versus labral repair or biceps tenodesis for type II SLAP lesions of the shoulder: a three-armed randomised clinical trial. British journal of sports medicine, 51(24), 1759–1766. https://doi.org/10.1136/bjsports-2016-097098
103	Schwartz, M. P., Wellink, H., Gooszen, H. G., Conchillo, J. M., Samsom, M., & Smout, A. J. (2007). Endoscopic gastroplication for the treatment of gastro-oesophageal reflux disease: a randomised, sham-controlled trial. Gut, 56(1), 20–28. https://doi.org/10.1136/gut.2006.096842

For peer review only

104	Schweitzer, C., Brezin, A., Cochener, B., Monnet, D., Germain, C., Roseng, S., Sitta, R., Maillard, A., Hayes, N., Denis, P., Pisella, P. J., Benard, A., & FEMCAT study group (2020). Femtosecond laser-assisted versus phacoemulsification cataract surgery (FEMCAT): a multicentre participant-masked randomised superiority and cost-effectiveness trial. Lancet (London, England), 395(10219), 212–224. https://doi.org/10.1016/S0140-6736(19)32481-X
105	Scolapio, J. S., Gostout, C. J., Schroeder, K. W., Mahoney, D. W., & Lindor, K. D. (2001). Dysphagia without endoscopically evident disease: to dilate or not?. The American journal of gastroenterology, 96(2), 327–330. https://doi.org/10.1111/j.1572-0241.2001.03514.x
106	Shaheen, N. J., Sharma, P., Overholt, B. F., Wolfsen, H. C., Sampliner, R. E., Wang, K. K., Galanko, J. A., Bronner, M. P., Goldblum, J. R., Bennett, A. E., Jobe, B. A., Eisen, G. M., Fennerty, M. B., Hunter, J. G., Fleischer, D. E., Sharma, V. K., Hawes, R. H., Hoffman, B. J., Rothstein, R. I., Gordon, S. R., … Lightdale, C. J. (2009). Radiofrequency ablation in Barrett's esophagus with dysplasia. The New England journal of medicine, 360(22), 2277–2288. https://doi.org/10.1056/NEJMoa0808145
107	Sihvonen, R., Paavola, M., Malmivaara, A., Itälä, A., Joukainen, A., Nurmi, H., Kalske, J., Järvinen, T. L., & Finnish Degenerative Meniscal Lesion Study (FIDELITY) Group (2013). Arthroscopic partial meniscectomy versus sham surgery for a degenerative meniscal tear. The New England journal of medicine, 369(26), 2515–2524. https://doi.org/10.1056/NEJMoa1305189
108	Sikand, A., Ehmer, D. R., Jr, Stolovitzky, J. P., McDuffie, C. M., Mehendale, N., & Albritton, F. D., 4th (2019). In-office balloon sinus dilation versus medical therapy for recurrent acute rhinosinusitis: a randomized, placebo-controlled study. International forum of allergy & rhinology, 9(2), 140–148. https://doi.org/10.1002/alr.22248
109	Silverberg, G. D., Mayo, M., Saul, T., Fellmann, J., Carvalho, J., & McGuire, D. (2008). Continuous CSF drainage in AD: results of a double-blind, randomized, placebo-controlled study. Neurology, 71(3), 202–209. https://doi.org/10.1212/01.wnl.0000316197.04157.6f
110	Soylu Özler G. (2014). Silver nitrate cauterization: a treatment option for aphthous stomatitis. Journal of cranio-maxillo-facial surgery : official publication of the European Association for Cranio-Maxillo-Facial Surgery, 42(5), e281–e283. https://doi.org/10.1016/j.jcms.2013.10.006
111	Steward, D. L., Huntley, T. C., Woodson, B. T., & Surdulescu, V. (2008). Palate implants for obstructive sleep apnea: multi-institution, randomized, placebo-controlled study. Otolaryngology--head and neck surgery : official journal of American Academy of Otolaryngology-Head and Neck Surgery, 139(4), 506–510. https://doi.org/10.1016/j.otohns.2008.07.021
112	Stone, G. W., Teirstein, P. S., Rubenstein, R., Schmidt, D., Whitlow, P. L., Kosinski, E. J., Mishkel, G., & Power, J. A. (2002). A prospective, multicenter, randomized trial of percutaneous transmyocardial laser revascularization in patients with nonrecanalizable chronic total occlusions. Journal of the American College of Cardiology, 39(10), 1581–1587. https://doi.org/10.1016/s0735-1097(02)01829-6
113	Stuck, B. A., Sauter, A., Hörmann, K., Verse, T., & Maurer, J. T. (2005). Radiofrequency surgery of the soft palate in the treatment of snoring. A placebo-controlled trial. Sleep, 28(7), 847–850. https://doi.org/10.1093/sleep/28.7.847
114	Sullivan, S., Swain, J., Woodman, G., Edmundowicz, S., Hassanein, T., Shayani, V., Fang, J. C., Noar, M., Eid, G., English, W. J., Tariq, N., Larsen, M., Jonnalagadda, S. S., Riff, D. S., Ponce, J., Early, D., Volckmann, E., Ibele, A. R., Spann, M. D., Krishnan, K., … Pryor, A. (2018). Randomized sham-controlled trial of the 6-month swallowable gas-filled intragastric balloon system for weight loss. Surgery for obesity and related diseases: official journal of the American Society for Bariatric Surgery, 14(12), 1876–1889. https://doi.org/10.1016/j.soard.2018.09.486
115	Sutton, C. J., Ewen, S. P., Whitelaw, N., & Haines, P. (1994). Prospective, randomized, double-blind, controlled trial of laser laparoscopy in the treatment of pelvic pain associated with minimal, mild, and moderate endometriosis. Fertility and sterility, 62(4), 696–700. https://doi.org/10.1016/s0015-0282(16)56990-8

For peer review only

116	Swain, C. P., Bown, S. G., Storey, D. W., Kirkham, J. S., Northfield, T. C., & Salmon, P. R. (1981). Controlled trial of argon laser photocoagulation in bleeding peptic ulcers. Lancet (London, England), 2(8259), 1313–1316. https://doi.org/10.1016/s0140-6736(81)91340-4
117	Swain, C. P., Kirkham, J. S., Salmon, P. R., Bown, S. G., & Northfield, T. C. (1986). Controlled trial of Nd-YAG laser photocoagulation in bleeding peptic ulcers. Lancet (London, England), 1(8490), 1113–1117. https://doi.org/10.1016/s0140-6736(86)91835-0
118	Swank, D. J., Swank-Bordewijk, S. C., Hop, W. C., van Erp, W. F., Janssen, I. M., Bonjer, H. J., & Jeekel, J. (2003). Laparoscopic adhesiolysis in patients with chronic abdominal pain: a blinded randomised controlled multi-centre trial. Lancet (London, England), 361(9365), 1247–1251. https://doi.org/10.1016/s0140-6736(03)12979-0
119	Thompson, C. C., Chand, B., Chen, Y. K., DeMarco, D. C., Miller, L., Schweitzer, M., Rothstein, R. I., Lautz, D. B., Slattery, J., Ryan, M. B., Brethauer, S., Schauer, P., Mitchell, M. C., Starpoli, A., Haber, G. B., Catalano, M. F., Edmundowicz, S., Fagnant, A. M., Kaplan, L. M., & Roslin, M. S. (2013). Endoscopic suturing for transoral outlet reduction increases weight loss after Roux-en-Y gastric bypass surgery. Gastroenterology, 145(1), 129–137.e3. https://doi.org/10.1053/j.gastro.2013.04.002
120	Thomsen, J., Bretlau, P., Tos, M., & Johnsen, N. J. (1981). Ménière's disease: endolymphatic sac decompression compared with sham (placebo) decompression. Annals of the New York Academy of Sciences, 374, 820–830. https://doi.org/10.1111/j.1749-6632.1981.tb30922.x
121	Toouli, J., Roberts-Thomson, I. C., Kellow, J., Dowsett, J., Saccone, G. T., Evans, P., Jeans, P., Cox, M., Anderson, P., Worthley, C., Chan, Y., Shanks, N., & Craig, A. (2000). Manometry based randomised trial of endoscopic sphincterotomy for sphincter of Oddi dysfunction. Gut, 46(1), 98–102. https://doi.org/10.1136/gut.46.1.98
122	Vallon, A. G., Cotton, P. B., Laurence, B. H., Armengol Miro, J. R., & Salord Oses, J. C. (1981). Randomised trial of endoscopic argon laser photocoagulation in bleeding peptic ulcers. Gut, 22(3), 228–233. https://doi.org/10.1136/gut.22.3.228
123	van Schie, C. H., Whalley, A., Vileikyte, L., Wignall, T., Hollis, S., & Boulton, A. J. (2000). Efficacy of injected liquid silicone in the diabetic foot to reduce risk factors for ulceration: a randomized double-blind placebo-controlled trial. Diabetes care, 23(5), 634–638. https://doi.org/10.2337/diacare.23.5.634
124	Verheye, S., Jolicœur, E. M., Behan, M. W., Pettersson, T., Sainsbury, P., Hill, J., Vrolix, M., Agostoni, P., Engstrom, T., Labinaz, M., de Silva, R., Schwartz, M., Meyten, N., Uren, N. G., Doucet, S., Tanguay, J. F., Lindsay, S., Henry, T. D., White, C. J., Edelman, E. R., … Banai, S. (2015). Efficacy of a device to narrow the coronary sinus in refractory angina. The New England journal of medicine, 372(6), 519–527. https://doi.org/10.1056/NEJMoa1402556
125	Viswanathan, A., Vedantam, A., Williams, L. A., Koyyalagunta, D., Abdi, S., Dougherty, P. M., Mendoza, T., Bassett, R. L., Hou, P., & Bruera, E. (2020). Percutaneous Cordotomy for Pain Palliation in Advanced Cancer: A Randomized Clinical Trial Study Protocol. Neurosurgery, 87(2), 394–402. https://doi.org/10.1093/neuros/nyz527
126	Walega, D., McCormick, Z., Manning, D., & Avram, M. (2019). Radiofrequency ablation of genicular nerves prior to total knee replacement has no effect on postoperative pain outcomes: a prospective randomized sham-controlled trial with 6-month follow-up. Regional anesthesia and pain medicine, 44, 646-651. https://doi.org/10.1136/rapm-2018-100094
127	Wang, S., Lu, J., Li, Y. A., Zhou, H., Ni, W. F., Zhang, X. L., Zhu, S. P., Chen, B. B., Xu, H., Wang, X. Y., Xiao, J., Huang, H., Chi, Y. L., & Xu, H. Z. (2016). Autologous Olfactory Lamina Propria Transplantation for Chronic Spinal Cord Injury: Three-Year Follow-Up Outcomes from a Prospective Double-Blinded Clinical Trial. Cell transplantation, 25(1), 141–157. https://doi.org/10.3727/096368915X688065

For peer review only

128	Wei, J. T., Nygaard, I., Richter, H. E., Nager, C. W., Barber, M. D., Kenton, K., Amundsen, C. L., Schaffer, J., Meikle, S. F., Spino, C., & Pelvic Floor Disorders Network (2012). A midurethral sling to reduce incontinence after vaginal prolapse repair. The New England journal of medicine, 366(25), 2358–2367. https://doi.org/10.1056/NEJMoa1111967
129	Wood, D. E., Nader, D. A., Springmeyer, S. C., Elstad, M. R., Coxson, H. O., Chan, A., Rai, N. S., Mularski, R. A., Cooper, C. B., Wise, R. A., Jones, P. W., Mehta, A. C., Gonzalez, X., Sterman, D. H., & IBV Valve Trial Research Team (2014). The IBV Valve trial: a multicenter, randomized, double-blind trial of endobronchial therapy for severe emphysema. Journal of bronchology & interventional pulmonology, 21(4), 288–297. https://doi.org/10.1097/LBR.0000000000000110
130	Wu, P. I., Szczesniak, M. M., Maclean, J., Graham, P. H., Quon, H., Choo, L., & Cook, I. J. (2019). Endoscopic dilatation improves long-term dysphagia following head and neck cancer therapies: a randomized control trial. Diseases of the esophagus: official journal of the International Society for Diseases of the Esophagus, 32(6), doy087. https://doi.org/10.1093/dote/doy087
131	Zamboni, P., Tesio, L., Galimberti, S., Massacesi, L., Salvi, F., D'Alessandro, R., Cenni, P., Galeotti, R., Papini, D., D'Amico, R., Simi, S., Valsecchi, M. G., Filippini, G., & Brave Dreams Research Group (2018). Efficacy and Safety of Extracranial Vein Angioplasty in Multiple Sclerosis: A Randomized Clinical Trial. JAMA neurology, 75(1), 35–43. https://doi.org/10.1001/jamaneurol.2017.3825

For peer review only

PRISMA 2020 Checklist

Section and Topic	Item #	Checklist item	Location where item is reported
**TITLE**			
Title	1	Identify the report as a systematic review.	1
**ABSTRACT**			
Abstract	2	See the PRISMA 2020 for Abstracts checklist.	yes
**INTRODUCTION**			
Rationale	3	Describe the rationale for the review in the context of existing knowledge.	4
Objectives	4	Provide an explicit statement of the objective(s) or question(s) the review addresses.	4
**METHODS**			
Eligibility criteria	5	Specify the inclusion and exclusion criteria for the review and how studies were grouped for the syntheses.	5-6
Information sources	6	Specify all databases, registers, websites, organisations, reference lists and other sources searched or consulted to identify studies. Specify the date when each source was last searched or consulted.	5-6
Search strategy	7	Present the full search strategies for all databases, registers and websites, including any filters and limits used.	5
Selection process	8	Specify the methods used to decide whether a study met the inclusion criteria of the review, including how many reviewers screened each record and each report retrieved, whether they worked independently, and if applicable, details of automation tools used in the process.	5-6
Data collection process	9	Specify the methods used to collect data from reports, including how many reviewers collected data from each report, whether they worked independently, any processes for obtaining or confirming data from study investigators, and if applicable, details of automation tools used in the process.	6-7
Data items	10a	List and define all outcomes for which data were sought. Specify whether all results that were compatible with each outcome domain in each study were sought (e.g. for all measures, time points, analyses), and if not, the methods used to decide which results to collect.	6-7
	10b	List and define all other variables for which data were sought (e.g. participant and intervention characteristics, funding sources). Describe any assumptions made about any missing or unclear information.	6-7
Study risk of bias assessment	11	Specify the methods used to assess risk of bias in the included studies, including details of the tool(s) used, how many reviewers assessed each study and whether they worked independently, and if applicable, details of automation tools used in the process.	na
Effect measures	12	Specify for each outcome the effect measure(s) (e.g. risk ratio, mean difference) used in the synthesis or presentation of results.	na
Synthesis methods	13a	Describe the processes used to decide which studies were eligible for each synthesis (e.g. tabulating the study intervention characteristics and comparing against the planned groups for each synthesis (item #5)).	na
	13b	Describe any methods required to prepare the data for presentation or synthesis, such as handling of missing summary statistics, or data conversions.	na
	13c	Describe any methods used to tabulate or visually display results of individual studies and syntheses.	na
	13d	Describe any methods used to synthesize results and provide a rationale for the choice(s). If meta-analysis was performed, describe the model(s), method(s) to identify the presence and extent of statistical heterogeneity, and software package(s) used.	na
	13e	Describe any methods used to explore possible causes of heterogeneity among study results (e.g. subgroup analysis, meta-regression).	na
	13f	Describe any sensitivity analyses conducted to assess robustness of the synthesized results.	na
Reporting bias assessment	14	Describe any methods used to assess risk of bias due to missing results in a synthesis (arising from reporting biases).	na
Certainty	15	Describe any methods used to assess certainty (or confidence) in the body of evidence for an outcome.	na

For peer review only

PRISMA 2020 Checklist

Section and Topic	Item #	Checklist item	Location where item is reported
assessment			
**RESULTS**			
Study selection	16a	Describe the results of the search and selection process, from the number of records identified in the search to the number of studies included in the review, ideally using a flow diagram.	7
	16b	Cite studies that might appear to meet the inclusion criteria, but which were excluded, and explain why they were excluded.	7
Study characteristics	17	Cite each included study and present its characteristics.	8-10
Risk of bias in studies	18	Present assessments of risk of bias for each included study.	na
Results of individual studies	19	For all outcomes, present, for each study: (a) summary statistics for each group (where appropriate) and (b) an effect estimate and its precision (e.g. confidence/credible interval), ideally using structured tables or plots.	na
Results of syntheses	20a	For each synthesis, briefly summarise the characteristics and risk of bias among contributing studies.	na
	20b	Present results of all statistical syntheses conducted. If meta-analysis was done, present for each the summary estimate and its precision (e.g. confidence/credible interval) and measures of statistical heterogeneity. If comparing groups, describe the direction of the effect.	na
	20c	Present results of all investigations of possible causes of heterogeneity among study results.	na
	20d	Present results of all sensitivity analyses conducted to assess the robustness of the synthesized results.	na
Reporting biases	21	Present assessments of risk of bias due to missing results (arising from reporting biases) for each synthesis assessed.	na
Certainty of evidence	22	Present assessments of certainty (or confidence) in the body of evidence for each outcome assessed.	na
**DISCUSSION**			
Discussion	23a	Provide a general interpretation of the results in the context of other evidence.	10
	23b	Discuss any limitations of the evidence included in the review.	12
	23c	Discuss any limitations of the review processes used.	12
	23d	Discuss implications of the results for practice, policy, and future research.	13
**OTHER INFORMATION**			
Registration and protocol	24a	Provide registration information for the review, including register name and registration number, or state that the review was not registered.	4
	24b	Indicate where the review protocol can be accessed, or state that a protocol was not prepared.	4
	24c	Describe and explain any amendments to information provided at registration or in the protocol.	na
Support	25	Describe sources of financial or non-financial support for the review, and the role of the funders or sponsors in the review.	13
Competing interests	26	Declare any competing interests of review authors.	13
Availability of data, code and other materials	27	Report which of the following are publicly available and where they can be found: template data collection forms; data extracted from included studies; data used for all analyses; analytic code; any other materials used in the review.	Table 2 and na

For peer review only - http://bmjopen.bmj.com/site/about/guidelines.xhtml

From: Page MJ, McKenzie JE, Bossuyt PM, Boutron I, Hoffmann TC, Mulrow CD, et al. The PRISMA 2020 statement: an updated guideline for reporting systematic reviews. BMJ 2021;372:n71. doi: 10.1136/bmj.n71

For peer review only

PRISMA 2020 Checklist

For more information, visit: http://www.prisma-statement.org/

For peer review only
